# Considerations for developing complex post-stroke upper limb behavioural interventions: An international qualitative study

**DOI:** 10.1177/02692155241265271

**Published:** 2024-07-25

**Authors:** Matthew Wingfield, Gemma Hughes, Natalie A Fini, Amy Brodtmann, Gavin Williams, Kathryn S Hayward

**Affiliations:** 1 Department of Physiotherapy, University of Melbourne, Melbourne, Australia; 2 Department of Physiotherapy, Epworth Healthcare, Melbourne, Australia; 3 Department of Physiotherapy, Austin Health, Melbourne, Australia; 4 Department of Medicine, Monash University, Melbourne, Australia; 5 Department of Medicine (RMH), University of Melbourne, Melbourne, Australia

**Keywords:** Qualitative, upper limb, stroke rehabilitation, recovery, translational neuroscience

## Abstract

**Objective:**

To simultaneously explore the perspectives and opinions of different invested participant groups on the important considerations for development of upper limb behavioural interventions that drive optimal post-stroke upper limb motor recovery.

**Design:**

A qualitative descriptive study in a constructivist epistemology.

**Participants:**

Purposively selected participant groups (preclinical research *n* = 9, clinical research *n* = 9, clinical experience *n* = 9 and lived experience *n* = 10).

**Setting:**

Research participants were selected from top internationally published authors. Experiential participants were recruited internationally, through networks.

**Results:**

Four themes were identified with embedded subthemes. Theme 1: ‘Clinical relevance should be the core of a “good” research question’ with two subthemes: ‘Breaking down silos: forging interdisciplinary research teams’, and ‘Beyond the pipeline: bench to bedside and back’; theme 2: ‘Balance restitution and compensation to maximise outcomes’ with three subthemes: ‘Good outcome: going beyond an outcome measure’, ‘Recovery is a puzzle: measure all the pieces’, and ‘Optimising capacity: knowing when and how’; theme 3: ‘Stroke demands personalised solutions’ with two subthemes: ‘Condition-specific considerations’, and ‘Person-specific considerations’; theme 4: ‘Upper limb recovery requires complex interventions’ with four subthemes: ‘Fuelling engagement’, ‘Content is crucial’, ‘Multidimensional dose’, and ‘Therapist sway’.

**Conclusions:**

This study suggests that post-stroke upper limb motor interventions are the interactions of multiple intervention elements (e.g. dose and content) shaped by different contextual considerations (e.g. stroke and personal factors). Development of such interventions may need to consider both content and context of the intervention to drive optimal recovery.

## Introduction

As many as half of all stroke survivors experience upper limb motor impairment early after stroke.^1,2^ Behavioural interventions are currently the only treatment options available to drive recovery in stroke survivors whose upper limb impairment persists for months and years post-stroke,^3^ leading to its recognition as a priority research area.^4,5^

Researchers are encouraged to progress testing of new interventions systematically along a continuum from preclinical research in the laboratory through to implementation in clinical practice^6,7^ to ultimately impact health outcomes. There are two potential translation blocks along this research continuum: preclinical research to early-phase clinical trials, and late-phase clinical trials to clinical practice.^7^ Preclinical research, clinical research, clinical experience and lived experience groups often contribute evidentiary input isolated to their domain when developing post-stroke upper limb behavioural interventions.^8–10^ These groups rarely contribute simultaneously during the development of behavioural interventions for stroke recovery. Incorporating all perspectives (preclinical and clinical research, clinical and lived experience) may enable the field to better navigate potential translation blocks from preclinical research to early-phase clinical trials,^11,12^ as well as late-phase clinical trials to clinical practice.^13^ A pluralist approach^14^ to behavioural intervention development may leverage the unique knowledge of each group to advance our collective understanding of the requirements for post-stroke upper limb behavioural interventions across the continuum of research.

Behavioural interventions for post-stroke upper limb motor recovery are complex.^15^ Behavioural interventions combine multiple ingredients or intervention elements (such as dose^16–18^ and content^8^). It is plausible that the interaction between these elements may be responsible for driving recovery.^19,20^ The development of behavioural interventions may be further complicated by contextual considerations such as the characteristics of the person undertaking a behavioural intervention or how long post-stroke an intervention is delivered.^21^ Identifying broader contextual considerations for upper limb behavioural intervention development, like their intended purpose, may provide the field with a deeper understanding of what influences post-stroke upper limb motor recovery. Therefore, our aim was to simultaneously explore the perspectives and opinions of people with experience in preclinical research, clinical research, clinical experience and lived experience on the important considerations for the development of upper limb behavioural interventions to drive optimal post-stroke upper limb motor recovery.

## Methods

Ethical approval was obtained from the University of Melbourne [2022-22956-24818-4]. This study was conducted per the declaration of Helsinki^22^ and The National Statement on Ethical Conduct of Human Research.^23^ This study is reported in accordance with the consolidated criteria for reporting qualitative research.^24^

### Research team and reflexivity

All interviews were conducted by the same author (MW), a cis-male senior clinician (physiotherapist) and PhD candidate. The interviewer had previously worked as a clinician with three participants enrolled in this study. Some other participants had contributed to research projects with authors KSH, AB, GW and NAF; however, none had an existing relationship with the interviewer. All participants were provided with a participant information and consent form which outlined the research questions. No prior contact had been made with participants regarding this study. Authors KSH, NAF, GW and AB had prior qualitative research experience.^25–28^

### Study design

A qualitative descriptive content analysis of multiple semi-structured interviews informed the results of this study.^29,30^ Findings are presented as an amalgamation of the multiple perspectives of the participants. We considered the findings of this research to be constructed through the collective knowledge of each participant group^31^ and not identified through discovery. Therefore, a constructivist epistemological approach was adopted. We considered each participant group to possess unique, fundamental knowledge that held equal value and importance when forming our results, which aligns with a philosophy of scientific pluralism.^14^

Four international participant groups were purposively targeted based on unique inclusion criteria aligned with their expertise: (1) preclinical research, (2) clinical research, (3) clinical experience and (4) lived experience. Attempts were made to balance all groups for geographical location and gender. Preclinical and clinical research participants were identified among the top 20 authors from a Scopus search using keywords (unpublished to maintain participant confidentiality), with consideration of scientific background (e.g. basic science, medical or allied health). Clinical experience and lived experience participants were identified through the authorship group's knowledge and networks. Clinical experience participants required more than seven years of experience working in post-stroke upper limb rehabilitation, with consideration given to discipline (e.g. physiotherapist or occupational therapist). Lived experience participants were adults (>18 years of age) with more than six months history of post-stroke upper limb motor impairment. All potential participants were emailed an invitation with the group-specific participant information and consent form. One follow-up email was provided if no response was received. It was deemed that an individual was not interested in participating after the follow-up email if they did not officially decline. Four participants declined to participate (see Supplemental 1). Snowballing was used to recruit additional experiential participants. The sample size was determined by thematic saturation. Thematic saturation was considered achieved when no new themes were identified across three consecutive interviews within a single participant group.

### Data collection

A pre-defined interview guide (Supplemental 2) directed each interview. Participants were not provided with the interview guide ahead of the interview. Included participants participated in a single interview. Participant demographics are presented in Table 1. The interview guide focused on three key areas: the participant's (1) role in intervention development, (2) understanding of the context of upper limb recovery and (3) understanding of elements in and neuroscientific rationale for upper limb behavioural interventions. The authors designed the interview guide with input from people with expertise in each of the four participant groups (preclinical research, clinical research, clinical experience and lived experience). The guide was refined for clarity and was piloted with two researchers (neurological rehabilitation), two clinicians (neurological rehabilitation) and one stroke survivor. All interviews were conducted and recorded on *Zoom* (versions 5.15.0–5.12.7) and audio files were used for transcription. The interviewer made field notes during each interview using Microsoft Word (version 16.0.16501.20228). Interviews took place in an environment self-selected by the participant (usually work or home). Participants in the lived experience group were invited to have a support person present during the interview. Interviews lasted between 22 and 60 min. Each recording was transcribed verbatim by a professional transcription service (Pacific Transcription Services Australia). Transcripts were analysed as the study progressed to monitor for saturation. Transcripts were not returned to participants for comment.

**Table 1.** Participant demographics via location, research background and gender.

	Preclinical research *n* = 9	Clinical research *n* = 9	Clinical experience *n* = 9	Lived experience *n* = 10
Continent, *n*				
North America	7	5	2	0
Europe	2	3	3	1
Australasia	0	1	3	8
Asia	0	0	1	1
Gender, *n*				
Male	8	4	3	5
Female	1	5	6	5
Species researched*, *n*				
Rodents	5	n/a	n/a	n/a
Non-human primates	2	n/a	n/a	n/a
Humans	4	9	n/a	n/a
Background training*, *n*				
Neuroscience/science	9	7	0	n/a
Medicine or health sciences	4	9	9	n/a
Age range	n/a	n/a	n/a	33–79
Time since stroke (months)	n/a	n/a	n/a	7–144

n/a = not applicable.

*Participants could qualify in multiple categories.

### Analysis and findings

Three authors (MW, GH and KSH) read all transcripts by interview group. *NVIVO* software (version 20.7.1.1534) was used to code the data. One author (MW) coded all transcripts and created a coding tree (see Supplemental 3). An independent author (GH) verified coding using the coding tree. Coding disagreements were resolved through discussion, and a third author (KSH) provided input to achieve consensus if required. Two researchers (MW and KSH) derived themes from the data.

A total of 37 interviews were conducted: preclinical research *n* = 9 (median interview length 42 min), clinical research *n* = 9 (median interview length 43 min), clinician *n* = 9 (median interview length 49 min) and stroke survivor *n* = 10 (median interview length 31 min). Major themes with embedded subthemes are presented in the results. Participants were not approached for feedback on themes. Each group's themes were subsequently analysed across groups to identify consistencies and discrepancies to define study-level themes.

## Results

### Theme 1. Clinical relevance should be the core of a ‘good’ research question

Preclinical and clinical research group participants identified a primary role in formulating research questions. They discussed that a research question needs to be able to be investigated with rigorous methodology, produce an answer, and be relevant to a clinical problem.

I try to start with the clinical problem … my preference is really to start with the clinical question and work my way back. Preclinical Research #9

**Figure 1.**
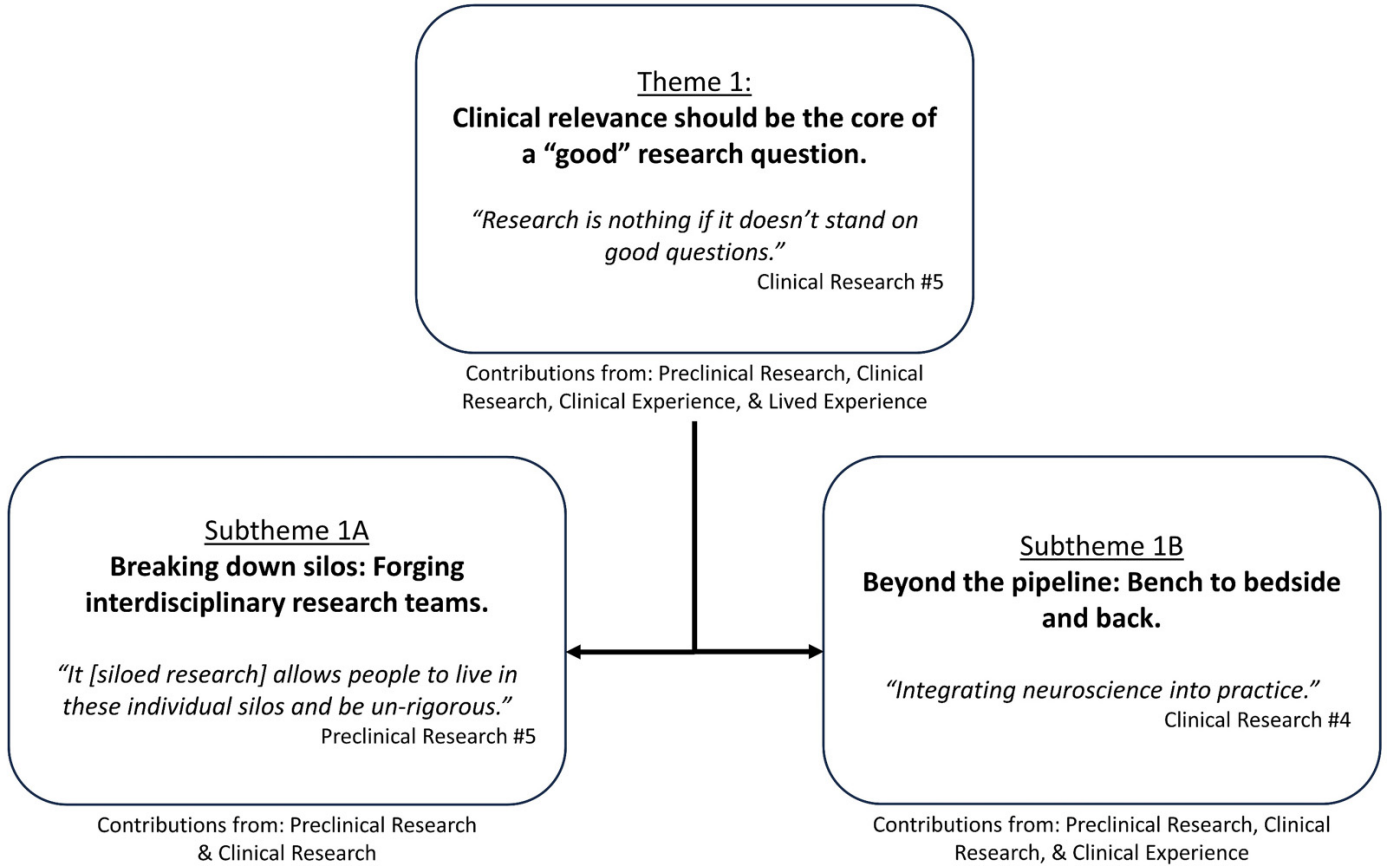
Theme 1: ‘Clinical relevance should be the core of a ‘good’ research question’ overview with embedded subthemes.

Participants in the clinical and lived experience groups identified that they were uniquely placed to communicate clinical problems. Participants in the clinical experience group identified their strengths in highlighting problems faced by stroke survivors at a population level. Participants in the lived experience group identified their strengths in highlighting problems faced by stroke survivors at an individual level. All groups agreed that co-design was the most practical way to ensure research relevance. While all groups identified stroke survivors as essential to co-design, only one interviewee beyond the clinical experience group suggested co-design should include clinicians. Supporting quotes can be found in Figure 1; additional quotes in Supplemental 4.

### Subtheme 1A. Breaking down silos: forging interdisciplinary research teams

Participants in the preclinical and clinical research groups identified the existence of silos within stroke recovery research. Silos were described as researchers from a specific discipline (e.g. basic science) undertaking research in a fixed area (e.g. preclinical). The existence of silos was viewed to limit the opportunity to conduct co-designed research or forge teams with researchers from multiple disciplines. Siloed research was perceived to contribute to misinterpretation and inherent biases in stroke recovery research. Breaking down silos was suggested as a pathway to more efficient communication, improved translation, and reduced research waste.

We have inherent biases, and we make assumptions about things. So, you really need everything to work together to advance the field … your interpretation as a researcher, as a clinician, can go awry. Clinical Research #9

Participants in the preclinical and clinical research groups shared the perspective that clinical problems may best be addressed by developing multiple research questions, each tackled by bespoke research teams. Participants suggested that members of a research team should be selected based on the knowledge and skills required to answer the proposed question. An optimistic future for stroke recovery research was expressed, where researchers from different disciplines formed teams to rigorously address research questions, making siloed research a relic of the past.

### Subtheme 1B. Beyond the pipeline: bench to bedside and back

Participants in the preclinical research, clinical research and clinical experience groups described intervention development as a pipeline. Knowledge gained from investigating a research question should inform the formulation of subsequent enquiry. This pipeline was described to be largely unidirectional (i.e. from researchers to clinicians); however, participants suggested it should be bi-directional. Researchers highlighted the need to work across both preclinical and clinical fields to enhance understanding and provide valuable insights to further intervention development.

Participants in the clinical research and clinical experience groups shared the view that research should be interpreted in the context of how it was conducted. They viewed research as covering everything from conceptual work (proof of principle) all the way through to implementation. They warned against attempting to implement conceptual work into clinical practice without supporting safety, feasibility, efficacy and effectiveness evidence. They encouraged practising clinicians to always come back to the purpose defined within the research question.

How does that impact on the way we actually treat patients, is it a useful thing to think about? Clinical Research #5

### Theme 2. Balance restitution and compensation to maximise outcomes

Participants in the preclinical and clinical research groups suggested that neurological damage caused by stroke necessitates the nervous system to adapt and compensate. This neurological adaptation was considered a compensatory process and part of post-stroke neuroplasticity. Neurological adaptations were viewed to manifest compensatory behavioural performance, which could be identified when examined with high enough resolution. Clinical research and clinical experience participants identified and contrasted two distinct compensation approaches: compensation that enables impairment reduction and compensation that comes at the cost of impairment reduction.

All groups identified the ideal outcome for animal or human stroke survivors as complete resolution of upper limb impairment to pre-stroke levels. However, they emphasised that a return to pre-stroke function was aspirational in the context of current practice.

Good recovery is better not perfect … I think that's unrealistic: to expect that every patient is going to return to their normal function after having an injury to their brain. Clinical Experience #9

**Figure 2.**
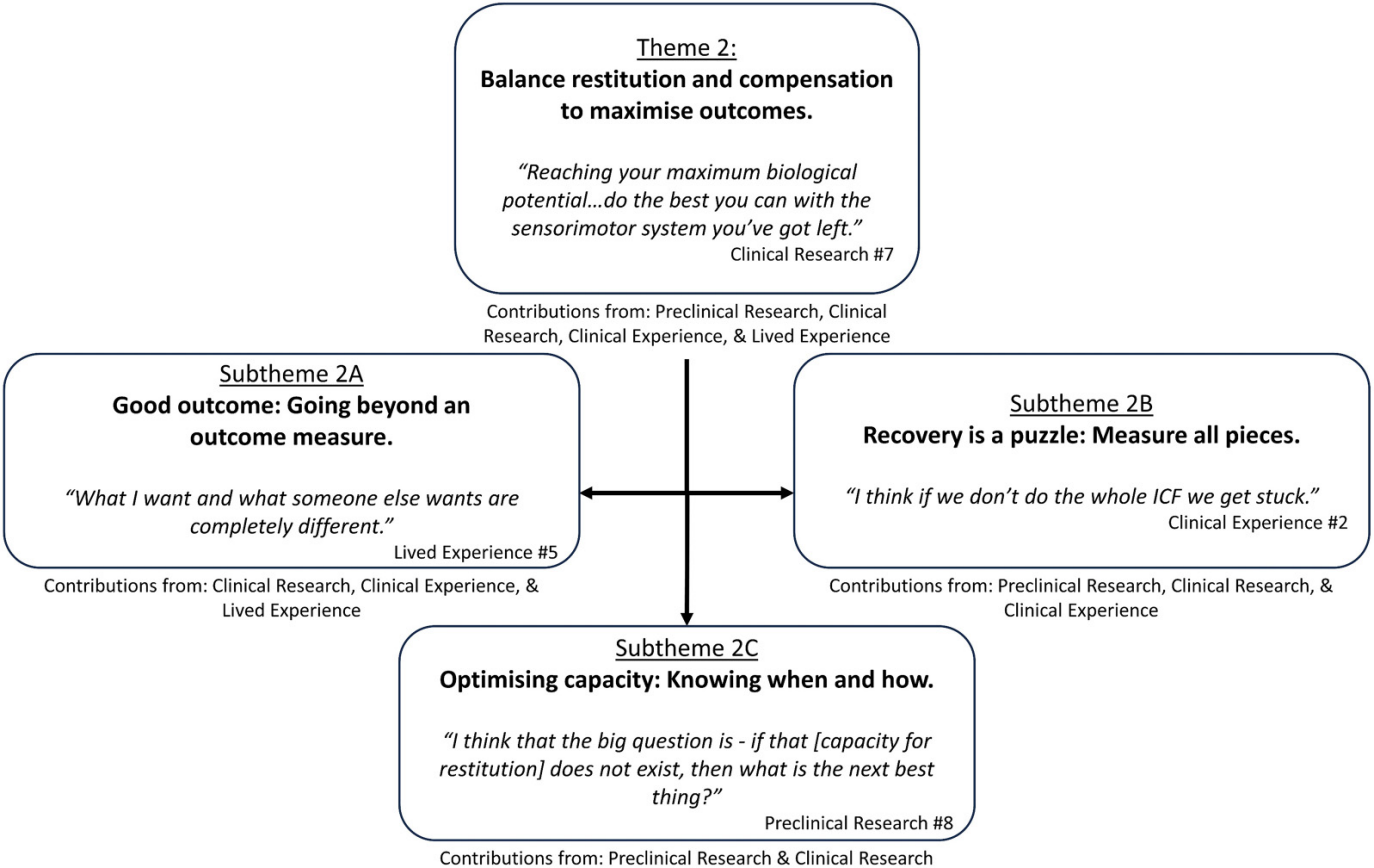
Theme 2: ‘Balance restitution and compensation to maximise outcomes’ overview with embedded subthemes.

Participants in all groups defined upper limb recovery by the stroke survivor's behavioural performance. Participants in the clinical research, clinical and lived experience groups suggested that improvement in performance through compensatory behaviours in the direction of normal should be considered part of recovery and, at times, a positive outcome. Supporting quotes can be found in Figure 2; additional quotes in Supplemental 5.

### Subtheme 2A. Good outcome: going beyond an outcome measure

Participants in the clinical research, clinical and lived experience groups challenged the idea that ‘good recovery’ aligns with any particular outcome or threshold in the World Health Organization's International Classification of Functioning and Disability (WHO ICF)^32^ impairment or activity domains. Participants distinguished the concepts of recovery and outcome. Outcome was considered to describe an endpoint. Recovery was considered to describe the process of improvement made by a stroke survivor without a defined end point. Participants from all groups suggested what a stroke survivor could do in the real-world (i.e. participation) should define a good or poor outcome. Participants in the lived experience group highlighted that a perception of a ‘good outcome’ differs from individual to individual.

They’re not recovered … they’re six points better on the scale and that's great, but they’re still moderately affected and there's still a tonne of things they can’t do. Clinical Research #1

### Subtheme 2B. Recovery is a puzzle: measure all pieces

Preclinical research, clinical research and clinical experience participants discussed recovery in reference to multiple behavioural domains of the WHO ICF.^32^ Improvements in body structure and function (i.e. impairment) were considered paramount to behavioural restitution. However, participants in the lived experience group emphasised the importance of the activity and participation domains over impairment. Participants in all groups discussed improvement across all domains as an ideal outcome.

Participants in the preclinical research, clinical research and clinical experience groups suggested that the effects of an intervention should be measured by examining multiple domains of the WHO ICF. The different rate and timeframe over which impairment, activity and participation recovery occur was acknowledged as a challenge when examining all domains simultaneously. Kinematic measures were thought to best capture nuanced impairment change. No participant proposed an ideal approach to measure multiple domains simultaneously.

Well-established outcome measures are confusing recovery and compensation. Clinically we have a better handle on disassociating those most of the time. But ground proof lies in kinematics. Preclinical Research #1

### Subtheme 2C. Optimising capacity: knowing when and how

Participants in the preclinical and clinical research groups identified the importance of timing interventions with an optimised neurobiological state or sensitive period of neuroplasticity. Timing was perceived to be of particular importance when the goal of rehabilitation was impairment reduction. Participants in the preclinical research group discussed sensitive periods of neuroplasticity as ‘almost certain’ to exist in humans. However, many stated that the stroke recovery field is yet to understand when these periods occur in humans or the variability between individual stroke survivors.

Participants in the preclinical research group shared that behavioural interventions should optimise the use of residual neurological structures involved in pre-stroke upper limb movement (i.e. ipsilesional corticospinal tract). In the context of catastrophic corticospinal tract damage, recovery of motor impairment may not be possible. In such circumstances, the goal of a behavioural intervention may need to change to maximise activity and participation. A key area for scientific exploration was identified as whether behavioural interventions targeting alternate neurological structures (e.g. reticulospinal tract) can help improve outcomes. They suggested that effective behavioural interventions may capitalise on undamaged neural structures while facilitating recovery of damaged structures.

Get the best recovery with the remaining circuitry that you still have … we really need to maximise whatever corticospinal drive we have left. That's my message. Preclinical Research #5

### Theme 3. Stroke demands personalised solutions

Participants in all groups considered personalisation of behavioural interventions to be essential due to the heterogeneity of post-stroke phenotypes. Participants in the preclinical group identified that variability is seen in preclinical models of stroke with similar stroke lesions (i.e. volume, size, location). They noted that such variability is likely to be more prominent in clinical populations, with more advanced brain structures and distributed neural networks.

It always has impressed me when we started making even these little focal strokes … we see a wide disparity in the way that animals react, whether it's rats, monkeys, or humans. Preclinical Research #9

**Figure 3.**
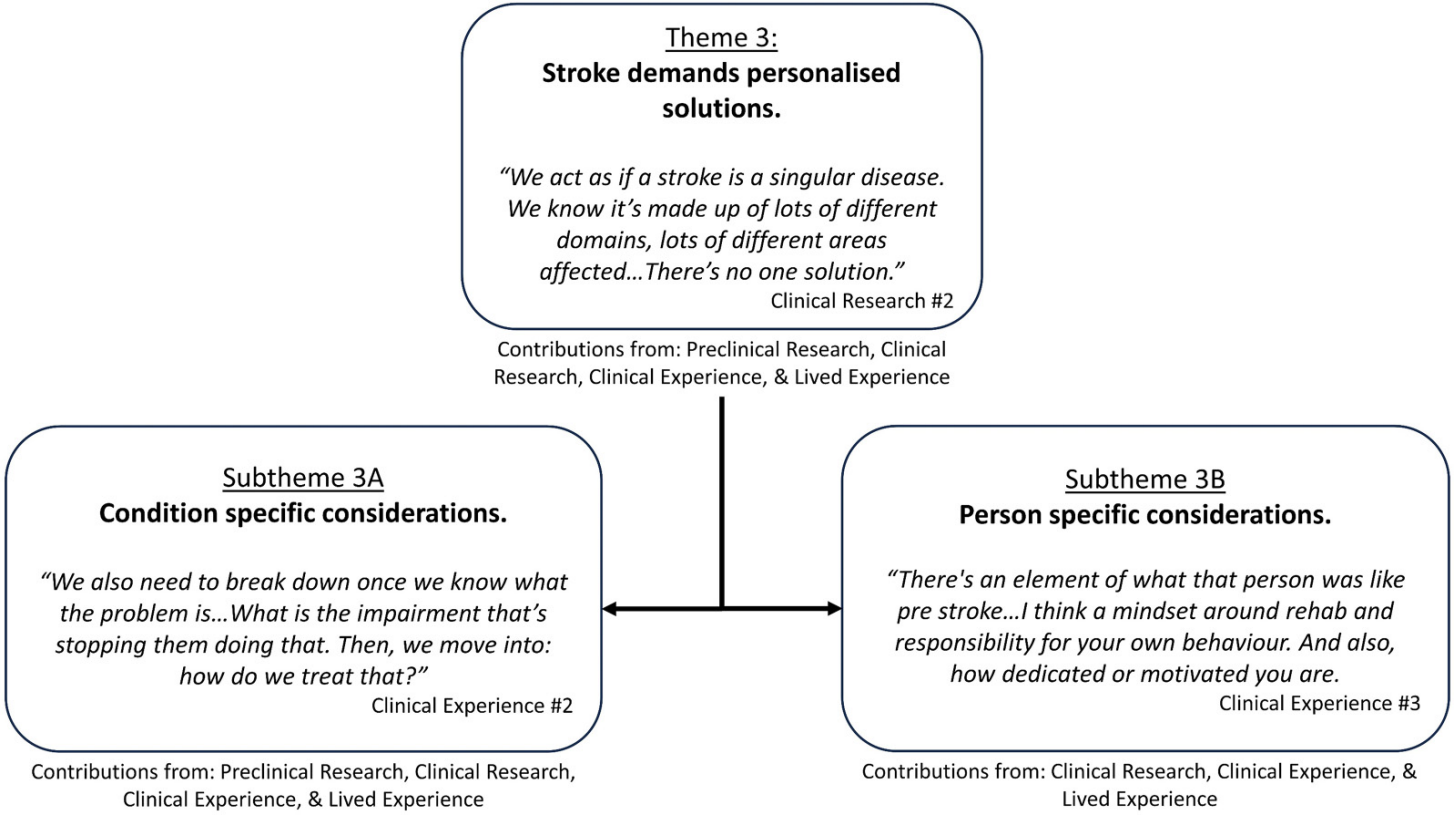
Theme 3: ‘Stroke demands personalised solutions’ overview and embedded subthemes.

Participants in the preclinical and clinical research groups suggested that those developing behavioural interventions need to consider the heterogeneity of post-stroke presentations while maintaining a firm foundation in neuroscience and human biomechanics. Supporting quotes can be found in Figure 3; additional quotes in Supplemental 6.

### Subtheme 3A. Condition-specific considerations

All groups identified that a stroke survivor's presentation may include multiple impairments that together define a behavioural phenotype (e.g. contributions from hemiparesis, abnormal muscle synergies, loss of dexterity). Participants in the preclinical research, clinical research and clinical experience groups described the need for diagnostic tools to provide deep insights into a stroke survivor's phenotype and go beyond what is possible to capture with a bedside clinical assessment. Phenotypes were discussed to be characterised by behavioural presentation (e.g. weakness) and biomarkers of neurological structures and function (e.g. residual corticospinal tract integrity). Integrating both behavioural presentation and biomarkers to define a stroke phenotype would be considered an advancement that would subsequently inform intervention decisions.

There could be no doubt that correlates of better outcome in the mild patients are not the same as the correlates in the more severe patients. There's no one solution. Clinical Research #2

Participants in the preclinical research, clinical research and clinical experience groups discussed the need to personalise interventions to consider the potential consequences of non-motor impairments to upper limb motor recovery.

### Subtheme 3B. Person-specific considerations

Participants in the clinical research, clinical and lived experience groups raised the importance of personalising the behavioural intervention content to the goals of the stroke survivor. They acknowledged an interplay between the goal-directed nature of rehabilitation and engagement in an intervention.

My big picture would be to be able to ride a bike again because I was an avid cyclist before my stroke. So, to be able to hold handlebars and ride…That would be good recovery. Lived Experience #5

Preclinical and clinical research participants discussed the importance of premorbid health and age in personalising interventions. Stroke survivors with significant comorbid illness were said to be less likely to be offered or receive intense interventions, thus compounding a poorer prognosis.

### Theme 4. Upper limb recovery requires complex interventions

Participants in the preclinical research and clinical research groups identified complex interventions reflected the combination of multiple overlapping and interacting intervention elements. Four intervention elements were considered by participants: engagement, content, dose and therapist.

You put all those elements together, it's kind of a complicated soup, but I think they’re all really important for the whatever-it-is you’re testing to be successful. Clinical Research #3

**Figure 4.**
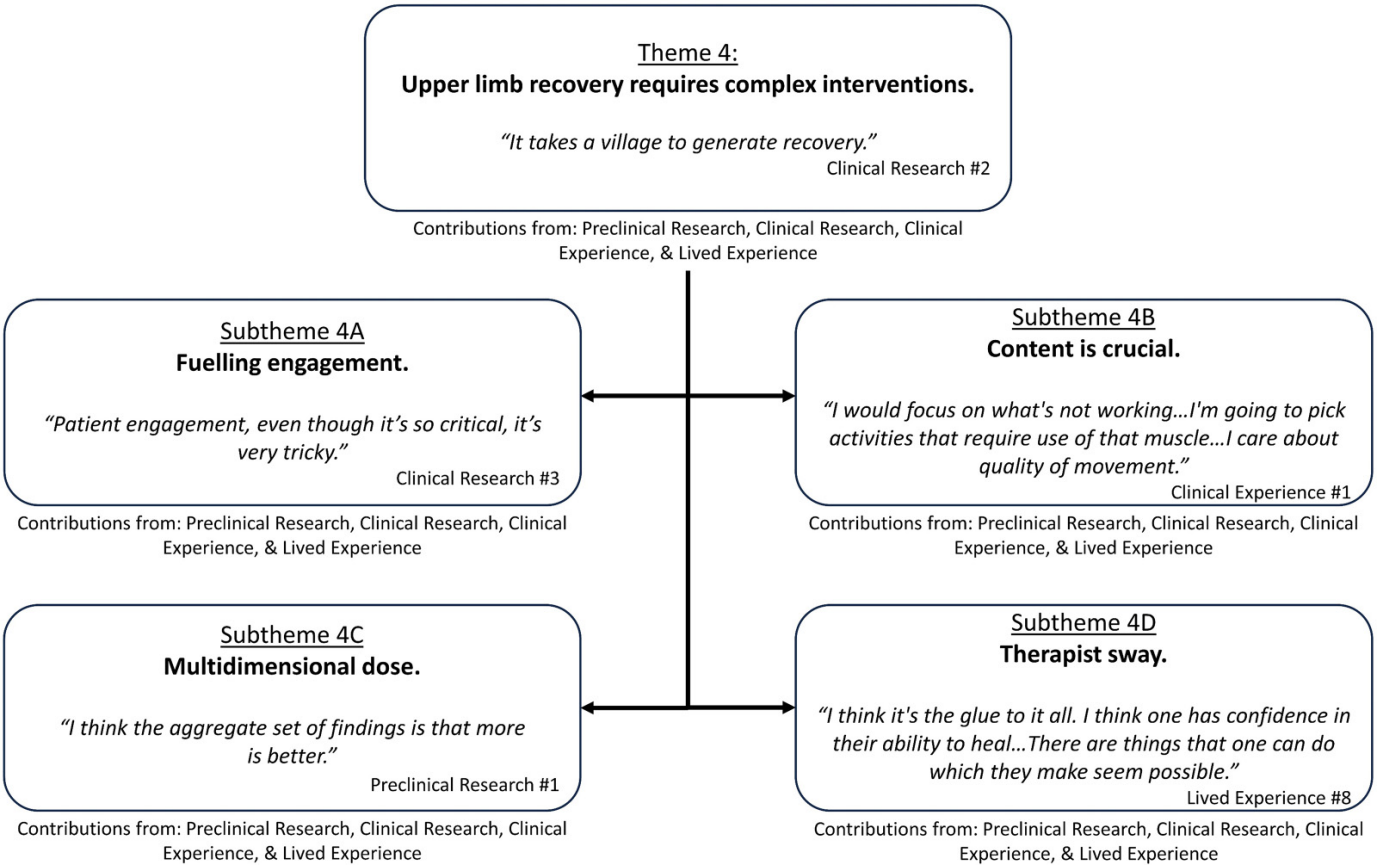
Theme 4: ‘Upper limb recovery requires complex interventions’ overview with embedded subthemes.

Participants in the preclinical research group reflected upon examples of when complex interventions had been experimentally tested. The effects of complex interventions (e.g. enriched rehabilitation which is reach training plus an enriched environment) were identified as ‘almost always’ generating greater improvement than more simplified interventions (e.g. reach training alone). Continued exploration of individual intervention elements in isolation was described as unlikely to provide the field with a breakthrough in improved recovery for stroke survivors. Supporting quotes can be found in Figure 4; additional quotes in Supplemental 7.

### Subtheme 4A. Fuelling engagement

Participants from all groups identified the importance of the stroke survivor's engagement in an intervention. Reward, feedback, support and socialisation, education, accessibility and hope were identified as potential considerations that affect engagement.

All groups identified that there needed to be a rethink of interventions that motivate stroke survivors to stay on task. Rewards were considered underexplored in human stroke recovery. Rewards were described to be physiologically different from external encouragement (i.e. therapist, family member) and separate from one's intrinsic motivation.

To use an American term: ‘atta boy’ or ‘atta girl’ the kind of therapist-based positive verbal reinforcement. That's just not enough. Preclinical Participant #1

All groups suggested feedback was key to keep a stroke survivor engaged in an intervention. Those in the preclinical research group emphasised that feedback plays an important role in learning and relearning skill-based movement. Those in the lived experience group identified feedback was required to understand if they had executed movement correctly. Participants in the clinical experience group identified the importance of providing knowledge of performance to ensure adequate movement quality was achieved. Further, they identified the importance of providing knowledge of results to reinforce task success.

All groups identified the importance of support and socialisation to remain engaged with an intervention. Participants in the preclinical group made analogies to enriched environments used in rodent models to highlight the importance of social stimulation. Participants in the lived experience group identified the importance that the support of a loved one or a therapist played in keeping them engaged long-term in their rehabilitation programs. All groups suggested that interventions should balance socialisation and time on task.

Something that we don’t measure much that's huge to normal function and post-stroke function is the social circle. Clinical Research #2

Education was identified as important to upper limb motor recovery by participants in the clinical research, clinical experience and lived experience groups. Education may be used to inform the participant of specific foci during a behavioural intervention, e.g. different components of a movement to be practised. Participants in the lived experience group identified that understanding the purpose of an exercise was critical to remaining engaged.

Being able to explain what one is doing and why, that's something very important. Lived Experience #8

Accessibility to therapists was identified as key to maintaining engagement and adherence by participants from the clinical research, clinical and lived experience groups. Stroke survivors identified ongoing access to behavioural interventions as essential to drive recovery. Participants in the clinical research, clinical and lived experience groups identified that practice of some parts of a behavioural intervention should be able to be replicated without a therapist.

Participants in the clinical and lived experience groups identified the importance of hope in maintaining engagement with an intervention. Clinical experience group participants described a nuanced version of hope, where unrealistic goals should be compartmentalised into smaller achievable goals with the stroke survivor. Lived experience participants stated firmly that they disliked being told about the potential ceiling of their post-stroke upper limb function. They instead sought encouragement to come to peace with their disability on their terms.

All I want from my professional viewpoint is what's realistic, realistic hope based on what's happening … I’m not asking to make judgements. Lived Experience #2

### Subtheme 4B. Content is crucial

The content of a behavioural intervention was identified as crucial by participants from all groups. Considerations under the content subtheme were perceived to be ‘what is done’, intervention adjuvants, specificity and transference. ‘What is done’ was said to incorporate what actions were completed by the patient and the therapist, how they are completed, and what equipment was used.

Movement quality was perceived as an important driver of post-stroke upper limb recovery by participants in all groups. Those in the preclinical research group identified quality as practicing how the upper limb normally moves during functional tasks (i.e. normal movement patterns). Clinical experience group participants identified that normal movement patterns could be broken down and individual movements isolated for part practice. These individual movements could be trained to recover the missing pieces of a normal movement pattern.

I would focus on what's not working in their impairments … I care about quality of movement. Clinical Experience Participant #1

Participants in the preclinical, clinical research and clinical experience groups discussed adjuvants designed to increase the potential benefit derived from a behavioural intervention. Participants in the preclinical and clinical research groups identified pharmacological options, transcranial magnetic stimulation and implantable stimulation devices as potential adjuvants. Participants in the clinical experience group identified adjuvants as primers of the nervous system before the active content of the intervention. Participants in the preclinical and clinical research groups emphasised that adjuvants must be combined with behavioural interventions and should not be considered interventions in isolation.

Participants in all groups discussed specificity in training and transference to everyday life. Preclinical research, clinical research and clinical experience group participants discussed the importance of practising specific movements contributing to multiple skills. Participants suggested that a skill recovered in a specific context should be transferred into real-world scenarios.

### Subtheme 4C. Multidimensional dose

Participants in all groups identified the importance of multiple different dimensions of dose to post-stroke upper limb motor recovery. Participants identified amount of therapy, difficulty of task and intensity of practice as important drivers of post-stroke upper limb motor recovery.

All groups identified the importance of a large amount of practice. Participants in the preclinical research group discussed animal studies demonstrating that large amounts of training were essential for motor skill learning. These participants mentioned repetitions, volume of time in a therapy space, and volume of time on task as potential quantifiable metrics of the amount of an intervention.

Participants in the preclinical research, clinical research and clinical experience groups all identified difficulty as an important consideration of dose. Participants in the preclinical research group emphasised the importance of a task being on the edge of one's capacity. Maintaining optimal difficulty (or challenge point) ensures that the behavioural intervention sufficiently activates and engages the neural circuitry involved in motor recovery. Exercises that stroke survivors could easily complete were considered redundant as they would not engage sufficient neural circuitry to drive recovery.

Make it progressive or regressive … we can’t have a fixed point and we probably can’t have the same point for everyone. Clinical Research #1

Participants in the preclinical and clinical research groups identified intensity as an important consideration of dose. However, there was no consensus on what was meant by intensity.

### Subtheme 4D. Therapist sway

Participants in all groups identified that a therapist had the power to make differences in a stroke survivor's recovery. Two considerations were discussed to influence the therapist's impact on recovery: relationship with the stroke survivor and level of knowledge and skill.

Participants in the clinical research, clinical and lived experience groups identified the relationship between the clinician and the stroke survivor, termed here as the therapeutic alliance, as essential. Participants in the lived experience group described their need to find a clinician they connected with to assist them with their recovery. Optimal therapeutic alliance was described as dynamic, where the clinician could adjust their approach to engagement, content and dose of an intervention on a given day to support a stroke survivor to get the most out of their therapy. All elements of an intervention were said to rely on a foundation of therapeutic alliance.

Participants in all groups identified that a therapist's skill and knowledge play a role in recovery. Some skills and knowledge were explicit, such as knowledge of normal human movement kinetics and kinematics. Some were implicit, such as what components of a reach movement would be acceptable to allow compensation for, which in turn allows impairment reduction in a different movement component.

There are some therapists who are just remarkably good with their hands. You give them a patient, you can pretty much guarantee that that patient is going to make improvements. Clinical Research #9

## Discussion

All participants suggested that complex interventions with multiple overlapping and interacting intervention elements will be required to optimise upper limb recovery. Intervention elements (engagement, content, dose and therapist) have multiple dimensions requiring consideration when developing complex post-stroke upper limb behavioural interventions. Several contextual factors were identified for behavioural intervention development, including the purpose (research question to be addressed), goal (restitution or compensation focused) and intended recipient (personalisation requirements).

The perception of what was a favourable post-stroke upper limb motor outcome varied depending on the participant group and the lens through which they examined recovery. Lived experience participants prioritised outcomes in the domains of activity and participation, while preclinical research participants prioritised impairment. These perspectives align with those previously reported for stroke survivors^10^ and researchers.^33,34^ Therefore, primary outcome measures should be selected dependent on which WHO ICF^32^ domain(s) is/are expected to improve,^35^ consistent with the Stroke Recovery and Rehabilitation Roundtable recommendations for multidomain measurement.^36^ All participant groups emphasised inclusion of real-world performance via participation measures.

Complex interventions in human stroke recovery research remain underexamined.^19^ All participant groups identified that complex interventions contain multiple interacting intervention elements, in keeping with previous stroke recovery research.^37^ A foundation of this study is that each participant group's input was considered equal when identifying the different intervention elements. Further research into the relative importance that each intervention element may play in promoting recovery is important. We identified that intervention element prioritisation may change based on the context of the research question, the desired outcome (restitution or compensation), and the stroke survivor's presentation. Previous stroke recovery research has highlighted the need to shift from a one-size-fits-all approach to personalised and tailored behavioural interventions^38,39^ via manipulation of intervention elements in response to contextual factors.

There were two novel aspects of our study. Firstly, we targeted recruitment of participants whose experience covered the continuum of research and aligned with potential translational blocks.^7^ Secondly, all perspectives were considered equal in importance, allowing for thematic alignment. For example, all participant groups agreed that co-design was an important approach to improve research in post-stroke upper limb motor intervention development and overcome research translation blocks.^40^ Our approach allowed the identification of important considerations that were nominated by one group that may be deprioritised by another. It also allowed the identification of divergent subthematic emphasis between different participant groups. For example, clinical and lived experience groups had a stronger emphasis on the subtheme ‘therapist sway’ than preclinical or clinical research groups. Encouraging pluralistic voices in future research may promote co-design, minimise siloed work, and drive the development of new ideas.

This study has some limitations. Participants were asked to reflect upon ideal rehabilitation and not their actual experience and the generated themes need to be interpreted in this context. We acknowledge that there are other perspectives that warrant inclusion such as caregivers. Participants had wide-ranging yet complementary experiences in stroke recovery; however, the nature of our interview structure may not have allowed participants to share some information that may have expanded our derived themes. Participants were selected purposively, and some participants in the clinical experience group (*n* = 7) were affiliated with both research institutions and clinical institutions. Participants with lived experience were all in the chronic phase of recovery and our findings may not represent earlier experience of upper limb impairment after stroke. Although efforts were made to balance groups for gender and geographical location, our preclinical participant group contained mostly male participants (*n* = 8), and our lived experience group was mainly from Australia (*n* = 8).

Our thematic analysis highlights that effective upper limb behavioural interventions require the interaction of multiple intervention elements shaped by various contextual considerations. Developing such interventions will require careful consideration of the purpose of an intervention and presentation of each individual. Personalised complex behavioural interventions can be created by combining multiple interventions elements.

**Clinical messages**

- Post-stroke upper limb behavioural interventions are comprised of multiple intervention elements.
- Behavioural interventions can be personalised based on contextual considerations including the purpose of the intervention, the desired outcome of the intervention, and who the intervention is intended to benefit.
- Perception of what is a favourable post-stroke upper limb motor outcome varies depending on the participant group and the lens through which they examine recovery.

## Supplemental Material

sj-docx-1-cre-10.1177_02692155241265271 - Supplemental material for Considerations for developing complex post-stroke upper limb behavioural interventions: An international qualitative study Supplemental material, sj-docx-1-cre-10.1177_02692155241265271 for Considerations for developing complex post-stroke upper limb behavioural interventions: An international qualitative study by Matthew Wingfield, Gemma Hughes, Natalie A Fini, Amy Brodtmann, Gavin Williams and Kathryn S Hayward in Clinical Rehabilitation

sj-docx-2-cre-10.1177_02692155241265271 - Supplemental material for Considerations for developing complex post-stroke upper limb behavioural interventions: An international qualitative study Supplemental material, sj-docx-2-cre-10.1177_02692155241265271 for Considerations for developing complex post-stroke upper limb behavioural interventions: An international qualitative study by Matthew Wingfield, Gemma Hughes, Natalie A Fini, Amy Brodtmann, Gavin Williams and Kathryn S Hayward in Clinical Rehabilitation

sj-pdf-3-cre-10.1177_02692155241265271 - Supplemental material for Considerations for developing complex post-stroke upper limb behavioural interventions: An international qualitative study Supplemental material, sj-pdf-3-cre-10.1177_02692155241265271 for Considerations for developing complex post-stroke upper limb behavioural interventions: An international qualitative study by Matthew Wingfield, Gemma Hughes, Natalie A Fini, Amy Brodtmann, Gavin Williams and Kathryn S Hayward in Clinical Rehabilitation

sj-docx-4-cre-10.1177_02692155241265271 - Supplemental material for Considerations for developing complex post-stroke upper limb behavioural interventions: An international qualitative study Supplemental material, sj-docx-4-cre-10.1177_02692155241265271 for Considerations for developing complex post-stroke upper limb behavioural interventions: An international qualitative study by Matthew Wingfield, Gemma Hughes, Natalie A Fini, Amy Brodtmann, Gavin Williams and Kathryn S Hayward in Clinical Rehabilitation

sj-docx-5-cre-10.1177_02692155241265271 - Supplemental material for Considerations for developing complex post-stroke upper limb behavioural interventions: An international qualitative study Supplemental material, sj-docx-5-cre-10.1177_02692155241265271 for Considerations for developing complex post-stroke upper limb behavioural interventions: An international qualitative study by Matthew Wingfield, Gemma Hughes, Natalie A Fini, Amy Brodtmann, Gavin Williams and Kathryn S Hayward in Clinical Rehabilitation

sj-docx-6-cre-10.1177_02692155241265271 - Supplemental material for Considerations for developing complex post-stroke upper limb behavioural interventions: An international qualitative study Supplemental material, sj-docx-6-cre-10.1177_02692155241265271 for Considerations for developing complex post-stroke upper limb behavioural interventions: An international qualitative study by Matthew Wingfield, Gemma Hughes, Natalie A Fini, Amy Brodtmann, Gavin Williams and Kathryn S Hayward in Clinical Rehabilitation

sj-docx-7-cre-10.1177_02692155241265271 - Supplemental material for Considerations for developing complex post-stroke upper limb behavioural interventions: An international qualitative study Supplemental material, sj-docx-7-cre-10.1177_02692155241265271 for Considerations for developing complex post-stroke upper limb behavioural interventions: An international qualitative study by Matthew Wingfield, Gemma Hughes, Natalie A Fini, Amy Brodtmann, Gavin Williams and Kathryn S Hayward in Clinical Rehabilitation

sj-docx-8-cre-10.1177_02692155241265271 - Supplemental material for Considerations for developing complex post-stroke upper limb behavioural interventions: An international qualitative study Supplemental material, sj-docx-8-cre-10.1177_02692155241265271 for Considerations for developing complex post-stroke upper limb behavioural interventions: An international qualitative study by Matthew Wingfield, Gemma Hughes, Natalie A Fini, Amy Brodtmann, Gavin Williams and Kathryn S Hayward in Clinical Rehabilitation
