# Supplementary figures and images for: Considerations for developing complex post-stroke upper limb behavioural interventions: An international qualitative study
Source: Clin Rehabil. 2024 Jul 25;38(9):1249–63. doi: 10.1177/02692155241265271 (PMC11487871; doi:10.1177/02692155241265271)

Supplemental 1. Flow of participants through the study.

**
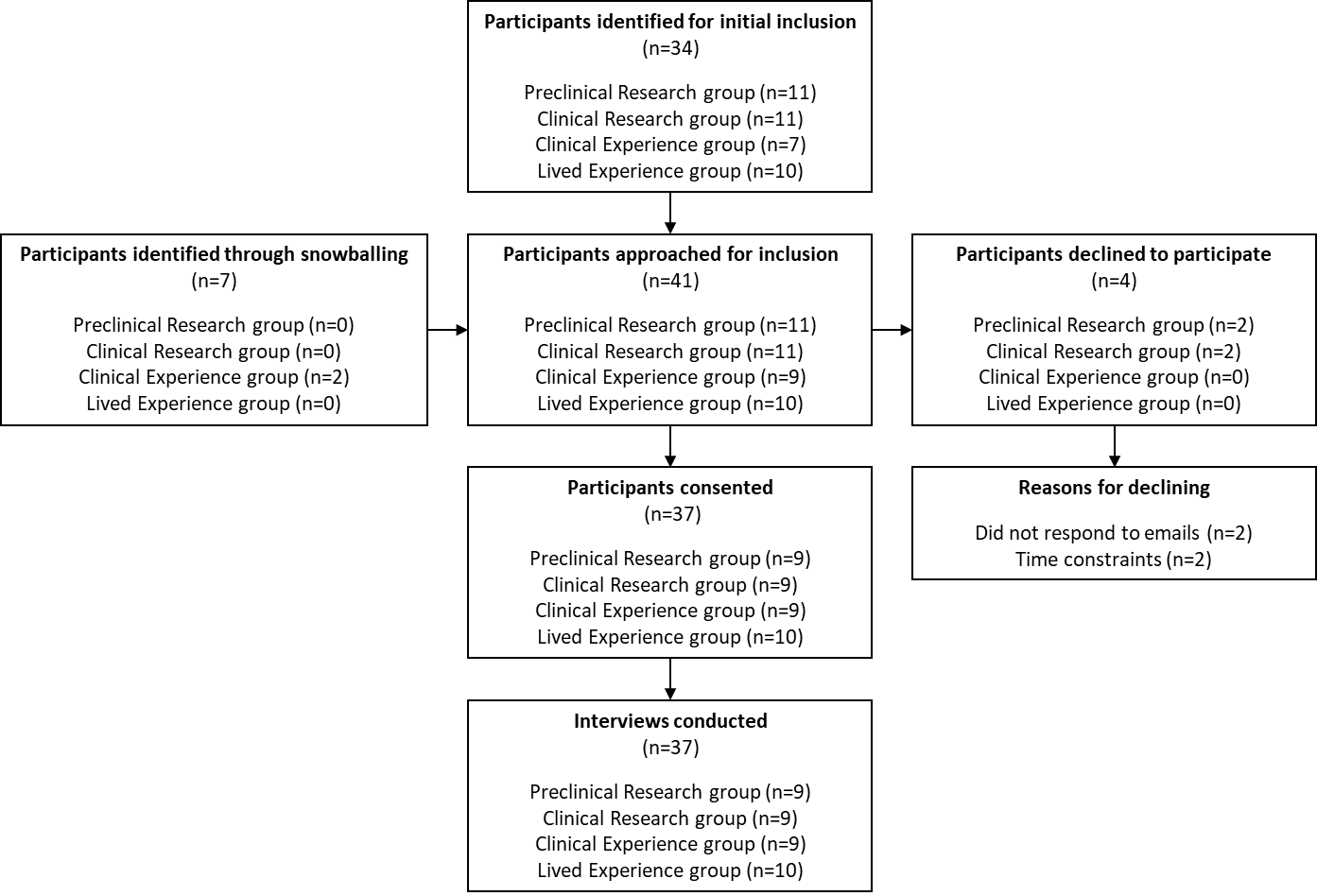
**

Supplement: sj-docx-1-cre-10.1177_02692155241265271 - Supplemental material for Considerations for developing complex post-stroke upper limb behavioural interventions: An international qualitative study [file sj-docx-1-cre-10.1177_02692155241265271.docx]
