# Supplementary material for: Considerations for developing complex post-stroke upper limb behavioural interventions: An international qualitative study
Source: Clin Rehabil. 2024 Jul 25;38(9):1249–63. doi: 10.1177/02692155241265271 (PMC11487871; doi:10.1177/02692155241265271)
Supplement: sj-docx-2-cre-10.1177_02692155241265271 - Supplemental material for Considerations for developing complex post-stroke upper limb behavioural interventions: An international qualitative study [file sj-docx-2-cre-10.1177_02692155241265271.docx]

# Interview Guide

## Agenda

1. **Welcome:**

Thank you [Name] for taking the time out of your day to participate in this research.

1. **Purpose**

The purpose of our interview today is to explore your perceptions around recovery of the arm and hand after stroke. This research is going to be used in my PhD program of research and will inform the development of an intervention that will be tested on people with moderate to severe upper limb impairment after stroke.

1. **Explanation of process for recording the interview**

The interviews will be recorded by the zoom platform’s recording system.

1. **Ethical Considerations**

We will do our best to maintain your confidentiality but under certain circumstances this may not be possible. There are no obligations to participate in this research and you can choose not to answer certain questions or to stop the interview altogether. All of this has been outlined in the PICF, have you got any questions any questions from reading that document?

1. **Introduction**

For context, I am speaking with [Name] as part of the [preclinical research/clinical research/clinical experience/lived experience] participant group to make sure we are on the same page can you state what your understanding of the research project is?

1. **Ground Rules**

- Please feel free to speak openly and respond positively or negatively to the questions, as you see fit.
- Please think about general principles rather than an individual example when responding.
- Please be ambitious and not restrained in your responses based on real or apparent practical implications.

1. **Questions**

**Q1) Today I would like to discuss how to maximise recovery in the affected arm and hand after stroke. What is your experience in this area?**

**Q2) What does good recovery of the affected arm and hand mean to you?**

**Q3) What would prove to you that recovery of the affected arm and hand has occurred?**

**Q4) What would you include in an intervention to maximise movement recovery of the affected arm and hand after stroke?**

**Q5) If you narrow your focus to people with severe arm and hand impairment. What would you include in an intervention to maximise THEIR recovery?**

**Q6) What affects recovery of the affected arm and hand after stroke?**

**Q7) Is there anything else that you would like to add that you don’t feel you have had the opportunity to say?**
