# Supplementary material for: Considerations for developing complex post-stroke upper limb behavioural interventions: An international qualitative study
Source: Clin Rehabil. 2024 Jul 25;38(9):1249–63. doi: 10.1177/02692155241265271 (PMC11487871; doi:10.1177/02692155241265271)
Supplement: sj-pdf-3-cre-10.1177_02692155241265271 - Supplemental material for Considerations for developing complex post-stroke upper limb behavioural interventions: An international qualitative study [file sj-pdf-3-cre-10.1177_02692155241265271.pdf]

- Roles in intervention development
  - Research question development
    - Clincial relevance
  - Translation of information
    - Assimilation
    - Communication across discipline
  - Operationalise interventions
    - Study design
    - Outcome selection
- Recovery
  - Definitions
    - Restitution
    - Compensation
    - Optimisation / Adaptation
  - Biomarkers
    - Presentation/ Diagnostics
    - Predictions / Prognositics
  - Measurement
    - Impairment
    - Activity
    - Participation
- Treatment
  - Complex interventions
    - Creating "buy in"
    - Dose
      - How much
      - Difficulty
    - Intervention Content
      - What is done
      - How it's done
      - Where it's done
    - Therapist delivering the intervention
      - Skills
      - Knowledge
