# Supplementary material for: Considerations for developing complex post-stroke upper limb behavioural interventions: An international qualitative study
Source: Clin Rehabil. 2024 Jul 25;38(9):1249–63. doi: 10.1177/02692155241265271 (PMC11487871; doi:10.1177/02692155241265271)
Supplement: sj-docx-4-cre-10.1177_02692155241265271 - Supplemental material for Considerations for developing complex post-stroke upper limb behavioural interventions: An international qualitative study [file sj-docx-4-cre-10.1177_02692155241265271.docx]

## **Theme 1. Clinical relevance should be the core of a “good” research question.**

Preclinical Research Group:

**Preclinical Research #1**

“The ideal role is that the pre-clinical researcher understands the clinical context of neuro rehab after stroke with a patient and seeks to model that in the design of their pre-clinical trials and testing paradigms when they discover new cellular and molecular mechanisms.”

**Preclinical Research #3**

“To translate findings related to stroke recovery and neuroplasticity to the clinical field. We're trying to understand how stroke recovery takes place.”

“These are studies that we cannot perform in patients. So, we need to do these experiments clinically in a lab. But they, do help us to understand why some patients recover any why some patients don't recover. That's a big question in the field of rehabilitation that hasn't been answered. With translational research, we might find answers that cannot be found with only clinical research.”

**Preclinical Research #6**

“I learned a very valuable lesson at that point: that it was really important to really pay attention and interact with clinicians, me being a basic scientist, as much as I possibly could because they gave me insights that I would never ever get from just reading research papers or textbooks about stroke and stroke recovery.”

“We need to get the preclinical people thinking more along the lines of doing things that will actually help the clinical people. As opposed to puttering around doing what they think themselves, might be helpful”

**Preclinical Research #7**

“Take advantage of the capacity to reduce the problem in order to make sense of its moving parts in ways that are really challenging to do in clinical populations. And also, to investigate things in invasive ways that you can't do in clinical populations while not forgetting that the models aren't the same as a clinical population.”

Clinical Research Group:

**Clinical Research #3**

“Getting their [stroke survivors] input into feasibility, interest, uptake, and kind of convenience so that they can actually undertake those programs.”

**Clinical Research #5**

“I think the role is to formulate good questions really…many of the studies that you see coming out are pragmatic studies and aspirational studies. That’s one thing. But there are studies that are just – it feels like they’re just convenient, they’re just things that particular groups are able to do so they do them, and actually whether it’s the right question or not seems to be a kind of secondary importance.”

“Yes, or no? If you can set things up as yes-no questions they just become easier to study, right?”

“See that informs your research question, right. So, the question is, if I’ve got some fine-grain detail about the brain and the pattern of damage, and I use, you know, machine learning approaches to – because it’s a complex dataset, right, it’s high-dimensional data - and I asked the question, does this add anything extra over and above what I can measure clinically, in terms of predicting outcome or predicting response to treatment, then that’s fine, but that’s the question. It’s not does a brain scan predict the future.”

**Clinical Research #8**

“I think people in my role have a responsibility to be really sensitive to what people who have experienced stroke want, and need. First, it needs to be patient centred. I think we have a responsibility to understanding what's possible in the real world. I know you just said to be ambitious - and we just can't fully ignore it. By the real world, I don't mean money. I mean people's energy, commitment, time, availability, resilience, perseverance, endurance - that's the psychology of engaging in any kind of treatment or therapy. That's what I mean by the real world.”

“I think we have a responsibility to be objective and evidence based in terms of the development of anything. We can't just - well, when you've got a hammer in your hand, everything looks like a nail. Right? You just have to be really clear eyed.”

Lived Experience Group:

**Lived Experience #1**

“I think [my role is] primarily to clarify expectations, limitations, and the plan which may or may not eventuate.”

**Lived Experience #3**

“I think it is important because obviously they are the people that have the firsthand experience and knowledge of what’s happening and can tell you not only the external signs of improvement, but whether they feel internally that things are getting better compared to the way they were before. So, I think in lots of fields, involving the people affected by it most is a very good way to go.”

**Lived Experience #4**

“There’s nothing like lived experience to help in research.”

**Lived Experience #5**

“I think I've experienced what therapies are effective and how much therapy is effective. So, I think lived experience is, I suppose it's the test. Whether it all works in theory is one thing but, the practical”.

**Lived Experience #8**

“Understanding is always enhanced through talking.”

**Lived Experience #9**

“I think it's probably fundamental for you guys to be able to begin with something like that. To identify just how much effort should be put on the arm and the hand compared to, say, the walking or something different to that.”

## **Sub-theme 1A. Breaking down silos: Forging interdisciplinary research teams.**

Preclinical Research Group:

**Preclinical Research #1**

“I do have that ability because I treat stroke recovery, I am a neuro rehab doc. So, I’m in the clinic with these patients. I see the kinds of deficits they have, the activity levels that they normally sustain in their community and home setting and all the process of subacute rehab after stroke and what is done in terms of therapy and current practices.”

**Preclinical Research #2**

“What does preclinical research mean? I think that usually means someone who is doing work in non-humans which I think is entirely false. You can do neuroscience in humans. Doing rigorous neuroscience in humans doesn’t make it clinical…you can do just as much rigorous neuroscience in the human as you can do in a monkey, or a rat, or a mouse.”

**Preclinical Research #3**

“it's translational, it doesn't get stuck in the lab as knowledge that is not being forwarded to the people who work with the stroke patients. So that's why I think it's important.”

**Preclinical Research #4**

“I would say that behavioural outcome after experimental stroke is complex and what we need is people who understand behaviour or rodents. This is a basic principle. And then of course we need experience in people who have experience in producing stroke and then evaluating the behavioural impairment and recovery.”

**Preclinical Research #5**

“We're kind of at the crossroad of neuroscience, engineering and clinical know how, if you will.”

**Preclinical Research #6**

“I don't think there's been really the uptake that there should have been. I mean, some people are obviously very aware of the preclinical research, but I think there are lots and lots of clinicians who really aren't, or they don't really know quite what to make of it.”

“Young basic scientists like graduate students, a lot of them have never even seen a stroke patient ever, never really saw what it was like to do rehabilitation and then they're sort of pretending to work on stroke recovery and they really have no idea what barriers are, or what the patients, and the constraints on the clinicians for doing the therapy. And by the same token, the clinical trainees, have no idea about the models that are used.”

“I think quite a few clinicians feel the animal work is really not relevant. And the basic scientists feel what they're doing really isn't research, they're just kind of floundering around and they don't really know anything about biology or anything. So, you know we don't talk anything more than a get together at scientific meetings… get people to break down those barriers and to really start interacting in a more meaningful way.”

“A lot of them have these built in biases about the other discipline that have grown over the years and it's very hard to change their minds. You see this at conferences all the time, where someone will make a sweeping statement about clinical research or basic science research and you know it's just nonsense.”

**Preclinical Research #9**

“I am in that translational bridge part of neuroscience to understand the changes or be descriptive about what changes take place in the nervous system after injury and what substrates can support the behaviour that we would like to recover. And then what particular interventions can we test in animal models in a controlled way with probably higher intensities and more invasive procedures than we can currently do in humans to try to get a handle on the most appropriate steps going forward in human research.”

“We make some very broad assumptions, some of them are good, some of them false.”

Clinical Research Group:

**Clinical Research #1**

“So, it’s not unusual to be pulled into discussions with my pre-clinical colleagues about what they’re doing.”

“I would say that’s probably fairly unique to the environment and it takes a little bit more effort on my part as a researcher to be conversant with them. It’s taken a couple of years for me to know what they’re saying when they’re talking about one thing and for them to know what I mean. So, I would say it’s not that common. It’s certainly not unique but it’s not that common and I think we need to see a lot more of it if we’re really going to make this pipeline work for solutions that are going to be helpful to human beings in the long run.”

“I think there’s a lot of nuances that happen in each spot along the pipeline that the people that work in those spaces know really well. You kind of get in your little space and you don’t really think about what came before or what comes after.”

“I just think that the ideas that we have need - the ideas that we have need to be vetted by people with broad perspectives as well as people with deep perspectives in each area along the way.”

**Clinical Research #2**

“I think our field tends to be a little bit - puts the blinders on, restricted in their thinking. But I think we’re going to have to get creative to get to the core of some of these things. There are fields where creativity is cherished and nourished. I’m not sure this is one of them, but it could be.”

**Clinical Research #3**

“We’re completely idealistic. I think it happens, but not always with the same kind of rigour of regularity that it should. So, it depends a little bit on what pre-clinical exposures you’ve had. So who are you working with, and what are you keen about, what are you aware of, what tools you have at your disposal.”

**Clinical Research #4**

“The field can move forward only if researchers truly integrate those two aspects of our understanding, of not only how the brain recovers and how the brain responds, but how the human as an individual responds and reacts to our interventions.”

**Clinical Research #5**

“I do see a lot of patients, and I think that has informed a lot of my thinking to be honest, which I think is the right way round, and I wish I’d got to be doing that, kind of involved in that sort of level of clinical activity earlier in my career.”

**Clinical Research #6**

“It’s even more important not only to understand the evidence but also the reasons why we are doing this and understanding the mechanisms that drive neurological recovery and behavioural recovery after stroke. Understanding the mechanisms is even more important than understanding the guidelines themselves about dosing or kind of therapy that you can apply.”

**Clinical Research #8**

“People in my role, have a responsibility to be really sensitive to what people who have experienced stroke want, and need. First, it needs to be patient centred. I think we have a responsibility to understand what's possible in the real world. Be ambitious - we just can't fully ignore it.”

“Preclinical work is essential to at least get a grip on just the basic biology of it - its potential and its limitations, and I think that's really informative. When I think of people who are working at this point in the pipeline, we’ve just this quite essential connection between preclinical work and good basic science…So, all that preclinical work is essential and you've got to be able to turn around and think about what that means for humans.”

**Clinical Research #9**

“I think it’s good to have a good scientific foundation for how we look at a problem, because, if you only look at things clinically, you can be misled and misled frequently. There are examples of that, certainly, in the literature.”

### **Subtheme 1B. Beyond the pipeline: Bench to bedside and back**

Preclinical Research Group:

**Preclinical Research #1**

“The barriers are understanding/knowledge of what’s happening in the clinical side. There are also barriers in the clinical side where the standards of practise are so sparce and varied that they are fully inadequate to engage the brain in recovery and quite frankly they are not worth modelling and attempting to model in the research laboratory.”

**Preclinical Research #2**

“The goal that we have with our preclinical research is to translate findings related to stroke recovery and neuroplasticity to the clinical field. We're trying to understand how stroke recovery takes place, what the underpinnings are at the level of neural mechanisms.”

**Preclinical Research #6**

“I think the preclinical research brings in a lot of guidelines about: dose, timing. That I think need to be explored in clinical research.”

“I mean ideally, we would always be working, probably with middle aged rodents who had maybe some hypertension, diabetes, and things like that.”

**Preclinical Research #7**

“When the basic science is pointing to a potential therapeutic direction, it is great idea to take advantage of animal models to get proof of principle and the potential for a treatment to work. So, you need to remember how you reduce your problem right? Because the translation needs to consider that the population that is going into has a lot of heterogeneity that wasn't in your model system.”

**Preclinical Research #9**

“The non-human primate models are highly appropriate. And as we have the rodent models that really predominate and are very useful. I think they've been extremely helpful at elucidating some of the basic molecular mechanisms of plasticity and there is a reasonable analogy, to the types of recovery that occur in non-human primates and humans. But the sensory motor structure is substantially different in certain respects, so you can translate a lot more from the non-human primate models than from the rodent models.”

“We don't necessarily replicate the human condition in our animal models in terms of the anatomy of a stroke. The kinds of tasks are analogous but certainly the stroke anatomy is very different, just because of the models. But that's where we start to depart from answering the clinical questions directly where we try to address a potential mechanism and test the hypothesis that particular mechanistic endpoint.”

Clinical Research Group:

**Clinical Research #2**

“One of my roles at least for me is translation, finding things that work the best in animals and try them in humans for the first time…how can we go from bedside impression to an informed decision based on assessments of brain structure, function, genetics, whatever it might need.”

**Clinical Research #3**

“So, I think it’s a critical bridge. So, you have your kind of data coming out of that pre-clinical trials, and then the practicalities of the clinic, which those two often are not too well aligned. So then our role would be to try to sort out a way to adapt the pre-clinical data to the patient, the human patient condition, that’s realistic and thoughtful. Also taking into consideration the really big differences in terms of response in the complexity of the brain that affects how much we can adapt directly from pre-clinical models, specifically animal models of course…the true clinical, the clinicians comes back to us as well. So you kind of have this multi-directional flow of information.”

**Clinical Research #4**

“I saw my role as primarily integrating neuroscience into practice. So, my interventions were informed primarily by the neuroscience, but as I grew into it and began to appreciate the importance of psychological science to recovery - after all, we're dealing with human beings who have had this major event in their lives, it's completely disrupted their life and their role in whatever they do with respect to their life. I began to realise that there is likely an interaction between neuroscience and psychological science…I've always had my eye on how we can use the science to inform our practice and improve recovery.”

**Clinical Research #5**

“I find it inspirational, and I think it allows us to talk about things like spontaneous biological recovery and wonder whether that is a real thing in humans, it probably is, but how does that impact on the way we actually treat patients? Is it a useful thing to think about? But in terms of a research study, I’m not sure, be as clinically relevant as possible in these things but they’re still rats, or mice, and their nervous systems are not exactly the same. So, I think we can inspiration and ideas from there.”

“I am interested in the idea of finding expert patients who can genuinely reflect things back to us that we haven’t thought about.”

**Clinical Research #6**

“What can we learn from human studies and what are the knowledge lacunes to solve in humans and what can be solved maybe, in animal studies? So, what is needed? And so, a translational point of view you need. But also, the other way around what they found, what’s the meaning for humans?”

**Clinical Research #7**

“I bring the neuroscience and the clinical problems to the engineers and work with engineers in developing technologies.”

Clinical Experience Group:

**Clinical Experience #1**

“The useability of whatever you're presenting. I think how realistic it is.”

**Clinical Experience #3**

“We are looking and seeing what is actually walking through the door. Which I think, is a really important thing to keep in mind when we are developing an intervention to trial. Not only from the point of view of it being appropriate but also generalizable to the population that we see. I also think we bring the nuanced idea of: you've got your ideal world, and your ideal intervention, but then there's also the reality of implementing it, and the feasibility of implementing it in a world that's not ideal. I suppose, outside of a research trial”

**Clinical Experience #6**

“Because I am an occupational therapist also taking a look at how those intervention really support their ability to return to functional use of the arm.”

**Clinical Experience #8**

“It's really important that anybody that's developing an intervention is liaising with clinicians and I guess seeing what the current state is and seeing what they might feel is lacking, but also seeing whether what they're trying to develop is pertinent and relevant to - this is predominantly stroke I guess you're talking about, so stroke survivors as a group. I guess a clinician's role is about the relevance and about the practicality.”
