# Supplementary material for: Considerations for developing complex post-stroke upper limb behavioural interventions: An international qualitative study
Source: Clin Rehabil. 2024 Jul 25;38(9):1249–63. doi: 10.1177/02692155241265271 (PMC11487871; doi:10.1177/02692155241265271)
Supplement: sj-docx-5-cre-10.1177_02692155241265271 - Supplemental material for Considerations for developing complex post-stroke upper limb behavioural interventions: An international qualitative study [file sj-docx-5-cre-10.1177_02692155241265271.docx]

## **Theme 2. Balance restitution and compensation to maximise outcomes.**

Preclinical Research Group

**Preclinical Research #1**

“Those patients that regain some meaningful use of their affected function after stroke whether that is language, motor, sensory, gait, balance, some aspect of that.”

**Preclinical Research #2:**

“Well, let’s first define what recovery would mean. And recovery would mean: some return towards normal motor control.”

**Preclinical Research #3**

“True recovery is the return of the actual function that is lost as a result of a stroke. Compensation, of course, is a patient able to perform a task in a similar way, but executed differently using alternative strategies. Like from the patient's perspective recovery could be either true recovery restoration of function - let's call it that way - or compensation. And the patient could say I feel recovered because I’m now able to open the refrigerator again which wasn't possible in the first weeks after stroke.”

“That's not how we see recovery, although it is recovery from the perspective of the patient. We're trying to see if we can get towards restoration of function, so that true recovery, because we think if that's achievable you can even further improve the recovery potential of patients. So, for me recovery is both true recovery and compensation, but I think if you can get to the true recovery, the recovery can get to a higher level.”

**Preclinical Research #4**

“I would be happy to see treatment effect, I would be even more happy if i could say that it is from true recovery.”

**Preclinical Research #5**

“Therapists always say the word functional and that means, in essence, you need to have pretty good hand control. I look at it a bit broader I'm already happy if people can reach the objects in their workspace that they may not be able to do. Even by lifting up the weight of their own limb.”

**Preclinical Research #6**

“As much as possible normalization of synergies to, a kind of, pre stroke level. I think that is really what I would consider to be a good recovery. And you know, that’s going to vary depending on the severity of the of the stroke and where it is, and what other factors...but I think, then the normalization of behaviour as opposed to regaining function through almost entirely compensation.”

“We know in people that there are many, many ways to accomplish a task like reaching for a cup. And all kinds of compensatory movements can get built in. Like shoulder rotations, you know, leaning forward at the trunk and other things. And then the extreme is just using the unimpaired limb. Animals do almost identical sorts of things. So, they accomplish the task fairly well at the end, but they do it in a very inefficient manner. So, I think trying to reduce the abnormal synergies and make them more like what they were prior to the stroke through the right type of intervention...and you know...the right type of training. This is the way that we should try to go at least up to a point, and then it may be at some point you just say well, “This patient, this animal doesn't have the capability of moving beyond this way.” And then maybe it would be useful to just work on compensation, but I think trying to achieve restitution initially is really what one should try for.”

**Preclinical Research #7**

“It depends on the stroke, right? But let's say optimal, and we don't know anything about the stroke, recovering fully normal ways of using the arm and hand to the capacity that you had before this stroke would be optimal recovery.”

“If you're using say, your contralesional hemisphere more for moving the impaired arm versus more of your ipsilesional hemisphere and you have exactly the same injury and you're equally good behaviourally - then it doesn't matter.”

**Preclinical Research #8**

“The patient that has that luxury, that they have enough residual material to use the previously functional network, then yeah, of course, the best solution - this is what evolution has done for millions of years. So of course, that’s - if you’re able to go back to that, that’s the best solution. I think that the big question is - if that does not exist, then what is the next best thing?”

“Biologically, why would you care that you do it the same way after? I mean, it’s not the same brain. So maybe the best solution with that new brain you have - like the residual brain - is not the same solution as the one before and if you’re just trying to do the way you did it before, you will never be able to and you’re wasting your time and this is a bad strategy.”

**Preclinical Research #9**

“A good recovery would be a functional system. I think that there are differences between clinical endpoints which, by their nature they tend to be more, on the spectrum from impairment to disability, more on the disability side. Good recovery I'd really try to take the clinicians view that if I see a patient walk into the office and they appear normal, whereas before they had an obvious disability, Ultimately that's good recovery.”

Clinical Research Group:

**Clinical Research #1**

“To be completely honest they [terms of compensation and restitution] irritate the hell out of me [laughs]. The reason that they do, the reason that they do is because people put them in boxes, right? They’re like, you either do this or you do that but I don’t think that’s true. I think you actually do both and it’s a long continuum with a lot of grey and very little black and white.”

“a kinematic analysis of a reaching and grasping task prior to the intervention and then after. What we see in the data is that some people restored some features of the - or you know, some kinematic variables became more normal and other kinematic variables in the same or in a different individual became less normal. So again, it’s not like what you’re seeing is one box or the other.”

“I’m thinking of recovery of the neurons, then there is no good recovery. Those neurons are dead. If I’m thinking of recovery of networks, maybe I have recovery of the network, depending on how much is damaged. If I’m thinking about recovery of impairments, that’s one thing. If I’m thinking about recovery of their capacity for activity or if I’m thinking about recovery of their actual activity in everyday life, which we call performance, or even recovery of the role - participation roles in life. So each of those things to me has their own trajectory and their own - they don’t all go together necessarily.”

“I think that good recovery would be a resolution of the paresis, the somatosensory deficits, the hemi spatial neglect. All on up, depending on I guess if we’re just talking about the arm, potentially resolution of apraxia if that’s present.”

“So I think what irritates me is this - people talk about compensation as if it’s a dirty word and we can’t allow it to happen when that’s the way - it’s - at the brain level of the system, that’s exactly what’s already happening no matter what. So it - and it’s also happening at various other levels of the system and that’s what helps people get to be able to do the things they want to be able to do.”

**Clinical Research #2**

“Usually, we try and use the term to mean some kind of true return of the original behaviour. So that's restitution. If you look at the functional level, compensation can be considered a form of recovery. There are times when that needs to be part of the conversation, but I think a lot of us in the field aim for what we might call true recovery. What does it mean? It means getting back some or all of what was lost.”

“Supposing the person is able to do a very specific motor task, a reaching task, that by all kinematics and behavioural assessments looks relatively normal but the brain is working triple overtime to produce that bedside phenotype. Is that true recovery?”

“What I would aim for, what I think the field should aim for - we should see how close we can come to getting return to original movement, original behaviour.”

**Clinical Research #3**

“There are inevitably those more minimally affected individuals who we can get a true restitution of arm recovery. Then as we – I think progressed on severity, both of the deficits and of the injury of the brain, I think our goals have to shift to enable maximal function. So, in some of those people it’s going to be some combination of restitution and compensation, and in some people, it’s going to be full-out compensation, because they literally don’t have enough brain left for restitution to take place.”

**Clinical Research #4**

“Recovery that I'm well aware of is that the same sort of movement strategy that was used before the stroke is used when one is fully recovered. Now what that means - that probably means at the neuromuscular level we have the same coordinated muscle firing patterns to produce certain kinds of movements. So you could say fast movements, slow movements. So that's kind of the field has sort of taken that as a definition of recovery.”

“The challenge is to get people out of those boundary conditions and get them to optimise their recovery. Maybe what that means is - and I've been doing a lot of thinking about this lately - manifesting their full capacity, whatever that capacity is, to its highest degree.”

**Clinical Research #6**

“Knowing there is a neurological deficit, not all things can be repaired. There is always compensation. This compensation part, you may also call it adaptation or in the cognitive fields we call it a “coping behaviour”. To understand these adaptations, that’s one of the most poorly understood parts in rehab medicine. Also, saying indirectly, that normal behaviour is not a good reference to understand abnormal behaviour after stroke.”

“Patients accomplish their task in the most optimal way at that moment. So, optimising that performance with the degrees of freedom that they have in order to accomplish a certain task like grasping or whatever.”

“We call it brain plasticity – that these networks are adapting, if there are some deficits somewhere in these networks. These adapting systems which can only be explained by mechanisms: some people say unmasking, let’s call it neuroplasticity, whatever. There is brain plasticity in adult brains but it’s not by growing axons and dendrites in the brain from one side to the other side. It might be in babies and non-adult animals or in primitive animals. But in monkeys and humans it’s much more subtle, and this synaptogenesis, that is happening, more at the dendritic level and subtle in the sense of making new connection with other existing neurons that are still functioning. These small subtle structural plasticity changes that are possible in the brain isn’t able to make full repair of what is lost but a stroke in these networks.”

“Neural restitution is not complete, so the behaviour restitution is not complete. We have neural substitution happening in the brain and so ultimately for networks optimising what happening: we can learn but it brings always optimisation and adaptation with it… Two parts: neural substitution and behavioural restitution, and behavioural compensation and adaptation.”

**Clinical Research #7**

“If we think of recovery of restitution, so true recovery as opposed to recovery of function, okay? So if we're thinking of the restitution then we're aiming to enable a patient to perform a task in the same way as they would have done before their stroke; so with the same biomechanics, not using compensatory movements. That doesn't mean to say that from a neurological point of view, they will be using the same pathways. They almost certainly will be using newly formed pathways or using strengthening of existing pathways. So, for instance, somebody with a very severe stroke may actually get recovery and when you look at their functional MRI, they might actually be using their unaffected hemisphere in a very extreme case. So I think when we're talking about restitution recovery, we're not talking about the brain becoming as it was before the stroke. We're talking about the actual performance of the movement being the same as it was before the stroke.”

“Recovery is not recovery of the brain, going back to as it was before the stroke. I think that that is - I think that's a slightly ridiculous idea because in fact the brain is plastic anyway, so the brain is changing all the time. I think the important thing is that the way the person performs the movement - they're able to perform the movement as they would - as they did before with the same movement patterns, the same muscle groups, the same synergies and so on.”

**Clinical Research #8**

“A good recovery, to my mind - this is for the upper limb - is one where people reach their maximum biological potential.”

“Reaching your maximum biological potential, let's just say that you do the best you can with the sensory motor system you've got left, and you then adapt to and compensate for the residual impairments using all your other biological potential such as strategy, planning, memory, orientation, good execution, good cognitive function, good communication. So, that's still all of your biological potential.”

“it's your whole brain, your whole system. Because stroke is, by definition, a permanent neurological deficit. The extent to which you need to adapt for or compensate to that really depends on the severity of that deficit. But it doesn't matter whether you're talking true recovery - so proper restitution, doing the thing the way that you've always done it, using exactly the same bits of your brain that you always used before - or adaptation, or compensation. It doesn't matter. Your biology is a constraining factor on that.”

“A good recovery is when somebody is maximally functional, independent, their disability is minimised, and that's going to be through a compensation - sorry, through a combination potentially of all sorts of things - recovery, adaptation, compensation. But if they're maximally functional their disability is as small as we can make it and they're able to participate, that's a good recovery.”

Clinical Experience Group:

**Clinical Experience #1**

“I think the patient would say restoration, but for me as a clinician knowing that restoration isn't always possible, compensation is just as good in those cases. I think acutely we try for restoration, we try for recovery in whatever way that means, as much as can get, but at the end of the day if that's not happening then compensation is necessary.”

**Clinical Experience #4**

“Good recovery means a lot of restitution or recovery of lost function. With some of the stroke upper limbs, over the process of time plus therapy, you will see a return of a lot of impairments, and functions and skills. So, I think there's capacity of some of those people to really normalize their movements.”

“Most of the recovery we see is degrees of compensation. So, where I’m bringing in other movement strategies, I’m making movements perhaps a little bit more simple and less complicated in terms of inter joint movements. And shoulder plus fine finger movements. I'm probably saying, ‘What is the best compensation that we can make?’ And I think that drives a lot of recovery in the majority of patients.”

“That term compensation gets thrown around a lot, so a lot of people have different ideas of what compensation means. But in my head, it's just degrees of compensation.”

**Clinical Experience #5**

“Essentially get back to essentially everything they did previously. Then good recovery would be full recovery. So, full recovery of reach and grasp, manipulation, fine motor, gross motor, everything. And we see in some people that it is entirely possible that they get something which very much approximates return to full function. But I think for other people where that isn’t achievable, or isn’t achieved.”

“The overall thing is compensatory, but you've actually improved power through range, so that they can actually get to where it needs to go to stabilize those things.”

**Clinical Experience #6**

“Essentially what that means is it may still be an arm with deficits, with some weakness, some increased tone and stuff like that. But for as long as you can use that arm in most of the task that you wish you can resume, then I think that is good recovery.”

**Clinical Experience #7**

“You come out from Uni and you're really optimistic and you think that you can actually make everything perfect again. Then you realise if you repair a smashed car, it will never be the same. So I'm not saying that we lower the bar. But sometimes I think we know that someone might be a little bit clumsy with their grip, but at least they can get back to work and it takes a bit longer and then they can do most of their activities. That's a fantastic outcome.”

**Clinical Experience #8**

“Good recovery is better not perfect. You know, better than what this person started off with. I think that’s the unfortunate piece that we have to always address with patients is better doesn’t mean back to normal and I think that’s often the hope for patients or maybe an easy long-term goal but I think that’s unrealistic to expect that every patient is going to return to their normal function after having an injury to their brain”

“It’s like splitting hairs but the difference between living with the effects of stroke to the best of someone’s ability versus recovery being to me, reversing the effects of stroke. In some cases, you might see someone just spontaneously recover back to almost normal behaviour, right? Normal abilities. Then there’s other people who will never - their arm and their legs will never be the same but their life will be improved by means of these changes and the way they are able to turn on or activate or control their arm within this altered movement pattern.”

Lived Experience Group:

**Lived Experience #1**

“Same as the left hand! What it was or could do before I had it. But I know, I don’t think that they can undo that process, what do you call it? But you never know.”

“What can we fix? I would love it to be fixed but like I said I don’t think so 100% it will be.”

**Lived Experience #2**

“In the end, maybe it doesn’t matter how you do it, but I can’t really believe that that’s ideal. I mean, how you do it – I can do lots of things now if I just forgo using that hand and try to engage the alternate one. So, compensating without actually using it is the way to get some things done. That’s not very satisfactory, but maybe I have to accept it.”

**Lived Experience #3**

“Ideally it would be to have normal function back in my left hand and left arm. I suppose initially my hand was fairly crunched up in a little bundle, which is not good. Fortunately, that relaxed over time, so I don’t have any problems with that as I now know a lot of people have.”

**Lived Experience #5**

“It's a difficult thing coming to terms with living adaptively as a survivor. But I think that is part of the grieving process as well. I am warming to the idea of having to do things adaptively.”

**Lived Experience #8**

“Regain about 80% of the use of it.”

“I know that it can move. I can move it, but I can't use it.”

**Lived Experience #9**

“I would prefer just to be able to get a bit more usage out of it so I can become a little bit more independent. But long term yeah, I mean, definitely looking for options to get the hand in the sense of the sense of the palm, you know, better and better. As I get to a certain level I’m not going to stop just because I can, you know, grab, and I can do certain things, and I’ve become independent.”

**Lived Experience #10**

“I would like it to be better, that's it. Yeah. I know I can't get all the way better, but yeah, I want to strive for 100 per cent.”

“I want to use it like before. So, I know if it's like I can use it, sort of, that's good, but still, I want it to be like before.”

### **Subtheme 2A Good outcome: going beyond an outcome measure.**

Clinical Research Group:

**Clinical Research #1**

“If I’m a knitter and I want to knit a stocking for my grandson, I can still knit but if I’m not a knitter, I don’t have to return to knitting. To participate in those life roles that are important to you. Again, whatever those may be.”

“They’re not recovered…they’re six points better on the scale and that’s great, but they’re still moderately affected and there’s still a tonne of things they can’t do.”

**Clinical Research #2**

“People want to get back to their behaviour. They want to be able to make coffee, hug their grandkids, drive a car. If all the guy wants to do is turn his TV on and so he bites the switch that turns the TV – and he achieves functional status, then power to him.”

**Clinical Research #3**

“Firstly, I’d like to know if the patient considers it good recovery – that would be my first thing. It’s a little bit frustrating because grant agencies don’t really like that answer, but I think that that’s probably the best answer – are they satisfied with their recovery? For me, best recovery is that it’s something that’s functional in daily life. So that you have minimised the impact of the stroke on this individual’s ability to do whatever it is they’re interested in and is important to them.

That’s going to be massively variable from person to person. I don’t know if it’s being able to pick up their grandkid with two hands, swing a golf club, or maybe they are more interested in just having the stroke-affected arm be something that they can use to stabilise and do most of the other tasks of daily life with their non-stroke arm. So I just think it’s a massively variable outcome”

“For me, best recovery is that it’s something that’s functional in daily life. So that you have minimised the impact of the stroke on this individual’s ability to do whatever it is they’re interested in and is important to them.”

**Clinical Research #5**

“It’s pretty simple really; somebody just needs to be able to use it functionally for whatever it is that they want to do.”

**Clinical Research #7**

“What we're trying to do is to work with our patients in order to help them to achieve their own goals. How they achieve it - how they achieve them, I think we shouldn't be fixated about.”

Clinical Experience Group:

**Clinical Experience #3**

“I probably embed that conversation in something around their function and what their end goal is.”

“I think if I was a patient with a really severe stroke and I someone told me there’s a 5% chance that I will recover my arm I would be like “I'm not going to spend, you know, hours and hours and hours of time, you know, taking heaps to pills or buying this funky device to get my arm back. If you’re telling me I’m only going to get 5% recovery?” I'd be like, “great. you know, write that off, buy an awesome sling, support my shoulder and move on with my life. All the other areas of my impairment.”

**Clinical Experience #4**

“So, I think good recovery is something where an arm is being used where a patient has a level of satisfaction.”

**Clinical Experience #5**

“It has to be taken into the rest of the recovery from stroke for the other person as well. for example, if you have someone who's had to have said, a relatively major stroke where they've had to redefine their life quite a lot. Then what recovery means for that arm and hand is in the context of overall recovery. But for someone else who has had, otherwise, a generally quite a good return of their other functions. Or their other functions weren’t as severely affected and their aim is to essentially get back to essentially everything they did previously.”

**Clinical Experience #6**

“I think what it looks like is for you to be able to use that arm in a majority of the functional tasks that you would like to perform in your daily life.”

**Clinical Experience #7**

“I like to see them achieving something that's functional and meaningful for the person. I like to see them having success.”

**Clinical Experience #8**

“I guess it's more important what it means to the individual that's affected for me. I might consider somebody to have good recovery but they might not consider they do.”

Lived Experience Group:

**Lived Experience #2**

“My initial goals were to be able to drive, be able to write, and be able to use the keyboard. Well, it’s two years down the line and I can’t do any of those things. I think they’re further out of my range of expectation now, certainly than they were at that time. Now I’d just be happy to be able to get my arm up on the table for any moment, to sit at the dinner table and be able to do anything.”

**Lived Experience #3**

“Things like even picking up grandchildren. Having sort of a normal relationship with grandchildren, I think, would’ve been something I – it’s something I miss…Minor things like doing up shirts and things like that. Trying to do up buttons on both sides.”

“You know that’s the case that everyone is different”

**Lived Experience #5**

“For me, personally, my big picture would be to be able to ride a bike again because I was an avid cyclist before my stroke. So, to be able to hold handle bars and ride and have shoulder movement. That sort of thing. That would be good recovery. Set a goal and achieve it.”

**Lived Experience #6**

“Getting dressed, answering the phone, it just really makes, I find – so, I can get away with my leg, but for some reason I’m really missing my arm.”

“By not having an arm. I can’t hold my kids. My youngest is 29, he suffers from anxiety. So many times, I want to hug him. I can only hug him with one arm. It’s not the same. This is deep and meaningful here. I just want to – I tell him all the time, there’ll come a day, you’ll see, I’ll hug you. That’s very important to me. You can imagine?”

**Lived Experience #7**

“I can't dress myself. And being the female, that's even harder. Just being able to do my hair, dry myself. All the simple things in life.”

“I've got great grandkids now. I can't even f***ing pick them up. That’s what I need!”

**Lived Experience #9**

“Can do certain things that I’ve learned. You know, I start to use my own arm a little bit more.”

### **Subtheme 2B. Recovery is a puzzle: measure all pieces.**

Preclinical Research Group:

**Preclinical Research #1**

“Clinically and preclinically I think we need to move to kinematics. I think we can’t rely on some of the traditional outcome measure in both the human and in – most cases the preclinical animal is the rat or the mouse. I think we’ve gotten pegged to well established outcome measures that are confusing recovery and compensation. Clinically we have a better handle on disassociating those most of the time. But ground proof lies in kinematics.”

**Preclinical Research #2**

“We believe in that kinematics and kinetics and better measurements.”

“Fugl Meyer and the ARAT have reasons, they’re not terrible. It’s just that they were never meant to be cast in stone forever. In other words it’s just that, they are a reason not to take a closer look at the arm and hand. Right? Let’s be pragmatic,”

**Preclinical Research #3**

“How we are trying to differentiate between true recovery and compensation strategies - looking at these kinematic changes over time… it's very tricky and you need to understand very well what you're looking for.”

**Preclinical Research #4**

“Slow motion video recording to see exactly the movements, different phases. Then it is possible to compare normal behaviour and impairment behaviour in a very detailed manner. So it is possible but its very laborious.”

**Preclinical Research #5**

“So those will be way more quantitative metrics that we currently do with our Fugl Meyer and other approaches. Which are a nice first approximations, but I can have 10 people with the same Fugl Meyer score that I can pick apart completely with these more quantitative metrics. So, we're using quantitative metrics that look at reaching distance as a function of shoulder abduction loading, hand opening as a functional shoulder abduction loading, involuntary grasp as a function of abduction loading and all of these metrics we should be able to be able to take in a in a short amount of time.”

**Preclinical Research #6**

“Then I think what's really valuable, which a number of investigators are starting to do more and more of and include, are quantitative kinematics where you can really look very closely at the movement patterns. Now, in our case on the preclinical side, we have the advantage of: We can do pre post easily. Because we have control, you can't do that exactly. But you can have a normative population of age match controls and look at the kinematics and reach to grasp or some other task like that and see how it's being performed. Then you can use that to, sort of, really help you decide whether am I getting some restitution in here, or is it really just mostly compensation.”

”I think the Fugl Meyer seems to be the best test for measuring impairment. And you can get changes in impairment, as opposed to, Modified Rankin Score and some of these other things that are pretty subjective and insensitive. Then I think what's really valuable, which a number of investigators are starting to do more and more of and include, are quantitative kinematics where you can really look very closely at the movement patterns. Now, in our case on the preclinical side, we have the advantage of: We can do pre post easily. Because we have control, you can't do that exactly. But you can have a normative population of age match controls and look at the kinematics and reach to grasp or some other task like that and see how it's being performed.”

**Preclinical Research #7**

“In the animals were actually behind the clinical side in differentiating between compensation to recovery. Because kinematics are challenging! There's actually a lot of movement on the animal side in this. And using machine learning approaches…I'm not doing that! I'm hoping someone else figures it out for you. But the bigger issue is that a lot of the animal side doesn't really care. I talked to them about compensation versus recovery and their eyes start to glaze over.”

“Our models of upper limb impairment, we do have measures of movement abnormalities as well as performance capacity and that's how we distinguish between the two.”

**Preclinical Research #8**

“I think that we need to have kinematics or a muscle pattern. I think muscle pattern would be even more precise because then it’s not just like the movement of the limb but effectively how you’re able to manage that specific movement. But I guess that kinematics is good enough and that’s why I think - a lot of people are moving to that.”

Clinical Research Group:

**Clinical Research #1**

“Level of the activity restriction is that they would perform very well on all the standardised tests but they would also be able to engage their upper limb in activities throughout their day.”

“Then I guess participation would be a good recovery, would be able to continue to participate in those life roles that are important to you.”

“Certainly, the field uses the Fugl-Meyer as a measure of - global measure of impairment. You could have a whole conversation about - debates about the value of that measure or not. We use it a lot in research. It’s not that useful in Clinical Experience for making decisions about your patients, in my opinion. So, I always try and avoid it but I have - certainly have studies that we’ve used it in. Raw things like grip strength. That would give you an indicator of the level of paresis. I’ve used monofilaments before. We so rarely want a pure impairment level measure of somatosensation that perspective often isn’t worth applying. Yeah, I mean I would use things - I think that a lot of those standardised upper limb function tests, the Action Research Arm Test, the Wolf Motor Function Test.”

**Clinical Research #3**

“As we get a little bit more sophisticated hopefully with those measures, maybe better measures of arm use and arm movement. We’re playing with some approaches right now to use videos to characterise arm movement in the real world, and trying then to extrapolate kinematics from those data, from those videos, in order to look at how that arm is truly being changed in the real world. I think those types of measures are going to be really valuable as we get better at collecting them.”

“I do lots of multi-modal stuff. So I really think you’ve got to look at changes in structure and function together – they really go hand in glove.”

“There have been these recent studies showing that patient perception and our indices don’t always track together. So I think certainly patient perception is critical, and I would start there. It’s fine to look at an objective outcome measure, so that’s nice as well. Something like the ARAT for example. But it’s also really nice if you can see changes in activities of daily life in the real world, and so wearable sensors are really, I think, crucial there. So, looking at changes in accelerometry, or as we get a little bit more sophisticated hopefully with those measures, maybe better measures of arm use and arm movement. We’re playing with some approaches right now to use videos to characterise arm movement in the real world, and trying then to extrapolate kinematics from those data, from those videos, in order to look at how that arm is truly being changed in the real world.”

“Changes in impairment, function, and I think participation is really critical, and I think a lot of times we don’t have very good tools for measuring that and so we don’t do as good a job with that particular outcome measure as we ought to.”

**Clinical Research #4**

“If you're using the ICF to kind of lay out your levels of disability, the Fugl-Meyer in stroke is probably one that captures the impairment level. It's probably not a great measure, but it's what we have. We have it across numbers of different studies. Activity would be something like the ARAT. Something where there is not only a time score, but a movement strategy - some information about how the task was performed. We don't have a lot of those, you know. We have movement time, which turns out to be a horrible, I mean, it's a - it's quite superficial and it captures something, but it's certainly not quality and it's not skill.

Which I believe the activity level should capture is skill. We want to capture efficiency. So that's another sort of thing probably at the activity level. At the participation level, we're pretty much stuck with patient reported outcomes, which I think are underutilised. You're getting at one of my pet peeves here, but I think most studies, whether they're basic mechanistic or qualitative, need to include both kinds of measures as a kind of an internal verification or validity check.”

**Clinical Research #5**

“Have things that cover impairment activity, participation, patient reported outcome. So, we’re covering all our bases there. So, yeah, you’re right, the impairment measure we have is the Fugl-Meyer, the ARAT has a bit of impairment and a bit of activity, so it’s a little bit mixed, and then we have the CAHI for again, more activity. Then we’ve got other stroke impact scales.”

“Recommendations from the SRRR about using kinematics in upper limb recovery are of interest,”

“what you need to measure to demonstrate recovery, well it depends what you’re trying to do, and if we’re talking about in the context of clinical trials it really depends on what you think the level at which your intervention is operating. So, if you’re going to do brainstem or you’re going to give a drug, you have to think that that’s going to have an impact at the level of impairment so you must have an impairment measure. But of course, people design trials to - they just say, oh well we’re going to give people fluoxetine and we’re going to look at the modified rankin. It makes no sense at all because there are so many things in between what the drug might be doing and what needs to happen for the modified rankin to change. The effect of the drug is just being diluted.”

“if you’re doing a self-management strategy, you’re not going to have an impairment outcome. So, it depends, yeah, what level of disability or of the ICF, more explicitly, you think you’re targeting, then you need to have an outcome measure which is proximal to the proposed mechanism of action of your intervention.”

**Clinical Research #6**

“The only way to tackle the problem of restitution is not by clinical measurements for outcome, but rather biomechanics. So, what we do is look in a standardised way at the biomechanics on the outside. If you like to understand the outside, then you need to also understand the inside. You need to connect the inside: brain activity, with the outside: biomechanics. So, this neural restitution/neural repair should be connected longitudinally in time if we would like to understand recovery with the long-term change of the biomechanics: what is seen on the outside in terms of quality of movement. This is the big connection.”

**Clinical Research #8**

“We've got a stack of outcome measures. Some of them are very, very domain specific. Specific things for the upper limb or for walking or not - or just the coarsest of all, the Rankin. There's lots of ways to think about outcome. As I said before, outcomes without any information about how the person was prior to you measuring their outcome are really problematic because they don't tell you anything about what happened prior to this point. I get a bit frustrated with studies that say, look, more people had a good outcome in this group than and this other group. It's like, yeah, but more people in that first group had a good outcome at the baseline but they didn't actually change, so how's that helpful? Anyway. Outcomes – problematic.”

“Increasingly takes quite a multi-dimensional view of recovery and outcomes to consider objective measures of the person's actual brain or body. As well as clinically assessed measures where usually a therapist assesses them and make some judgments, hopefully fairly consistently, about performance and how to grade it versus purely patient reported measures. You've still got, in my mind, there's three groups there of types of measures, and increasingly all three are used simultaneously to gain some insight.”

**Clinical Research #9**

“I think it’s probably more complicated than most people would make it out to be in research papers.”

“Putting new tyres on your car and measuring engine rpm as the indicator of success, right? Like, it’s - ultimately, it’s important that we move properly, but measure what you’re actually treating, and, if that’s responding, then go on and look at things. So, there’s some flawed - I don’t want to say - maybe flawed thinking, or maybe just the tools weren’t ready at the time to be able to measure things like that.”

“The timelines for recovery of sensorimotor function can be markedly different…Patients who had most of the recovery on a reaching task, so, a motor task, essentially, in six weeks, but then other people who, their sense recovery, they made most of it between 12 weeks and six months. So, you have these interactions which just, I think, say to me that we don’t know as much about sense recovery, we don’t know as much about cognitive recovery or language recovery and the timelines. That creates challenges.”

Clinical Experience Group:

**Clinical Experience #1**

“That's different for every patient but can they do the things that they want to do or need to do to live their life? If you're talking about more of a standardised measure, that's always hard.”

“I still look at those how much assist someone needs to do those things, but then when you talk about a more standardised outcome measure, I feel those are important as well so that you can compare.”

**Clinical Experience #2**

“I think if we don’t do the whole ICF we get stuck”

**Clinical Experience #3**

“I would say it does rely heavily to what that patient is telling me. What I’m seeing in my practice in terms of how well they're implementing that impaired arm back into their normal routine.”

“I think outcome measures have a role to help patients understand their recovery too though. I have seen, you know, a lot of patients go well, “I feel like I’m not improving, I'm not getting any better.” And I think that in that rehab journey, you know, even for me, as a person not having a stroke, I find it nice to know that I have improved. You know, able to lift a more heavier weight at the gym or I'm able to do another repetition or, you know, run faster. So, I think those objective measures are important and play a role in motivating a patient, but as a clinician I probably don't use them to be like “tick, recovery complete.”

**Clinical Experience #4**

“I don't think we have very good outcome measures to be able to say whether someone's getting that restitution style recovery. If I was if I was researching it, for example, or as a clinician involved in research. I’d be looking at movement kinematics.”

“In particular with real functional tasks as opposed to lab based task, where we're looking at motor control we're looking at reach and grasp and we're looking at a whole selection of on movements.

So, as a clinician I think FMUL is one of the better ones to give me a clue about in that early phase of recovering where they seem to be tracking in a direction where there's some positive signs of more movement patterns emerging.”

“if they're a bit delayed in the recovery. Let’s say it's a stroke patient who's showing good signs of upper limb recovery, but they were really set back early on, by...let’s say they're unwell with pneumonia or had other setbacks... So, they’re just sort of catching up but much later down the track”

**Clinical Experience #5**

“A measurable change in what someone can actually do with their reach, their grasp and then the activities that they can do. Obviously, at work we've used different outcome measures, and I think they're helpful to measure change but certainly not enough to tell you how to treat. I feel like, again at an individual level, to do them properly you need to...they kind of look very specifically at certain things and actually they aren’t tailored to that person's arm or condition. So sometimes actually about being able to achieve the activity and not just achieve it any old how. Actually, looking at quality and why they can or can’t.”

**Clinical Experience #6**

“I assess functional use of the arm, either using standardised assessment like the motor assessment log and stuff like that. I also take into consideration patients qualitative feedback. Yeah, so the thing is with patients when they come in they may set a different goal for themselves. They may say that I want to be able to use my hand to hold a spoon to feed myself, right. But then along the way they see changes and things happen, or their perspective change, right.”

“I put more weight on the functional use of the arm, okay. But at the same time if I were to use a clinical outcome measure, like say, for example, I look into the activity, okay. Then I realise that actually with this movement you should be able to perform that function. But you keep saying you can't do it, right. So that’s when I kind of also look into why is there is a discrepancy? Why are you not being able to use your hand when your movement is actually pretty good? Okay, so that's when I kind of - then again if you think about it, then again I am still putting a lot more emphasis on the function, I am just using the standardised measure to give me a gauge of how this person should be performing in real life.”

**Clinical Experience #7**

“I suppose a lot of our outcomes are actually tricky in terms of whether they are being achieved by a compensatory way or a restitution way. I suppose I’ll come back to - I probably would tend to have like a one-handed measure. So it might be something like a Jebsen or something like that to sort of get some timed dexterity.

If it’s something like round the shoulder - you know MAS is okay to a point, but sometimes you might need something where how long they can hold for, how many times they can do something in 10 seconds. I might make up little things like that to get a bit of an idea of the motor control of a particular segment. To see if that's changing over time. So I’d use a series of measures to see how that's changing and try to standardise it. Even if I’d made it up myself I would try and standardise it in the best way possible.”

**Clinical Experience #8**

“Try and cover the whole ICF. We do an impairment based one which is the Fugl-Meyer, we do a supposedly more functional one which is the ARAT, but also the CAHE which we quite like because it's by manual tasks. We do patient recorded outcome measure which is the ArmA and the ArmB, but that was really developed for spasticity but it's still quite a nice one. We use other various patient recorded outcome measures like the stroke impact scale and the self-advocacy questionnaire.”

**Clinical Experience #9**

“There’s the Fugl-Meyer and the Chedoke-McMaster outcome measures but I think ones that include functional tasks of the hand or an outcome measure that includes meaningful activities for the patient themselves, I think that’s really, if we want to be applying patient-centric care principals, I think that’s where we really should be focussing our attention on.”

Lived Experience Group:

**Lived Experience #1**

“I can pick up with the bits and pieces and start to heal it. Before I couldn't do anything with it. If I can pick up the glass.”

**Lived Experience #2**

“Some improvement in either dexterity or reach.”

**Lived Experience #3**

“Being able to lift my arm up, and to be able to move my hand, and move my fingers, pick up things in my hand. That would be those sorts of things I think I’d be looking for as initial evidence that things were improving.”

**Lived Experience #4**

“Well...say - the fingers would come alive, you know? Being able to see movement.”

**Lived Experience #5**

“it feels to me like when I can open and close my hand at will, that would be. I mean that's a long way off I think. But that’s sort of what I feel like. I tend to be a bit, you know, I get a bit flat about it. I can't my hand, yet. So therefore there’s not much going on.”

“I think so qualitatively, Yeah, I find that really helpful personally.”

**Lived Experience #6**

“The fact that there are certain things I can do that I couldn’t do.”

**Lived Experience #7**

“Nah, I’d rather just hug the grandkids!”

**Lived Experience #9**

“I would do certain things with my hand that I can’t do now.”

**Lived Experience #10**

“Not the numbers. Yeah. But yeah, when they - I guess, I have a example. So, I was trying to move my finger - oh no, I will not do it, but I couldn't do it, and then occurred, so, oh, that was a good measure.”

### **Subtheme 2C. Optimising capacity: knowing when and how.**

Preclinical Research Group:

**Preclinical Research #3**

“We focus also on strategies that promote compensatory techniques or strategies because, in the end it's what the patient perceives right? So, if true recovery becomes the ultimate goal that's Okay, and particularly from a scientific perspective, I think that is what we are aiming for. But again, to place it in the patient's perspective, the patient just wants to recover as optimally as possible and if that is achieved through compensation in a better and easier way that with strategies that could potentially promote true recovery, then we should go for that”

**Preclinical Research #6**

“This patient, this animal doesn't have the capability of moving beyond this way.” And then maybe it would be useful to just work on compensation, but I think trying to achieve restitution initially is really what one should try for.”

**Preclinical Research #7**

“Compensation can be a stage towards that recovery, it can also be a different kinds of compensation. Of course, it can be something that is interfering with that recovery, and really the question becomes when should you actually favour the compensation that's going to interfere with recovery because there's not any capacity for that recovery? And that's a big knowledge gap to fill in for our field”

“There is surely a severity of injury and motor impairment, maybe it's corticospinal tract injury I'm not just not sure, where there is likely to be little if any true motor recovery in terms of return of normal movement. In which case we should be throwing our massive efforts into what's optimal compensation right?”

**Preclinical Research #8**

“You want to say, "you want to have the perfect movement back" because this is - if you think about sports and like it’s all about - there’s one optimal solution. There’s not, there's 25,000? But I guess that if you think about it in the perspective of a lesion brain - like I often get back to this and there’s big wigs in the field that have written these - and people make a big fuss about we should have restitution and not compensation, I’m not convinced.”

Clinical Research Group:

**Clinical Research Participant# 3**

“You’re just not going to be able to remediate some stroke deficits. I wish we could – we just don’t have the really neurotechnology to do that. So I don’t see how you’d get away from compensation with where we are today.”

**Clinical Research #4**

“if the compensation is at the expense of further recovery, then I would say it's not a good idea. So we can solve movement problems in multiple ways, but if one way of doing it is the easy, lazy way. That gets the job done and that could be fine for the individual, but if in fact they have more room for true recovery, whatever that is, they could be stalled then at a place that is suboptimal for other things.”

**Clinical Research #6**

“My personal point of view is that within this window of 8, maybe 10, weeks for some people in which spontaneous neurological recovery is happening - That is the process of time alone, without any therapy influencing this repair. After 8 or 10 weeks there is always a matter of compensation or optimisation or adaptation. The question is if we are doing this within the first 8 weeks or 10 weeks are there any interaction effects with behavioural restitution and neurological repair.”

**Clinical Research #7**

“Their recovery is inevitably going to involve some compensation. I think the critical thing is that, if you reach the point where somebody is functional so that they perform activities in an everyday way, not in an exercise way, but they're actually using their hemiplegic limb in a normal way, or bilateral movements that they're using it without being conscious of using it, that it becomes automatic, then the recovery - that would be a point, I think, a critical point in their recovery.”

“I think the only thing is that if you do things and using compensation, you may inhibit restitution.”

“The structure of your nervous system will change as a result of the practice, the behaviour, that the learnt behaviour, so - which is why we try - as somebody who we think we have got the potential for true recovery, we might want to try and avoid them learning compensatory patterns because that will form new pathways, new synergies, new ways of doing things, which might inhibit relearning a more normal pattern, which probably won't be as good, as effective as the true recovery.”

**Clinical Research #8**

“If we're talking recovery, then I think the only sensible time to do that is in the first three months. Because after that the biological processes that support recovery have largely resolved in the motor system and improvements past that point are down to just garden variety learning.”

Clinical Experience Group:

**Clinical Experience #1**

“If I'm seeing someone let's say two weeks post stroke and they're starting to move, they've got some finger extension, they have some shoulder abduction, that's pretty promising to me so I'm going to try for restoration. If someone has zero motor in their limb it's harder. There's really, and to your question about intervention, like what do you do with their arm if it doesn't move at all? How do you help to even start restoring that? That's when it's hard.”

**Clinical Experience #3**

“I think early post stroke that really isn't in my mindset: compensation. Like, I wouldn't be the clinician who prescribes the sling on the stroke unit for a patient who has a hemiplegic arm unless I have had a very specific reason.”

“if they are really severe, compensation off the bat, should not be a goal as a clinician.”

“I would say though I wouldn't like to teach them or recommend something that's a bad habit for them to try and develop. I would rather try and use aids, or use the other/unimpaired side, rather than use the impaired side badly and maybe then not be able to pull that back later down the track if they are doing ongoing rehab. Yeah, I think compensation plays a role, and perhaps in an ideal world, less of a role early, you know, early post stroke. But yeah, the feasibility constraints overall mean that there is an element of compensation.”

“I think there's a line between promoting poor movement or bad movement and, you know, achieving a goal that might be really important to that patient. I think that it's not always possible. And I think intuitively as humans, we take the easiest route to do a task normally and sometimes that does mean that patients start to use their arm in perhaps not a normal way.”

**Clinical Experience #4**

“Once I’m getting an upper limb that's past the six-month mark, I'll probably be working with whatever I’ve got to try and see what movements are capable. And see what the patient is capable of practicing and how that relates to their goal.

**Clinical Experience #5**

“There's different ways to even use the term compensatory. So, compensation on one level could be that you're using a particular type of movement to achieve an activity. So, it's a compensatory way of achieving the same activity. Or it can also be that it's more: you haven't had for return a function so you're still incorporating that arm into a task in a way that's meaningful and assists the tasks.”

“Yeah so, it's compensatory, but sometimes I think people always think compensation is thinking of an alternative method. Where actually it can just be that you've got reduced function that you're using in a somewhat normal way.”

“Sometimes, by taking a compensation approach you see some of that motor restoration. Whether or not that's a brain level or whether that's actually undoing or unlocking something which is a weakness or a tone issue... I don’t know at that level, it’s difficult to tell, quite possibly it is...but the outcome is the same.”

**Clinical Experience #6**

“Right, so with somebody who is a lot more severe I usually don’t aim for more than shoulder and elbow recovery, okay. But for somebody who is a lot less severe I may even look into wrist and hand recovery. Yeah, so the balance is definitely different.”

**Clinical Experience #7**

“Compensation can be good and can be bad. So again it depends on there's often different ways to explore how to do something. So if compensation is actually putting undue strain on joints and ruining joints…That's not good. There's a place where you might have to adapt. So, it might have been where you actually have a larger pen rather than a smaller pen. I would still see that as a way of compensation. But you know you're actually able to still use what you've got in a better way.”

**Clinical Experience #9**

“It depends on their presentation as well as - or the severity of the stroke. Mild, moderate, severe and whether or not that - I think over time, I’ve gotten the better picture of this person’s - how people move is a pretty big signifier for what impairment. So you can kind of get a sense of someone’s moving through these really densely synergistic movements. Like I’m not going to be trying to focus on breaking apart these muscle groups for them to get normalised movement where someone who might be already showing kind of a lot of control, it’s just weakness or some flaccidity, then maybe.

It’s so hard and I’m probably not very consistent with that either. I think that’s also identifying this kind of gap in my knowledge as well as - and maybe there is hope for people with a very hemiparetic arm to return to normalised movement but I haven’t seen that with my own eyes yet, nor have I heard of it.

I have no idea if the literature is really starting to show that that’s possible but my kind of locked-in understanding so far is that there’s kind of this - yeah, you have your non-responders and your responders and yeah, I guess that would be how I would choose to approach someone is whether I have a sense that this person is going to be a non-responder or not.”

Lived Experience Group:

**Lived Experience #5**

“I would like to think of it as maybe it's a bit of a detour, but I would still be aiming higher towards the other goal.”
