# Supplementary material for: Considerations for developing complex post-stroke upper limb behavioural interventions: An international qualitative study
Source: Clin Rehabil. 2024 Jul 25;38(9):1249–63. doi: 10.1177/02692155241265271 (PMC11487871; doi:10.1177/02692155241265271)
Supplement: sj-docx-6-cre-10.1177_02692155241265271 - Supplemental material for Considerations for developing complex post-stroke upper limb behavioural interventions: An international qualitative study [file sj-docx-6-cre-10.1177_02692155241265271.docx]

## **Theme 3. Stroke demands personalised solutions**

Preclinical Research Group:

**Preclinical Research #2**

“We have to do a lot more basic work on the paretic phenotype…granularity work trying to relate certain physiological and anatomical data to behaviour.”

“Talk about hemiparesis, but before we start talking about the corticospinal tract or reticulospinal tract you have to have a very good characterisation of what hemiparesis is…With Parkinson’s Disease people are very clear about what you are trying to target. Are you trying to target the tremor, the rigidity, the bradykinesia, the nightmares, the cognitive problems, the balance problems? They all respond differently for example to DBS, they respond differently to dopa, right? So, you split it into its components. In stoke you just go – hemiparesis.”

“What is synergies? what is dexterity? what is weakness? what is spasticity? what is compensation? what is happening peripherally?... You need to do extremely detailed human neuroscience into the actual deficit and then map that to the best we can, anatomy and physiology.”

**Preclinical Research #3**

“If something works in one patient it doesn't necessarily work and the other patient. We know this! That's why you need to personalize it because there is no one standard therapy that's going to work for every patient. You need to identify patients. If you have a therapy that has been shown to be effective in a certain patient population, then you know: this is the patient population that could benefit from this treatment. But you should also identify the patient population that does not respond to that therapy. That means you have to find something else, or maybe there's nothing else. But it means that for every patient there could be a therapy, but there also could not be a therapy. It's not that there is one standard therapy that should be given to patients.”

**Preclinical Research #5**

“So there's paresis, hyperactive stretch reflexes, and there are abnormal muscle coactivation patterns that clinicians call synergies. So really trying to understand what drives that.”

**Preclinical Research #7**

“How you personalize it. Because common sense in everyday knowledge tells us that different people find different things more or less rewarding.”

**Preclinical Research #8**

“The other thing about stroke is the heterogeneity of the population. So, the animal models allow you to target specific subpopulations - so control very precisely for version characteristics…So, that increases your capacity to identify subtle changes that can get lost in human population.”

**Preclinical Research #9**

“We underestimate individual differences. It always has impressed me when we started making even these little focal strokes, that are not like clinical strokes - we do them and physiologically identified areas we know exactly where we're making the lesions, they are the same size, and yet we see a wide disparity in the way that animals react, whether it's rats, monkeys, or humans. It's a lot easier for us to demonstrate that objectively in the animals because of the lesions are virtually identical.”

“there's a lot of pre-existing anatomy that we don't appreciate and the physiological interactions that go along with that. We treat human brains as basically the same and all we have to do is look at percentage of the corticospinal tract that's involved. That's certainly one outcome that does seem to be correlated but a lot of the nuances are something that we haven't actually gained access to. We don't have the tools to yet to access what is different about these brains that respond.”

“It's striking, though: when you see animals that with the same lesions respond very differently, and we don't have a good answer.”

Clinical Research Group:

**Clinical Research #1**

“They’ve [medicine] gotten to the stage where they actually test the decision-making algorithm as to give to which person because they know that not everything is going to work for every person. Precision medicine. Bring the over into rehabilitation and be explicit about it.”

**Clinical Research #2**

“We act as if a stroke is a singular disease. We know it’s made up of lots of different domains, lots of different areas affected. But we tend to forget about another axis, the difference being severity. There could be no doubt that correlates of better outcome in the mild patients are not the same as the correlates in the more severe patients. There’s no one solution. Stroke is very heterogenous. I think the general principles apply.”

“If you have severe flexor synkinesia and Fugl-Meyer of 15 you’re going to use a very different apparatus to try to pick up a cup successfully, or not, than somebody whose Fugl-Meyer is 64 out of 66 and just has a little bit of interosseous weakness.”

**Clinical Research #3**

“I think it has to be highly personalised, from person – from individual to individual, and kind of personalised throughout the rehab experience, right, as they’re changing their capability, that’s going to be changed a lot too. So, I don’t think there’s a single one that fits into that.”

**Clinical Research #4**

“You have somebody with the same exact lesion, and one of these two pairs that are paired by lesion improves tremendously recovers a lot, and the other person recovers very little. So that says, okay, we need to consider the brain, but we need to consider more than just the lesion.”

**Clinical Research #5**

“I think we need to start using the word diagnostics in stroke. We’re missing a trick by not using that word. People are not taking us seriously because they don’t understand that, what we’re dealing with is far more complex than if you’re doing molecular biology or genetics, we’re doing something more complex. So, you need good diagnostics that give you then good prognostics, and then – I mean the kind of the crux of your question is about the hashtag plateau, right. At what point do you go, well you’re on the plateau so we just need to compensate. That’s a tricky question. I think you need the diagnostics; you need the prognostics.”

“I come back to this concept of diagnostics. I have two videos of men…neither of them are really able to use the arm. One of them, the reason they can’t use their arm is because they have profound weakness; you can see the scapula is wobbling about all over the place. They’re profoundly weak, probably got to be a bit of increased tone as well. The other video is a man who has been asked to make a cup of tea in the OT kitchen and he’s wondering around, and for all the world he looks like he’s got a flaccid arm, it’s just hanging by his side. He’s kind of wondering around, he’s getting all the bits and pieces and, at some point his brain decides, actually, I’m just going to use my right arm now and his arm comes up just - I’m not saying it’s perfect, but his arm comes up to stabilise an object whilst he unscrews the jar. The question is, why does that man’s arm not work? This is why I use the term diagnostics because the answer cannot be: because they’ve had a stroke. The fact they’ve had a stroke is irrelevant in planning how you treat that person. We need to know weakness, we need to know what their level of control is, because obviously weakness and motor control are two different things. We need to know about sensory loss, we need to know about cognition, we need to know about all kinds of things. If you put those two people into the same trial because their ARATs are roughly the same, in the same ballpark, why would we expect the same treatment to have the same effect in those two people? It makes no sense at all.”

“The stratification of patients based on expected outcome. The spinal cord people have done this very well, understanding the parameters that you need to measure in individual patients in order to understand more about where they’re likely to get to.”

“You can always get an ARAT or a Fugl-Meyer or a CAHE out of somebody, but it might be for completely different reasons, and then we throw them into the study, especially when the ‘n’ is 20, and we’re going to give them a bit of virtual reality or bit of, whatever the fashionable thing happens to be.”

“The SRRR thing about the how much, who, why, and what, you know, that we talked about. Who, that is about the diagnostics and the prognostics that we talked about.”

**Clinical Research #6**

“More stratification of the phenotypes in the background. Let’s stratify better at baseline for these different types. That’s the methodological point of view – when doing trials, we should do them much better.”

“We have to understand first the phenotypes of stroke patients much better in order to understand what type of therapy we should go with.”

**Clinical Research #7**

“I think that's a really difficult question because every patient is different and requires a different approach; that some people are probably more able to be independent in the practice that they do, other people need much more encouragement.”

**Clinical Research #8**

“Going from many people with one thing, this is one person with many things - two different perspectives on how to do this. One's driven by practice and the other is driven by principle, what's acceptable research design and conduct. It's really complicated.”

Clinical Experience Group:

**Clinical Experience #2**

“Diagnostically where we’re seeing upper limb, we really want to assess that arm to see what the impairments are…It’s not just about motor impairments and sensory impairments it’s also about having some acknowledgement about cognitive impairment, and also affect and mood and the impact that has on recovery. Then, we also need to break down once we know what the problem is, so what the impairment is, either is it apraxia, is it a pure sensory, is it a pure motor, or it is all three, have we got spasticity. So, we debate, discuss, what are the barriers for that individual participating in their activities, what is the impairment that’s stopping them doing that. Then, we move into: how do we treat that.”

**Clinical Experience #3**

“One of the biggest things is a good baseline assessment. I think that is very helpful to look back on as well. In a patient journey through the rehab process, it also helps me decide what I would want to do with them.”

**Clinical Experience #4**

“The ability to be able to feel and assess things and understand things about biomechanical restrictions, and spasticity, and painful structures, and all these things that probably add nuance that really affects your clinical decision making.”

**Clinical Experience #5**

“You can have an arm that if you measured the power, sensation, tone, and all those kinds of physiological factors, in one person versus another person, their individual kind of lifestyle the demands for the use of their arm, their individual personality, their wants. All those things that makes them who they are.”

**Clinical Experience #7**

“Places to start would be very much individualised.”

**Clinical Experience #8**

“Two sets of eyes and two lots of clinical reasoning at the very least are always better than one. It's really helpful.”

**Clinical Experience #9**

“I think is not just a science but it’s also an art in that clinical piece of each individual therapist and how they reason through what they see in the presentation of stroke - patients with stroke that they come across…The clinical intervention that a therapist applies is so individualised.”

Lived Experience Group:

**Lived Experience #1**

“Everybody works differently, and some of them work in one way and other don’t.”

**Lived Experience #2**

“They [therapists] all have different approaches, certainly.”

**Lived Experience #3**

“One of the frustrating things in hospital is, every question you ask the answer is, oh, everyone’s different.”

“I think probably having a variety of different techniques available to try just to see if one works better than the other, I think is quite beneficial.”

**Lived Experience #9**

“But they sit with me we go through the process: are we getting better? You know, and I did meet a couple of people, you know? They had different injuries or symptoms to mine.”

**Lived Experience #10**

“I think it's different for me. It's not just my hand. If it's just my hand, maybe I wouldn't care as much, but the aphasia and the speaking, it's in moves in my brain.”

### **Subtheme 3A – Condition specific considerations**

Preclinical Research Group:

**Preclinical Research #1**

“Depending on stroke sub-type and location…They’ll have changes in perhaps cortical plasticity.”

**Preclinical Research #3**

“There are some critical factors like the corticospinal tract, I mean if you're looking at motor recovery, and you have no corticospinal tract, then the chances of good recovery are almost zero…are zero, we know that already.”

**Preclinical Research #6**

“Post stroke fatigue and that turns out, of course it that's a big problem, but nobody has a clue what it's all about.”

**Preclinical Research #9**

“I think using relatively crude assessments of using motor evoked potentials or imaging to look at the lesion load on corticospinal tract. Those are fairly straightforward, and there are some general relationships with clinical status and potential for recovery.”

Clinical Research Group:

**Clinical Research #1**

“The biggest thing in the literature is stroke severity. Overall stroke severity. I actually just gave a lecture to the PT students here this morning about what are the prognostic predictors of recovery, so I’m fresh. So, the more severe the patient is, the less recovery and the longer it takes. The more mildly affected, the quicker and the more recovery occurs.”

“The higher the number of non-motor impairments, the less likelihood that they’re going to achieve functional independence.”

“Of course, they would be individually titrated to challenge the impairments that you’ve identified in the patient through your evaluation but done in such a way that they’re trained.”

**Clinical Research #3**

“You have a bigger stroke, you have less aggressive intervention, well of course you have a worse outcome. So those are other things that I think we need to be considering when we’re deciding who and when and how people get therapy.”

“I think we also need to understand in those patients, kind of the magnitude of overall damage in the brain. I think that’s going to alter how and when they recover.”

**Clinical Research #6**

“that’s not surprising because looking back to some of the animal studies there might be some parts of the brain that may be really conditional for this spontaneous neurological motor recovery. Then we need first, to understand how these parts, and how consistent these parts are at in defining spontaneous recovery.”

**Clinical Research #7**

“They're probably going to have other problems as well as their upper limb problem. So that might be an added complication and probably actually the frustration of not being able to do very much and not making very good progress.”

**Clinical Research #8**

“The processes in your brain initiated by stroke, they happen whether you want them or not, whether you like them or not, whether you understand them or not. There's a lot that we can do bugger all about…There's so much we can't do much about. So much of somebody's recovery and end outcome depends on how big the stroke is, exactly where the stroke is, what critical white matter connections has it destroyed?”

Clinical Experience Group:

**Clinical Experience #1**

“It's so frustrating to me because I feel like that's [neglect] a really big limiter. When people have great awareness, when they're already starting to bring their hand up onto their lap or onto the counter, I'm like - I get so excited because I'm like okay, half the battle, we're there already.”

**Clinical Experience #3**

“I would say the lesion sites, where they have their stroke. Whether or not your corticospinal tract might be intact or not, or partially intact. I would say how big their...I think there is not enough research out there about size of lesion but I do think, a huge stroke vs one that is smaller does have a role to play. I think location over size, I would say I’ve seen strokes it look huge on scans and they have quite good and hand recovery versus, a patient who's had a stroke, in a very specific area that's impacted, those pathways then they're not going to have that same level of recovery.”

**Clinical Experience #4**

“Then you've got other people who are severe because they've got major motor planning problems, cognitive issues, neglect, anosognosia, recognition issues, and the list goes on and on and on.”

**Clinical Experience #5**

“Abnormal tone. So, you know, in the presence of any abnormal normal tone and particularly that has led to any soft tissue shortening, loss of range, pain. So, anyone who's not able to move their shoulder, with a really stiff shoulder might need a dilatation, or whatever. So yeah, definitely all those kinds of structural changes can be a real barrier.”

“it's all those capabilities that can affect recovery. And we haven't talked a lot about sensation. Sensation’s also hugely important, and I know we've been saying that for quite a while.”

**Clinical Experience #8**

“Fatigue, particularly early on which is a real bummer because that's when they say your neuroplasticity is best. Gives with one hand, takes away with the other a stroke.”

**Clinical Experience #9**

“The stroke itself, like where was the stroke, how severe was the stroke? Was it a clot? Was it a bleed? Was it both? How was it medically intervened? What was the timeline there? So, I think the stroke itself is so crucial or the nature of that stroke is so crucial to our understanding and hopes for recovery.”

Lived Experience Group:

**Lived Experience #3**

“I mean, you know that’s the case that everyone is different, but just being able to give you a range of possible outcomes I think is very valuable.”

**Lived Experience #8**

“Well, I think, when we first came to treatment, I asked, I wanted to know what is the time frame? What's the prognosis? But I came to realise as well, that that’s only just a small part of it and it's not very helpful to have a specific date or just to think or have in my mind that in six weeks, I'm going to operate much better. It creates a false expectation.”

### **Subtheme 4B – Person specific considerations**

Preclinical Research Group:

**Preclinical Research #3**

“I think older age people recover less well than young people. This has to do with the plasticity of the brain. I think if you have comorbidities, this will affect the recovery process. Everything needs to be in an optimal state to promote recovery of the brain, the vessels, the neurons, the supporting cells, the entire motor system. So, if any of those parts are less functional it will affect the entire recovery process.”

**Preclinical Research #5**

“There's the whole psychosocial aspect of things as well that can make things harder, the socioeconomic side of things.”

“Not even looking at the person, but even the other comorbidities, etc., that people might have cognitive deficits, visual deficits.”

**Preclinical Research #8**

“For human and I think that’s where on top of it [the stroke], you’re dealing with depression. All of these humans, they have all of this cognitive burden on top of the physical burden. Animals don’t give a sh*t that they have problem moving their hand.”

**Preclinical Research #9**

“Humans experience depression and in ways that animals don't and we have a lot more motor learning capacity in the sense that we can we can develop compensatory strategies in a different way with some additional cognitive executive functioning that the animals may not have.”

“We can't treat all brains the same. That normal brains are normal brains, and injured brains are due to the particular injury they have. People come into their strokes with very different pre-existing traits, and we don't quite appreciate that.”

“Human brains have an additional capacity for cognitive function that sometimes can really override in a positive or negative way.”

Clinical Research Group:

**Clinical Research #1**

“You’ve got age of the nervous system, right? So, the same stroke in a child does not produce as debilitating deficits as it does in an adult because of both the nervous system and the rest of the physiological systems that allow the child to be more capable and more active. The existing comorbidities of the person. Again, here in the States, our average age of stroke is probably about 60, which I think is quite low compared to what you guys see. We do not have a national healthcare system so there’s a lot of people that have coexisting comorbidities, many of which they’re not aware of and are poorly managed for whatever reason.”

“The number one thing to do when you’re trying to pick an intervention is to ask the patient what they want. So whatever intervention you design, it needs to be tailorable to what the patient’s goals are.”

**Clinical Research #2**

“Something that we don’t measure much that’s huge to normal function and post stroke function is the social circle, social factors… the social network side showed that it was related to outcomes. I think that’s huge. It should be huger. It doesn’t really fit with our models of M1, CST muscle. But the social bone is connected to the motor bone.”

“Similar to depression, but joy - a lot of people after stroke, they lose the joy in life even if they’re not clinically depressed. Society spits them out - at least US society. It doesn’t have to be that way.”

**Clinical Research #3**

“Pre-morbid health and activity levels. They maybe diabetic right now, maybe they had COVID, or have COVID. You know all these other things happening that are making them kind of sicker to start with. I think that’s part of it.”

“Patients who were more active to start seem to have just a much different recovery pattern. I know it’s been touched on in the past, but that’s something that certainly we need to think about. I think that we also need to consider differences in both sex and gender, as we’re kind of contemplating these recovery profiles and trying to think about how to best prescribe interventions. Then a calculation of brain health or brain age is going to be really critical in here as well. We all know we have people that have healthy or stronger looking brains, and they recover better. They may be younger, but they may also just have a younger brain age. There are now pretty cool ways that we can calculate that out and account for it, as we think about who to give what intervention to, or how hard to provide them an intervention with. So, I think we could be much more elegant in the ways that we do these things than we have been in the past.”

“We see baked-in biases against women. they’re significantly less likely to be offered aggressive interventions after a stroke. So TPA or mechanical embolism, or infarct extraction, a woman is much less likely to be offered that. So, the net result is she has a bigger stroke. I think that’s something else that we need to really think about.”

**Clinical Research #4**

“A good example of that is the sort of brain health idea right now. There are likely many others that the science will eventually come up with.”

“We need to consider the brain, but we need to consider more than just the lesion. We need to consider whether this is a healthy brain to start with that had a stroke. So, there's an example where you focus on the brain, but maybe we're not focussing on the right aspects of the brain to really capture what we see in terms of individual differences in recovery trajectories.”

**Clinical Research #8**

“Do emotions have some kind of massive effect on their recovery and outcome? I don't think it's true, or if it is, it's only through other things like engagement and therapy, ability to sleep. But creating that kind of causal link places a huge burden on patients. It's totally unfair.”

“What is the person's pre-existing overall brain health? Where are they at? All of those things.”

**Clinical Research #9**

“Depression is a common one. Almost a third of patients that I see will have depression in the first six months after stroke. If that comes on in the first month or two after stroke, it can be disastrous, right? They don’t want to - they start and they - you get them into inpatient rehab, and they want to do it, and then they get depressed, and it’s a downward spiral from there, right?”

Clinical Experience Group:

**Clinical Experience #1**

“Someone's age, what's their social support, how motivated are they, what's the motor recovery been so far? If I'm seeing someone let's say two weeks post stroke and they're starting to move, they've got some finger extension, they have some shoulder abduction, that's pretty promising.”

**Clinical Experience #3**

“There's an element of what that person was like pre stroke. Probably less so than perhaps an exercise point of view, but I think a mindset around rehab and responsibility for your own behaviour. How dedicated or motivated you are to improve. And that might be a post stroke think too, but pre stroke, I think those patients who, you know, had a really good personal internal motivation prior.”

**Clinical Experience #5**

“A lot of it is to do with socio-economic, and socio-educational kind of situation. And I feel like sometimes when I've seen some interventions or research come out that it feels like it's very much based around people with reasonable support and access to things. Those groups should have maximum access... but quite often they're very good at finding things anyway. For me it's the people who don't have a lot of family, don't have a lot of money, don't live in an area where there's good access to rehabilitation. I think actually from a sort of social political point of view, I think there's something about your background.”

**Clinical Experience #6**

“How they perceive themselves as a person. Yeah, so if even before the stroke they have always been not loving themselves so much, after the stroke it becomes worse. That affects their recovery because nothing they do is good enough.”

**Clinical Experience #9**

“Circumstance, right? It’s the internal and external circumstance of the stroke so unique to that person would be - you know, what their premorbid and postmorbid cognition and education, psychosocial. Do they have the time? Do they have the means to engage in as much therapy as needed to recover as if they’re - like what’s - is their brain and body in optimal health to facilitate tissue recovery?”

“What is their general health? What is their age? Prior - like who were they going into this experience? Were they a healthy person and the stroke just happened from maybe another surgery or whatever? Or were they chronically co-morbid with all these other potential processes happening? Like if someone already has type 2 diabetes with end-stage tonnes of peripheral sensory loss to begin with? Right? So, the person themselves.”
