# Supplementary material for: Considerations for developing complex post-stroke upper limb behavioural interventions: An international qualitative study
Source: Clin Rehabil. 2024 Jul 25;38(9):1249–63. doi: 10.1177/02692155241265271 (PMC11487871; doi:10.1177/02692155241265271)
Supplement: sj-docx-7-cre-10.1177_02692155241265271 - Supplemental material for Considerations for developing complex post-stroke upper limb behavioural interventions: An international qualitative study [file sj-docx-7-cre-10.1177_02692155241265271.docx]

## **Theme 4 - Upper limb recovery requires complex interventions.**

Preclinical Research Group:

**Preclinical Research #1**

“To engage true neuro-rehabilitative therapy for severe stroke patients requires assistive devices. I think it requires distinctly different reward and reinforcement paradigms and probably a prolonged and distinctly different neuro-rehab environment.”

**Preclinical Research #2**

“It has to be exciting, it has to be social, it has to be motivating, it has to be cognitively challenging, it has to be cardio vascularly challenging, it has to be difficult, it has to be beautiful.”

**Preclinical Research #4**

“Talking about enriched environment it is a combination of social stimuli, sensorimotor stimuli, space…”

“Most likely it [a complex intervention] leads to activation of multiple repair mechanisms. On top of the particular therapy content you are testing.”

**Preclinical Research #6**

“I think that kind of background is something that may be needed to be brought in more in the human side, where they become more of a complex intervention, not just a single thing that you're doing. The enriched environment is, I guess, the ultimate expression of that more complex intervention.”

“I think I think we really need to start using much more complex interventions. Every single study that we've ever done where we've done combinations of different interventions, be they enriched environments with reach training, or training with exercise, or training with stem cells, the combinations always work much better.”

**Preclinical Research #7**

“Maybe it's something like having people to rehab together and social groups, you know? Enriching the social environment. I know that there's music-based therapy approaches that are that are doing that.”

Clinical Research Group:

**Clinical Research #2**

“There’s assessment and there’s monitoring and there’s psychology, education and on and on and on. So that’s one framework that I think pertains to your question which is if the patient just walks in and all you do is say, start moving as many times as you can and I’ll be back in an hour, you’ve missed the boat. It's more than high intensity activity-based therapy. Good rehab therapy pertains to lots of different categories of experience.”

**Clinical Research #3**

“You put all those elements together, it’s kind of a complicated soup, but I think they’re all really important for the whatever-it-is you’re testing to be successful.”

**Clinical Research #5**

“We’re talking about a complex intervention, and the problem with clinical trials of upper limb rehab that just focus on reps is they are adopting and - it’s much easier to do - but what they’re doing is they are using the biomedical approach to designing a trial.”

“Removing the shaping or the clinical reasoning and you’re removing the transfer package and the coaching bit, and so it becomes something else. It’s then not upper limb rehab in the way that we do it, and that’s why I kind of look at those trials and go - they’re all really underwhelming, and I think they’re underwhelming because they’re just not studying the thing that we do. I think that’s the problem.”

**Clinical Research #7**

“But [dose] that's just the tip of the iceberg. It's motivation of the patient, it's the skill of the therapist, it's the technologies that they use, it's the number of repetitions, the usefulness of the movements, the way of monitoring whether these are normal movement patterns. A whole host of different things.”

**Clinical Research #9**

“It’s kind of my blundering summary of the psychosocial aspects of rehab. So, then there’s some components we talked about previously, which is dose…content, engagement.”

Clinical Experience Group:

**Clinical Experience #5**

“It’s the entire process. And I think what I’ve seen is a lot of programs or a lot of approaches is: they'll be very focused on just lots of repetition of movement, or just practicing functional tasks, or lots of adjuncts. And I kind of feel like you need everything.”

**Clinical Experience #8**

“I very strongly believe it should be a multidisciplinary team approach. Because, as you know, each individual discipline brings their own strength.”

Lived Experience Group:

**Lived Experience #2**

“it’s a little hard to itemise what’s happened because the therapy has been broken up into many, many – several segments.”

### **Subtheme 4A: Feuling Engagement**

Preclinical Research Group:

**Preclinical Research #1**

“I think the idea of joy. And so, if you talk to patients especially those with neurodegenerative diseases like Parkinson’s but you see it in stroke also. We all enjoy moving, that’s why we run, and we go mountain biking and we do all these things. We enjoy moving through space. People with stroke and degeneration in motor circuits lose that. And that’s a large and underappreciated element in lack of compliance to our therapy. And possible a lack of cellular reinforcement.”

“The standard feedback reward mechanisms that we use are essentially, to use an American term: “atta boy” or “atta girl” the kind of therapist based positive verbal reinforcement. And that’s just not enough.”

“There is just not enough reward for a patient at home to sit their and do those tasks over and over again. So, some have posited that a way around this is VR, gamification to try and generate a reward signal from task completion or maybe seeing a funny gif or video upon task completion. But there needs to be consideration of how we engage reward mechanisms.”

“I think it [treatment] requires distinctly different reward and reinforcement paradigms.”

“I think the idea of joy. And so, if you talk to patients especially those with neurodegenerative diseases like Parkinson’s but you see it in stroke also. Patients, and we all – we enjoy moving, that’s why we run, and we go mountain biking and we do all these things. We enjoy moving through space. People with stroke and degeneration in motor circuits lose that. And that’s a large and underappreciated element in lack of compliance to our therapy.”

**Preclinical Research #2**

“So, it’s fun! No one is going to lift a glass of water a thousand times. No one is going to open a closet 500 times. So, you need to find a way to reproduce the animal literature by having tasks which are fun and enjoyable. Enjoyable in a way that goes beyond bribing with drops of juice.”

“It’s the distinction between exercise and sport. Sport is more than exercise. What you have to do is come up with a neuro-sport.”

**Preclinical Research #5**

“It's essential, you know this, you’re a PT. It’s essential, how many of your clients go home, doesn’t matter if it’s a stroke or muscle skeletal, what do they do? They never do the exercises. Or they do it for a few days and then it kind of lingers off. My hope is, and that's something I need to prove, that doing it in a gaming environment - particularly also with younger generations that grew up with this - is a whole different ballgame. Now it's something on the phone, they get a score etc. I think it's essential, frankly. This way, it becomes not like a boring thing, it becomes an enjoyable part of your day. You do it two times a day, we can change the games to keep it all fresh. But at the same time, you get some therapy.”

**Preclinical Research #6**

“One of the big differences between the preclinical and clinical is that you know we can motivate our animals through very mild food restriction. You obviously can't do that in people. And at least I don't think you’ll get ethics approval. Although it might be fun to try. But that [food restriction] gives us more of a uniform motivation. I mean, we can't talk to our rats and say look, you know, “Mr Rat, unless you do all these repetitions you're never going to get better and you'll be impaired the rest of your life.” So we use food motivation. But I think that's a big difference and maybe clinicians need to think a bit more about how they can up the motivational level of their subjects. I mean, obviously some people are extremely motivated. But I also imagine that there are lots who are not very motivated as well.”

“I don't know quite how you do that as it may be through specific types of coaching and things like that, like, maybe you really need to bring in some people who really work with athletes and others who are really good at motivational speaking and inspiration. And then maybe also have to limit or better educate family members. And those who seem to often just sort of counteract a lot - you know? Not encouraging their family member to be active, and to do, and push themselves, which is really what they need, they need to do so. I think that whole motivational thing is something that needs to be thought a bit more about on the clinical side. Like, how do you try to get more like what we, we can achieve with mild food restriction.”

“I think it's [motivation] all very much involving meso-limbic, meso-cortical dopamine systems, you know projections to the nucleus accumbens. You know, prefrontal cortex and so on. You know in rodents or in order to humans, I mean it's obviously more complicated in humans, but I think the fundamental drive is neuro chemically the same.”

**Preclinical Research #7**

“I don't know what is likely to engage the reward systems in humans, as a scientist that doesn’t study humans, but I think we can apply a lot of what's known about reward systems to the idea. It is likely that telling someone that you've done great job is rewarding. Competition is rewarding especially if you're the winner. Actually, we are studying that in rodents. It does help facilitate. Food, sex, people, music for some people.”

“Hopefully talking to someone who actually knows more about that reward. I know reward is a positive modulator. But there is actually really mature research on the basic side, as well there’s plenty of clinical, or at least human, research on it.”

**Preclinical Research #8**

“I have a good one for humans. It’s motivation. There needs to be a reward system. You guys are kidding yourselves thinking that just talking loud to the person and clapping your hand and saying good job is really motivating the person. I think there is a little bit more thinking that needs to be put into that. I think motivation should be considered like a real factor.”

“I think that you guys need to talk to the reward system people and find a way that you’re engaging that reward system way more than you’d doing now.”

**Preclinical Research #9**

“Food is a great motivator, and you know anecdotally we see this all the time. The palatability of the food has a lot to do with whether those animals are going to do work that day, regardless of their neurological state. So, we will schedule their food, so they get most of their food during those behavioural sessions. We can do it efficiently, and it would be very difficult to design studies, without some type of restriction, time restriction and food delivery. So, it is a big motivator, and it can be a motivator in people too. But there actually haven’t been too many studies when people look at that.”

“How can we motivate people the way we can motivate our animals? Well, either use food or sex and I haven’t found a reasonable way to use sex as a motivator so! [laughs]”

“I think it's something that I don't think has been explored a lot. To use some other kind of motivation for people, I mean of course, a lot of psychology studies that use money as reward and those seem to be motivators for cognitive processes, for example. As much as cognitive processing plays a role in in motor control as well, that there may be some clever ways that we can tweak the motivational properties and may increase attention and so forth, we haven't really paid attention to that a lot.”

Clinical Research Group:

**Clinical Research #1**

“The social network or the social environment in which the individual lives. You can imagine that could be either helpful or hurtful, and that’s hard to tell. Like you could have a spouse who’s pushing you to do a lot more and that could be really helpful. You could have a spouse who is doing everything for you and they think they’re helping you but they’re actually inhibiting your recovery.”

“One of my collaborators is a psychologist by training. He does a lot of research design and statistics for this. He was funny. He’s like [redacted], why did you think you could change people’s behaviour? I said, because that’s part of my training as a PT, we tell our students we can change people. [Laughs]. So we have these rosy coloured glasses on, I think, that we can change people’s behaviour but there’s a whole science of health behaviour change that suggests that it’s actually really hard to change people’s behaviour. That comes from nutrition, physical activity, smoking cessation. We can see it now with the vaccination behaviours. All kinds of crazy things. So behaviour change is hard. I don’t think it’s a question of us motivating the patient per se. I don’t think the patients are unmotivated.”

“I can’t speak for everywhere else, is that it’s within the medical model. People come in and they think that [redacted] is going to fix me. That’s a very different model than what I would call the coach or the music teacher model where the expectation is I’m practicing by myself and then I go in to see [redacted] once a week or whatever and I show you were I’m at and then you help me figure out what I’m going to do to get a little bit better because I want to play some sort of song.”

**Clinical Research #2**

“If you just do rehab solid for an hour, high intensity, it gets boring. You have to pepper it with something [education]…It breaks up the tedium.”

“[education] to promote engagement, to get people interested in their disease. People marching down the street for AIDS and breast cancer, but stroke is a disease of shame. People don’t want to talk about it.”

“Patients really don’t know a lot about their disease when it comes to stroke. There are a couple of studies I'm fond of pointing out where people admitted for a new stroke or suspected new stroke were asked, can you name one sign or symptom of a stroke. Fifty per cent could not name one sign or symptom. It’s not that they’re bad or dumb or anything, it’s just if you asked me how a computer or a car works, I don't know. They just don’t know. So, by giving them information about the disease we’re empowering them. We’re demystifying, maybe taking some of the fear out of it.”

“It has to be fun. So people want to come back, I can’t wait to do our thing the next day because it’s fun. It gets old after a few months but it’s fun while it lasts”

“Easy access. It’s high motivation. We gamify. That’s I think important.”

“Another is not often considered but if you think about the literature you see for physical therapy, same as with pills, patient compliance is about 50 per cent, 60 per cent. [if you just] hand it to the patient and say, go do this now, the patient is like, yeah right. Then they go home and they’re tired, they’re confused, they’re lazy, they’re terrified, whatever, they’re busy. They’re trying to find a way to eat. People don’t do it. So…it has to be easy to access and easy to use.”

**Clinical Research #3**

“I think that sometimes patient engagement, even though it’s so critical, it’s very tricky.”

“Then the other piece that is critical and very hard to do, is engagement. So, it’s getting that participant, that patient to be a very engaged in the intervention is going to be critical. So, finding something they enjoy practicing, that is rewarding, that is often difficult.”

“So, if it’s an actual clinical intervention, you want to reward the patient. If you are working on something that they’re engaged in and interested in.”

“Getting a score, so you can tell, you get that what’s called knowledge of results, right, so you know how well you’re doing on a particular task. But I do think that it’s kind of critical to keep it very clear with the patient, like what success is, so that they can, I guess, be implicitly rewarded by achieving those particular goals. If you’re working with them to set those goals, then that should be a very clear relationship.”

**Clinical Research #4**

“They're going to lose motivation because they're not going to be - it's going to be a struggle.”

“I think that motivation is an individual thing. It's sort of like quality of life. You have to find out what motivates an individual, right? I mean, some people are motivated by just getting a point or two. Or some people are motivated by monetary means. Some people are motivated by just having the success. So I think people are different and you kind of have to figure out what motivate - what drives people.”

“That's probably the best way to get motivation then to assume that everybody's going to be motivated by getting a little star on their [laughs].”

“Especially older individuals. They have - there are different things they're motivated for, right? They're not the same as the gamers that make these games, right?”

**Clinical Research #5**

“But it does lead to this other issue about how long can you keep treating patients, because why is it that stroke patients in this country, and I think everywhere else, get great acute care, generally, then they get their inpatient rehab, then they get their six weeks of community, maybe their six weeks of early supported discharge, and then maybe they get a bit of community. Now, granted, there are pockets where they’ll do better than that, but the treatment people get is time limited. So, why is that? That’s not the case for any other neurological condition, and it’s because this idea that stroke is a one-hit condition and you do your recovery over your three to six months and then you plateau, and I think people look at that line and they go, well you’ve plateaued so therefore you’re not going to improve, therefore we’re not going to treat you. I mean it’s a kind of – it’s a very – it’s not logical, that line of thinking.”

“They should receive the appropriate treatment for as long as they’re willing and able to participate, and as long as they’re showing measurable benefit, and I think people are thinking about that in terms of those recovery curves, and I think they’re also thinking about it at the impairment level, and I’m fully accepted at some point you may not be able to eke out any more improvements at the impairment level in some people. But can you take anybody, however long ago they had the stroke, and help them at the level of activity? Yes, of course you can. Can you take any stroke patient, however long ago they had the stroke, and help them at the level of participation? Yeah, of course you can. I mean those two things are self-evident. So, why do we say, there’s no point treating people six months after their stroke because they can’t benefit. It makes no sense.”

“You can really do anything in chronic stroke patients, and that should open up the door again to patients being able to demand treatment for much longer periods of time.”

**Clinical Research #7**

“That some people are probably more able to be independent in the practice that they do, other people need much more encouragement.”

“It's very difficult to measure motivation and I've never - I don't know of any really good measures of motivation. But I think it's probably worth looking at the sport literature because I think that's something which is considered a lot more in that than it is in the clinical stroke rehabilitation where - and I think - it's not my field but I'm sure that there is - there are studies about somebody's performance when they're motivated being a lot better than when they're not motivated. But just purely sort of anecdotally, from clinical experience, knowing patients who are highly motivated and how much better they do than patients who are, let's say, depressed, who are not able to, don't have the confidence, don't have the ability to put a lot of effort into their recovery, seems from experience to make a big difference. So, I think we tend to - whether it's in stroke rehabilitation, in sport, in everyday life, what we know what we can achieve when we're highly motivated is hugely more than when we are just doing it by rote, sort of thing. But it's not my area, so I guess there will be psychology papers and certainly sport psychology work in that area.”

“I think probably the most critical thing is the motivation from feeling that you are improving. That's, I think, probably the most powerful thing that any patient - or any human being gets. Whether you're training to run a marathon or whether you're trying to move your - grip of a - you know, grip a ball or release something or - however simple or however high level it is, feeling that you are making progress towards your goal is the most important thing in encouraging you to keep at it. I think that's one of the frustrating things for upper limb patients with severe upper limb problems.”

“That might be an added complication and probably actually the frustration of not being able to do very much and not making very good progress; obviously you adjust the tasks that you're giving them so that they can get success, even if it's a very small movement. I think maybe the use of FES for very severe patients is something that's worth doing because that's encouraging for them to actually see some movement.”

**Clinical Research #8**

“You talk to our community colleagues and it's just like, well, we really have to help make the transition. From my perspective, information is power. Information is very powerful and enabling for people, whether they’re the person with stroke or their family or their clinical team.”

“if patients aren't supported to understand their possible outcomes and they're allowed to just focus on the best possible thing in their mind, often it is months down the track that they finally come to the realisation, this might be it - wow. That can come with a huge amount of disappointment and a sense of embarrassment that they had their hopes so high and that they worked so hard for nothing as it turns out. They go through all that without us because they've already left our care. We're not even there to support them as they go through that acceptance phase of their new life. We're not there to help them reconstruct their identity having accepted, wow, this is how I am now and how I will probably be pretty close to this for the foreseeable future.”

**Clinical Research #9**

“So, probably the number one thing is actually engagement in that, if you cannot get someone engaged in what they’re doing, then they’re not going to improve, or what improvement you see would then be termed by many people as spontaneous recovery, right? You’re just going to lay in bed and not do it So, I’m sure you’ve seen this, you [go and] look at Mr Smith or Mr Jones, and they just don’t want to do it. That happens. For me, doing consults, if somebody does not want to put the effort in, it typically means they don’t get inpatient rehab, and it often means they’re going off to a long-term care home. So, I mean, that’s on the bigger level for rehab in general, but, for the upper extremity, sure, if you’re going to have somebody do something two-plus hours a day to try to get better, it better be fun. It better keep their attention, right?”

“So, contextually relevant, engaging - I think those are probably two of the big things. Then the mechanism of delivery has to be able to support the fact that the person going through this is going to have ups and downs. So, again, part of that engagement is encouragement, when people are doing well, but also as they start to, you know, go through the usual ups and downs, that you’re continuing to provide that encouragement for them, so that they stay engaged.”

Clinical Experience Group:

**Clinical Experience #1**

“Also appreciate that there's so much education that we're also providing that that's just as important as actual physical movement.”

“I think that because it's brand new - some are brand new, right. They've had a life altering traumatic event and there's a lot that you need to learn not only about your body and your injury, but moving forward how are you going to manage this new body? How are you going to tell others how to help you if you need it? How are you going to advocate for yourself? Those types of things. Equipment too, right? Then just for buy in. I explain why I'm doing this particular intervention. Especially when you're having someone pick up blocks or pick up a cup 100 times. I mean I need them to understand why I'm having them do it 100 times.”

“Okay. I think ease of I'd say set up, right, so it doesn't take much time to start the intervention from a clinician standpoint. I don't know, something that works. Something that I know. Even if you told me it was going to take six weeks, let's say, but it works, then I think it's worth it.”

“That's what I mean about like something that is easy to - and in the case of a device, if it takes 20 minutes to set up or you have to constantly problem solve, the Bluetooth is dropping or whatever the case may be, then it's no longer helpful. I might as well just do what I know to do also works as a conventional OT.”

“it's so dependent though on your patient. Are they motivated? Do they want to work that hard?”

“I've seen what good family or friend support does for someone, right, when they have a lot of visitors or when I know that their discharge plan, right, they're going home with someone who can help them or someone who shows up for a family training and who is invested in their recovery. I think that makes a huge difference in how far someone will recover.”

“we have to rummage around and get the two in the cupboard and share them across the whole ward and all this sort of stuff. You go that's just - maybe everyone who has a stroke could get one of those and then we just teach them how to set it up. Rather than us spending all our therapy time to teach them how to set them up. They're really - they're not that complicated, seriously. Like again we could get some really - some very good programs to go to help sort of work on things.”

**Clinical Experience #2**

“Giving them the tools to be able to communicate to their therapists, or their carer along the whole pathway, how do I optimise my recovery.”

**Clinical Experience #3**

“There’s a big role about education and coaching that I think that would be important as part of what does good arm and hand recovery mean to me. It’s about ensuring that people get the right information.”

“That coaching and that education about how somebody’s going to use their arm on a daily routine perspective then we just don’t get that success at self-efficacy.”

“A lot of patients do need ongoing support, but some are able, if given the right knowledge at a level, whether that is whatever dose you give them and we have an enriched environment to give a high intensity, but if you give people the knowledge they can carry on training and can adapt, if they’ve got good self-efficacy.”

“I think that helps your arm and hand recover if you can learn. We haven’t done a study on learning, but it would be good to know. I mean it’s the same with any form of education and coaching if you’re not receptive and you’re able to retain that information whether you’ve got strategies or no strategies then that’s the same for cognitive rehab, it requires a slower level of intensity, and you won’t get good arm and hand recovery.”

“So, you know those patients who are perhaps...have their communication intact, they can follow one stage, or at least they can follow a command: a verbal or visual command. I would sort of try - and they have movement - I would try to get them to start to engage with their arm and to start to move it as much as possible.”

“Get potentially some ability of that patient to do some of the movement themselves. Which I think is a really motivating factor for a patient to see that they can move their arm some degree.”

“I think motivation is a huge part of rehab. And if you’re not getting any wins on the board then trying to focus on something that they're slightly better at, just to show that they can have a bit of positive change in that area. And then going back to the area.”

**Clinical Experience #4**

“I think error-based learning is where I’m doing movements with the upper limb where we're refining their skills and then making slight errors all the time. So, slight errors might be in their trajectory might be in their distance or their speed. If they're having to say, reach and let go of something, and they let go of it too late, they'll miss the target. So, what's happening is their sensory prediction is not matched up with what actually happens. At an unconscious level they go through a process of learning. That learning is probably happening where they don’t have to think about it. That learning happens through the process of time and repetitions and subtle variations.”

“So, you can do error-based learning, but then, if it doesn't really stick and create any meaning or reward, then I think we can unlearn again. So, they can have made some gains and then fall away again. I think as soon as we get some kind of new skill, with skill-based or error-based learning, we've got to try and give it context and goal-based context as often as we can, because they know as leading somewhere.”

“Feedback is a quite confusing term. When I think about feedback I’m thinking about knowledge of results and knowledge of performance. A lot of the knowledge and performance information would be related to error-based learning. So would be often at an unconscious level. But there is feedback going in. Where they're getting some information there unconsciously and it's changing their movement behaviour. It's giving them enough Information to direct their movement. So, I’ll give you an example. We were looking at that upper limb reaching tasks and often patients will lean forward with their trunk. So, they're not using the new shoulder flexion and they’re not using the new movements that we've given them, shoulder retraction. So, what we did; we get this little Velcro dots and we put them on the back of their shoulder so that when they move away from the chair they start to get this pulling sensation. Now you can just leave that there and then unconsciously it's a like a little cue or reminder to keep their trunk back. So, they're getting a little bit of feedback there, but in the meantime I’m making verbal feedback to get higher or to go faster, something that's a coaching feedback. So, you can use a combination of different kinds of feedback. But then obviously when you're setting the tasks so that it's relatively achievable. Like I said before, with the home exercise program - where they're getting the knowledge of results around number of successful attempts or to reach up to a certain point, or to open their hand and let go of an object certain number of times - then you're getting feedback from more like the actual outcomes. I do think feedback - sometimes when I worked with my physio staff - gets a bit confusing around things like: just queuing people, or some of the other feedback that might be the reward based stuff I think.”

“Some patients do email me back and they say how they're going, and I just try and motivate by saying, ‘I want you to do this amount.’ Sometimes it's time based. So, I think one of the things I learned from some strategies of constant induced movement therapy, if they're doing an exercise say, running for a minute. They may be doing a number of successful attempts within a time span. And that works well, often quite, because it puts a little bit of time pressure, it adds to the level of attention and concentration. But also shows them that they can see that I say, “If you were able to do more over a period of time, then that's really quite exciting,” So, I pump up that that idea that small gains are actually quite meaningful in this in this field.”

“I'm excited by virtual reality, what that might bring to engage people to use their arm more.”

“Reward-based learning would be more about...somehow the brain is getting some kind of dopaminergic hit, if you like, that “that was quite good.” I don't know what that would be, but in my patients, it might be a movement that is suddenly a new one. New and novel that's exciting and it feels good. It may be a movement that's pain free, that was really stiff and painful before. So, that can be a real breakthrough for them and they then sort of play with that movement because it feels very nice. It also then might be that they're able to achieve more within the small moment that what they could before, and then generally getting excited by the fact that they're making progress. That's where obviously we could gamilfy it a little bit. We could gameify with exercise saying, ‘You get to this level, you can level up to the next level.’ The other reward might be they're actually achieving functional tasks now and they’re starting to realize and get excited that they using their arm more in the real world. Not just showing up in front of physio, they're actually using their arm. Or their relatives, or their partner or something is telling them that they're using their arm a lot more. Then they they're actually quite chuffed with themselves. So, there's a level of satisfaction. And sometimes it's showing off too. So, they can show off in front of me, or they can show off in front of somebody else that that would like to show off about their progress that they're making. So, I think all those sorts of things are generally an exciting part of rehab. I think what that does is probably helps cement some of the learning and mechanisms that take place and helps with the carryover effect.

“Saying to someone, ‘That was really, really good.’ yes, that can be feedback. It might be reward but they're not they're not actually the same thing. So positive reinforcement might be related to knowledge of results, but I think a lot of the encouragement stuff is probably on the behavioural reward based spectrum, rather than just feedback.”

“Whether they've got any supports. If they don't have people around them: if they don't have a husband, or a wife, or daughter, or son, or a friend, or someone to work with them. And to motivate them, particularly when they've got mental health, and behavioural, and cognitive challenges... Then it's really going to be quite difficult. So that becomes a factor. I know I said genetics and that’s just what I’m reading at the moment but don’t really feel we know what genes are helpful yet.”

**Clinical Experience #5**

“The education very much links to that self-efficacy part. If the person doesn't know and understand what the impairment is and how to treat it, and what's effective; then they're going to very quickly lose interest. So, I think that is really simple and my background is more in cognitive rehab and the three things we always talk about is sort of impairment level, functional activity level, and education. And we'll sort of, say these three things are as important as each other. and I would say in motor recovery, you might not spend as much time on education but it's as important, because it creates insight and awareness which then leads to self-efficacy of motivation.”

“For patients that maybe are a little bit distractible or their persistence wasn't very good, the faff of getting them into some of the robotics would be a bit of a negative for them. The way we were taught to think is that, “Oh definitely go for just lots of lovely functional tasks and fun stuff in people that that have trouble concentrating.” but actually sometimes the robotics really lock people in, and I think, because they have some of that goal driven sort of games, and things that motivates people.

I think the VR headsets as well, I've seen people who are highly distractible in other situations, and you put robotics plus VR on them and they in the zone and you almost have to pry it off them.”

“A big factor to me is also self-efficacy. So, the person that's doing it needs to both understand what it is that they're trying to achieve...what the elements of what they need to do are; but they also, more importantly, from a self efficacy point of view, need to believe in it.”

**Clinical Experience #6**

“Feedback is important for the patient because it informs the patient when they should stop and reset and retry. So many times because - so like what I earlier said, I am taking a look at a balance between compensation and restitution, right. So when you take a look at restitution what you are working on is you are working on correct movement pattern. Okay, most of these patients with stroke they come in with abnormal movement pattern. Number 1, they may not always realise that it is abnormal. This is how I can move, this is how I move, right. Then you kind of need to correct it. So their feedback is extremely important because that it informs them that they are not quite doing it correctly. It's not a normal movement pattern, and they would know when to stop, reset, and try again. So that feedback is extremely important. So whether it is a feedback of the physical movement using a mirror, or using somebody telling them that they are not quite doing it correctly. Or feeling it in their joint, it feels like my shoulder is a little bit further than my trunk, from my trunk. It should be closer, and stuff like that. So that feedback is, to me is very important because the therapist is not there all the time with the patient. They need to be able to utilise this feedback to make sure that they are actually doing their practices correctly.

“When I say social support, it's not just support in terms of a tender loving care kind of support, but support as in encouragement. Appropriate encouragement. So sometimes the social support of the family can be very positive and encouraging. But then it becomes unrealistic. Or it becomes, or maybe, so a very good example was the patient I saw yesterday. A youngish patient, about [30 years old]. So the father wants the son to be able to do more. But he makes it sound, but he sounds very, like he puts down everything the patient does. Oh, it's not enough, you must do more. So there is no encouragement of the success. He's just pushing and pushing and pushing. So it's social support, but appropriate level. Not too much on one side, and not too much on the other side.”

“Social support is a huge thing, yeah. When I say social support, it's not just support in terms of a tender loving care kind of support, but support as in encouragement. Appropriate encouragement. So sometimes the social support of the family can be very positive and encouraging. But then it becomes unrealistic.”

**Clinical Experience #7**

“Working out the relevance and what’s buying in to the person is really important.”

“I think engaging a person. I think those things are really important. Seeing success, seeing progress. But I think you're really - if you're not motivated you're not going to do it and then you won't get enough practice, enough good experience to help drive the recovery.”

“If they can’t do one arm, the other hand can sort of do an assisted assist. So I’d be looking at ways of trying to get the person to be able to take as much independence and onus on the particular activity as possible. Because I think then - I think that empowers a person.”

**Clinical Experience #8**

" It would be good if you could keep people for as long as they are continuing to improve. I mean it may be that it's actually - I would probably do a bit of sending them off to practice and to consolidate and then perhaps bring them back for another week's burst or whatever.”

“People having access to rehab, not every day, but having access to rehab for as long as they need it. It's a long-term condition, stroke. They shouldn't, you know, get your community, get your outpatients who discharge and that's that. They should be able to tap back in and out.”

“Well, I think it's really important. I'm often amazed by what people are told when they come to clinic. They say oh, I've done this because I was told to do it. What they've been told to do makes absolutely no sense to me and to [redacted] or whoever, but because somebody in, for want of a better expression, because somebody in a white coat or somebody in a therapy uniform has told them to do it, they're doing it. The problem with that is that if anything changes, they still do it because they've been told to do it and so they're not responding to any changes that are happening in terms of their recovery or even in terms of secondary complications or anything because they don't - they're just doing what they're told.”

“If you educate somebody and tell them why you are asking them to do something and what you're hoping will happen and what to do if they start to develop pain and they're able to take that on board, then it's not just a blind trust in the therapist. I think we need to enable our patients to take more responsibility. Not responsibility. To have more ownership of their recovery. I don't think we enable our patients to. We tell them what to do and expect them to do it, you know, here's 50 million exercises, do them every day, but we don't tell them why often and then people end up doing extraordinary things.”

“I think they need to understand what's going on and how they can best help themselves to recover because then if things change, they know better how to respond to that.”

“I think there should be a component of education and coaching so that the patient, if they can, so as far as possible they understand what you're doing and why and therefore how to move their own progression on if they need to.”

“I think it would be motivating for the and the therapist. I think it can be quite soul destroying when you're working away and working away both as patient and therapist and changes aren't occurring.”

**Clinical Experience #9**

“It’s like maybe the most important part because if you don’t provide good education then patients won’t have buy-in. They won’t - it’ll just be kind of meaningless activities that you’re holding someone hostage doing if they don’t know why they’re doing it or the importance of - yeah, they need to know why you’re doing every part of their session. So I think education is important to do that as well as helping to set realistic goals for recovery or an understanding of what to expect from not just your intervention with them but stroke in general.”

Lived Experience Group:

**Lived Experience #1**

“I’ve seen people with similar problems as mine but their wife or husbands have too much pressure, so they leave. So people…no. If you love each other, if this happened to my wife, I would do this for her, she would do this for me, it doesn’t matter. She helped me still be here”

“Yes definitely, that’s what I would do [access rehab whenever I want to]”

**Lived Experience #2**

“You have a therapist there on an ongoing plan that lends hope. Realistically, I still have a dream it's all going to go away.”

**Lived Experience #3**

“Think one of the toughest things is people who live in country areas that just don’t have access to the same resources, either the people with the skills who’ve done it many times before, or access to the equipment”

“It’s very difficult to do it without good family support. So, I’ve had very good family support from my wife and my three children and everybody around me. That’s certainly been a very critical factor in my recovery from the start right until now.”

**Lived Experience #4**

“I’m very lucky to have [my wife] sitting beside me.”

**Lived Experience #5**

“I think it's [feedback] motivating. I also think for me personally, if I get stressed or anxious about something that really affects my ability to use my affected side, my tone, my spasticity will kick in. And I really notice I get a bit better outcomes if I feel like I’m achieving things. I mean if I can see myself achieving things, I'll be more relaxed and the outcomes are better.”

“I think giving constructive criticism or feedback is really important. I think I've got relatively good insight. But it’s really important that therapists give you that. Like encourage you, and when you're doing something right. That really for me, it seems to really cue neuroplasticity. I mean, if someone says, “Yep that’s good, you’re doing well.” I don’t know I just feel like my brain takes that and, yeah. It seems bizarre and I’m not sure if it’s overly scientific but it seems to work. And I don’t know if that's just my personality or not. But yeah, that seems really with helpful.”

“I think funding would be probably be there for sure because I'm covered by TAC, I can afford it, where, I don't know if I could have. Like I’ve seen private funding. You know, I've seen people with, similar issues. Go through public, the public system and have like no where near the outcome that I have had.”

“Lock down has shut the hospital system that I do therapy in several times. Four months at a time. Which means I have gone months without therapy. I guess that comes back to the access that I was talking about earlier. I feel that arm therapy, I have really missed out over that time because I can’t do much of that at home.”

“I think where you live is important. I made sure that I have been able to remain close to my rehab centre so I can get here. I think if you were regional, think that would be a big issue. I have a few friends from survivor groups that I have met and one of them has literally moved to Melbourne so he can be closer to good clinicians. Yeah, geography I think also.”

**Lived Experience #6**

“I’ve been thinking about this. I think that GPs don’t really know too much about what’s going on in the outside world, what’s going on in here because it wasn’t my GP who referred me. I found Dr. [redacted] through a friend, so to speak. So, I think the medical profession needs to know what’s happening in various centres. I’ve had various treatments, but I’ve had to find them through word of mouth. So, if I was referred here back at as the grassroots…I love my doctor. He’s a wonderful man. He’s been very kind and he’s tried very hard, but I think he’s been a bit lost with the stroke, to be honest.

**Lived Experience #8**

“That was a reason that we came back because we felt that we would be able to get the skills and care that I needed. I felt as if in England, I could have done so much more.”

“The ability to come in and out of it is very important. And the fact that this is one country where you can do that, England should be as well, but it's not.”

“Now this is the real glue! [Laughs] I couldn't have done it without him [my husband].”

“Being able to explain also what one is doing and why, that's something very important. I think each time they say to me, “The reason we're doing this exercise is to get that muscle moving, to get this happening.” They keep saying, “building blocks.” One step after another, it's very important for us to know that that all these things individually may not seem like much but there is a method to all this, and the result will be. Or will lead to something. I think I alluded to it earlier that when they showed us how the different muscles work or can be encouraged to work. That was a visual, and very effective demonstration about why we are doing these different things.”

“All the months in England. It was lack of physiotherapy. They just didn't have enough people.”

**Lived Experience #9**

“That'll give him some indication and motivation to what he's doing. And what he wants to do.”

“Got a couple of other people that are helping me out. Slow it down, straighten it up a bit, don’t wind your leg around, Keep steady.”

“But that motivated me to get out of bed in the first place. I'm a fairly flexible because I don't have to wait like you guys. So, I was getting up at 9:00 because I set up my mind to go to the gym at about 9:30 / 10 o'clock. And that kept me...if I didn't have to go to a gym I’ll be in bed until about 10:30. So that shows you the motivation is important, and you got to learn to motivate yourself first. But it's great for people around you to motivate.”

“Motivation is very important to me and that's why I always wanted, perhaps, someone that could come into the room, that could sit around with me.”

“I did meet a couple of people, They had different symptoms to mine. They were learning back to walk again. But you know, that helped me in the certain thing: to get out of bed, go and visit them, sit there and talk to them.”

“I got my wife and the kids to come and visit me from the street. Because they couldn’t come in. There's an area there where you can see them you know? It feels good. It got me out of bed it made me move around.”

**Lived Experience #10**

“My husband is so important for me and, he's really stepped up and taking care of me. When I couldn't speak at all, he was the only person who got what I wanted, and he took the time to figure out what I wanted. Yeah. All the doctors and the therapists, they say take your time, but they are rushing. I knew they were rushing me.”

“I think the motivation. Yeah. So some days I think I don't want to do any therapy, and I don't want to think about it again, so, but not often. But yeah, I think maybe some people have that more and - yeah. I think the motivation is a big factor.”

“When I was in the Rehab, there were so many people who I didn't get along with. So, I think it's good to get a group, but maybe a couple of groups. So, you can find one who - so you can find your place and then you don't have to stay and then go to another one if you like, no.”

### **Subtheme 4B: Content is crucial**

Preclinical Research Group

**Preclinical Research #1**

“They’ll need to have ways in which they can offload the gravitational stress of their upper extremity so that they can engage their hand and their elbow in individuated movements.”

“To engage true neuro-rehabilitative therapy for severe stroke patients requires assistive devices. I think it requires distinctly different reward and reinforcement paradigms and probably a prolonged and distinctly different neuro-rehab environment.”

“If you want to engage whatever restorative circuits that are available in a severe stroke for distal upper limb control, you have to engage them. and if the patient can’t because they can’t move their proximal limb enough to start to meaningfully try to engage the distal limb, that’s a problem. Because you’re just not activating the circuits available. If they had premotor cortex, if motor cortex and descending projections had been damaged by a severe stroke, you can’t get those circuits of distal upper limb control that might take over function, active routinely, in a therapy. You’re not going to engage whatever plasticity is available.”

“For severe deficits, those patients have to have assistive devices the help proximal forelimb control so they can get their distal forelimb in position to start to use it.”

“Plasticity is available in the circuit that you want to recover, and you don’t activate those circuits – say for the forelimb or the...if we transfer to the human: the arm. If you don’t use those circuits, then you don’t engage the plasticity of those circuits.”

“Trying to get patients to achieve the right truncal posture. The right positioning and use of proximal forelimb motor control so that they can essentially not cheat and start to engage compensatory movements. And really focus a lot of the activity on true distal forelimb /upper limb control.”

“We need to activate all of the gait sequences repetitively with training and feedback in an intensive way to get gait back. And so there needs to be a linear strike with heel strike and toe off and appropriate transfer duration. We need to ensure appropriate foot placement and the lower leg and so an isolated therapy that engages strength is going to be ineffective because its really the entire movement sequence that needs to be reinforced and enhanced. And so that is obviously applicable and crosses over to the upper limb as well.”

“They do a look of sort of passive and active limb movement while seated and they’ll do stationary bike and they’ll do those things. And we are constantly walking into the rehab unit and saying no get the patient up and walk them. if they want to regain overground walking, they have to walk. Therapy itself is engaged towards age old practices rather than task specific and intensive activity levels.”

“We would need a wholesale re-engineering of subacute rehab. So, as you know, we don’t really stimulate any meaningful kind of task specific activity in terms of frequency, dose and type in a clinical neuro-rehab setting. As so we would need to fundamentally redo how we would do our subacute rehab.”

“For severe deficits, those patients have to have assistive devices the help proximal forelimb control so they can get their distal forelimb in position to start to use it. So, for example in home-based therapy for our patients who have been discharged from acute inpatients, they’ll need to have ways in which they can offload the gravitational stress of their upper extremity so that they can engage their hand and their elbow in individuated movements. You can’t expect them to sit there at a chair and try and reach. So there has to be a restructuring of some of the activities that they do at home.”

“Now you can use assistive devices: robotics or other things to try and get the upper limb in a position so that they can start to routinely use the distal forearm. We know from clinical trial evidence when we did that we didn’t have, I believe, the right dose. We didn’t do it enough. I also think that ultimately, severe stroke deficits are going to need some kind of pharmacological or cell-based therapy. I just don’t think we are going to break through severe stroke even with assistive devices or robotics and more intensive activity frequency or dosing.”

**Preclinical Research #2**

“We know from animal models, whether it is primates to begin with and then rodents. That high intensity, high dose training of capacities – quality of movements – works. Not focusing on tasks, not focusing on function. Actually wanting the movements to be normal. Now if you do hundreds if not thousands of movements focused on quality and return to normality, in monkeys and in rodents you can get quite startling results.”

“What should one do for impairment reduction? What is the right way to get people to do hours and hours and hours of movement quality capacity training outside of ADLs and tasks. So, you have to create an enriched, enjoyable environment and you have to make people make movements that are non-task based that are training capacity.”

“High dose training on capacity and movement quality work in animal models. We don’t get anywhere near those approaches in humans.”

**Preclinical Research #5**

“Deweighting the limb, it's not magic, it's just reducing the drive to the shoulder. So, reducing the drive through the shoulder - you can do this mechatronically using robotics, but you could also do this electrically. Stimulating the deltoid muscle so that there's less drive necessary to the deltoid to even deal with the weight to the limb. The result will be the same. That's what I mean by understanding basic mechanisms. That basically tells you, what the approaches are that we should even consider. So driving deltoid either by reducing the need to activate it or by activating it through other means would be a way by which one could already reduce the expression of the synergies a great deal, as an example.”

“If you start to make a limb heavier, what we have learned is that you start to not use your contralateral hemisphere, but you start to use more and more the non-lesioned hemisphere. And that makes you stronger, it allows you to lift up more. But it makes you – to use a PT term - less and less functional. Meaning your workspace gets smaller. The ability of opening the hand becomes less. So now you're driving it not cortico-spinally using cross pathways but you drive it ipsilaterally with cortico-reticulospinal pathways, since those are the same pathways that you and I have.”

“Telling them that they're actually having their client do too much at their shoulder, thereby actually promoting the use of the backup system. Which are just not helpful. So, it's a totally different way of doing physical therapy.”

“We’re finding if you actually make things much tougher then you basically get to use these backup pathways. And you may actually make your client much more impaired. Plus, they start to use these pathways and it becomes a new learned behaviour. So, avoiding that early on it's something that we're working on with new interventions that we're doing really within days after a stroke already. To avoid the use of these ipsilateral systems.”

“I can still reach do lots of reaching exercise, but I may need to support you more either using robotics or using electrical stimulation of your deltoid muscle. Whatever means you use, but I need to reduce that drive through the shoulder because that's actually having you as a client, or patient, use backup systems that are just not helpful.”

“Well, the initial deweighting, progressively actually making the limb heavier. So we call it the abduction loading a paradigm. where based on reaching abilities and potentially based on these more quantitative measurements of which side of the brain is active - which is what you brought up. Or how to the connection is from the brain to the muscle. Then use that as a quantitative metric to say, ‘Okay, now we can deal with additional loading without starting to use these ipsilateral systems,’ So we have to incorporate the remaining circuitry more and more over time. And that will be the progression.”

“[manual deweighting] It’s not very effective because how do you know what the weight of a limb it? What weight you need to eliminate exactly, and how do you know what your subject does? That's all very subjective. So, no, I think that that's another thing that we need to change. We need to come up with devices that can do things better than we can do. But our hands, this is an addition! So, I'm not saying, not to work with your hands, don't get me wrong. But we need more objective ways of doing things. That's the only way we can have a precise controlled environment.

Clinical Research Group:

**Clinical Research #2**

“I’m a firm believer that big pharma will pave the way.”

“If somebody blew off their CST and all they have is some corticoreticular spinal tract stuff, possibly driven largely by their contralesional hemisphere, well maybe we should be doing whatever we’ve got to do to activate those brain stem areas.”

“If somebody just has a little bit of shoulder rotation and that’s it, a program might start off with different prosthetics, actuating devices or a sling, all kinds of things, a saebo springy doo-da. But you might need more.”

**Clinical Research #5**

“But just to kind of establish the framework, the things like brainstem drugs, you know, I guess people call them – well, they should think of them as primers. Those are not treatments, those are things that can enhance the effect of behavioural training, but if you give fluoxetine to somebody and expect it to make their walk better or make their arm better without any sort of behavioural training then you haven’t – you know, that person doesn’t know anything about the neuroscience of recovery at all, and those are the people who designed those huge fluoxetine studies by the way, because they were a waste of money, let’s be honest. So, the modality is behavioural. That’s the first thing to say.”

“Then, if we think about some of those brain states as being fluid rather than fixed, so not anatomy but, you know, I guess that’s plasticity, then you can start to think about how you might modulate brain states with brainstem or drugs or enriched environments et cetera et cetera in order to maximise the effect of your behavioural intervention. I think that’s the framework for me, and I think studies should be done within that framework.”

“If they don’t have voluntary motor control of the extensors. But you can also think about new kind of therapies: neuromodulation therapies, maybe we can influence things but until now there is no signal in my opinion. Maybe vagus nerve stimulation might be effective in the very early stages combined with exercise therapy, I don’t know?”

“I don’t think there is any evidence to say that, you know, VR versus robotics versus spending time with a physio - I would rather – you know, if it was me, I would rather spend time with a physio. I don’t think there’s any suggestion that any different sort of behavioural treatment is particularly different.”

“I think there are people who – there are nuanced discussions about the benefit of task-specific training versus more non-directed type training. So, John Krakauer will talk about trying to encourage people to just learn to love moving again, this idea of motor babbling he got from watching babies just babbling with the language, just getting to explore their range of movement. I mean that sounds interesting, but we don’t have any empirical data at the moment to suggest that that’s going to be a huge advance on what we currently do.”

**Clinical Research #7**

“But again, let's be realistic, the patient isn't going to sit and do nothing all day; it wouldn't be the right thing to do anyway. But in an ideal world, if they could have four hours of therapy, which was targeting normal patterns of movement, then that would be what I would want to do with my patients.”

“The problem is that you do that and then you come up with a trial but it doesn't come up with a positive result and everybody says, oh well, robots are not worth using. So I think it's very, very difficult to get the evidence and I just - you know, we're a long way away from doing that, so technologies - I'm quite sure that technologies will increase; the use of technologies, but they - it's slow and they will certainly never take over from therapists. They've got to be something that therapists feel confident using.”

“They [robotics] provide many, many more repetitions than you would get in an everyday way. They provide feedback on success. You can adjust the level of activities so that they are challenging but the patients get the reward of success. They can be used independently; a patient can be set up using a robot and they can carry on - you know, if you think of the Armeo, for instance, or the - any of the upper limb robots, and they can be practising with them without having a therapist there all the time. So they're going to get many, many more repetitions, varied repetitions and repetitions that are at the limit of their performance, and they're getting feedback on their performance. So, I think the theoretical use - argument for using them is very strong.”

“I think that we have a big problem with translating technologies because it isn't just about having the technologies, or even actually having the evidence, which we don't really have yet for technologies. It's also about changing culture and also educating therapists so that therapists see technologies as being one of the - something in their toolbox that they can use and they feel confident using it.”

**Clinical Research #9**

“If you start looking through the recommendations, they’ll talk about things like constraint-induced movement therapy. As you’re a clinician, you realise that it’s a minority of people who actually qualify, if you go through the criteria for constraint-induced movement therapy. One of the others is functional electrical stimulation. There’s like 52 randomised control trials for the upper extremity in functional electrical stimulation or NMES, which, certainly, I’ve seen work. I have seen it, though, applied to a bunch of people with severe stroke, and you don’t see a lot happened.”

Clinical Experience Group

**Clinical Experience #1**

Yeah, so it's funny. Well, it's good for - well, let me back up. I treat spinal cord injury different from a stroke in that way where stroke, the research shows it doesn't matter what the movement looks like, as long as they get their arm moving. It doesn't matter what their gait looks like as long as they're walking. So no, with stroke it depends on what's not working. I can explain it better from a spinal cord injury standpoint where let's say they have thumb flexion, right, which is functional, they can use their finger flexion and their thumb flexion but they have no opposition. Their opposition is very weak or it's not firing or whatever. In that moment I would focus on what's not working in their impairments if that makes sense. I'm going to pick activities that require use of that muscle versus an activity where they would be able to compensate. Maybe I'd pick a much smaller object where they really have to focus on the opposition versus gross grasp in that case, right.

I struggle a lot with stroke I care about quality of movement. I think long term about ortho complications that happen after neuro injuries but research is really heavy on it doesn't matter what it looks like, just get it moving. I have internal conflict all the time on that. I hope I answered your question.

**Clinical Experience #2**

“I think also I think resources are quite important we haven’t really touched on that. I mean obviously we’re talking about conventional therapy because I’m a fan of conventional therapy which is that whole goal based, problem solving, hands on, not hands off. It’s about making patients understand what they need to do. There is something about Tele-Rehab maybe has a window further in the future to give patients some opportunity for practice.”

“We can’t just do task practice. Task practice for good arm and hand recovery: get’s you just to hold an object, or do tasks, but it needs to be meaningful, it has to be part of your participation in your daily routine. You will not do exercises unless it’s got a goal orientation to it, and that you then can incorporate into your daily life.”

“If we don’t do the whole ICF we get stuck at task practice which Cathy Lamb talks about a lot, shoelaces, buttons and zips and eating and knife and fork, that is not using your arm and hand in an occupation-based way, that’s using it only at task and you’re stopping at task. I think if you want true change for a patient to be able to feel independent at those opportunities to use their hands, particularly hand movements, shaping of hands, grasps, grips, lateral grip, pinch grip, that has to be trained, patients do not spontaneously get hand movement and then just its functionally because they might not have used to functionally for six months and they’ve forgotten how to use their hands bimanually.”

**Clinical Experience #3**

“So, robotics, no matter how skilled you as a clinician, to completely deweight someone’s arm is pretty tricky. As well as helping to facilitate some type of movement from them. I'm not saying it's not possible. But I think if a robot can do that part for us, at least in some of the practice. It does allow the therapist...it does allow a severe patient to get in more therapy, with less intensity of therapist giving it to them too.”

“I think it does remove some of the barriers to that patient demonstrating whether they do, or do not have any movement at all. And I think by doing that you can help make a clinical decision about whether they do, or do not have any movement. But also, you can help give the patient some feedback around the fact that there are some messages I suppose getting down to that arm. It's just, at the moment, they're not strong enough to elicit the movement that we need.”

“For me, I do think it has a role to play hands down. And particularly for those severes. I've used it in rehab a lot for those patients who have no movement, and you know, this robot...obviously the robot that we had there could pick up movement that we couldn't see while deweighting the patient's arm. So, they do feel like they have some ability to use their arm. I think we can over rely on technology. I would definitely say that there's sometimes in groups where it becomes, “well let's just set the patient up, get the reps in rather than it being a quality, kind of, tool to use alongside a well-developed rehab program.”

**Clinical Experience #4**

“I would use a physiotherapy session to be like guided discovery to them. Figure out what's the two or three things I want to leave them to do as homework. So, I do find a lot of my upper limb sessions are trying to activate some movements and find some things, and then figure out what works well.”

“It could just be able to hold an object there for a while the other arms doing something. So, that the arm, there's a sense of body ownership. The arm actually belongs. That they move their body in that direction too. So, we see a lot of trunk movements don't go in the direction of the severe side so.

If we get them propping or taps in there on the wall, then we can actually get them moving differently through space we can change the game. Then we start to realize that the upper limb opens up movement options for other functional tasks as well.”

“Most of the interventions, I would say for different categories...One of them would be a lot around priming. So, what I mean by priming is I’m probably trying to improve sensory awareness and body schema but also prime up muscle activity in groups that obviously are not doing very much and I'm trying to get them involved. And so, a lot of intervention for the beginning of a home exercise or beginning of my intervention with the patient will be waking everything up and getting everything fired up.”

“So, I’d probably do that through getting the patient to do certain actions and certain activities. I also might do it through sensory stimulation. So, I might stimulate the skin, or I might brush, or rub, or vibrate, or approximate, or press on areas. Which is probably just helping - I’m imagining - is helping the brain find itself in terms of its body position. So, a lot of it is probably around schema and spatial awareness. And then, when they're actually moving, adding in those extra sensations to give it a bit of extra sensory input paired with their active movement at the same time. So, if they are moving their hand, rather just move it in open space and they are reaching out, actually move out and slide their hand over something so they can feel the friction and they can feel the approximation. And then they may...And then turn priming into the fact that they're doing movements and...even very basic movements in this state and trying to gain control. So it might be guidance around the trajectory of a movement or the distance. So, they have visual feedback, or they have tactile feedback, or they have my feedback through voice or some kind of keyword to help them again begin the process of some kind of control.”

“And these will be movements that have a particular reason behind why I’m doing it. So, it's probably to work towards a specific function which might be propping for sitting, or from lie to sit, or it might be to help press on something to get out of the chair, or use their arm in the parallel bars when they're doing the exercises for their weight shift. Or then start to get functions to bring about being able to reach out and grab something.”

**Clinical Experience #5**

“There is some evidence for some priming. I think the concept of it also makes sense. I think there's a broad church of priming. So, things that can be very upper limb specific, but then also then those things that are more from a...where they've shown you've got more, kind of, excitability in the brain. Or just know say things like people doing some aerobic just beforehand, those kinds of things that. I mean from the reading I've done it kind of makes sense. It's not going to do any harm and I know in specific situations there's beginnings of some evidence for it. If I had to sacrifice something out of all of this it probably would be the priming. But I think if you can put it in and it's easy, and it's one of those things that often can either be done by an unqualified staff member or by the person themselves, I think it's useful. And for the more general things like some cardiovascular beforehand, that has many other benefits as well on stroke health, you know again, it's just not harmful - it's beneficial otherwise.”

“I think there's again there's a role emerging, probably for some of the robotics as well. But again it's out of reach of a lot of services. And it's got a long way to go to be brilliant. But I think there's definitely...there’s certainly built into some of the robotics we are seeing some modules that will actually be able to detect really quite small changes. I have never used anything like EMG clinically, but again I could see how if you're really trying to measure some activity - There might be some value in that as well.”

“And then you need to translate that into function. So, it goes from sort of maybe partial practice of a particular movement to either strengthen or reduce unwanted movements or improving session. Whatever it is. And then you build that up into, instead of that part practice, into maybe a whole task. But then you need to translate it into real tasks that are meaningful. The tasks earlier on...I see problems in both ways I think sometimes people get very focused on everything having to be in a meaningful context - which no absolutely not. Because it's very difficult to you know, to do that with any level of intensity. These particular things like eating or cooking every night - you can't eat for an entire day. You have to do some sort of simulation at some point. But, equally, only during part practice and then somebody will just neglect putting into real life. So, I think that's important.”

**Clinical Experience #6**

“What we're really taking a look at is correcting a normal movement pattern, right. The various steps, or the various modalities or approaches that I use, I order it in my intervention. Exercise and input to the patient to inform them of correct movement pattern, okay. So the assumption here is they have lost the ability to execute that movement pattern. You need to kind of upload that into their brain for them to be able to remember and understand, and be able to execute it in their limb, right.”

“Essentially, they also need to be able to understand how that looks like in real life setting. So that’s the reason why I usually like to end up with a functional task. Because for many of them they, especially the ones who just, I just want to move on with my life.”

“I know that quality of the movement is not good. They are already so used to using compensation in their task, right. They need to be able to see and feel what it is like when they use a normal movement pattern in a functional task, and see how much more efficient and effective it is for them, right. So, a lot of times I like to end with a functional task because that gives them an idea of, ah, when I use the correct normal movement pattern this is what it looks like, this is what it feels like. This is how much more efficient and effective it is.”

**Clinical Experience #7**

“In terms of if someone is moderate to severe, I will probably be looking at some more things like two handed activities. So, things where they're probably more likely to see how they can actually try and get some useful control of their arm for some of the activities. That might be picking up a pillow, might be holding a jar, might be stabilising something. Might be patting their dog, might be threading their arm through a sleeve. So, it’s sort of activities which are a little bit more like simple activities. Rather than fine grip, precision, complex grip, opposition. So, I’d be trying to think about some meaningful activities, classes of action that do work on that.”

“So rather than having these weird anatomical movements, I would put something into a reach and grasp. Or in something - I’d also do a lot of environmental modification for that.”

**Clinical Experience #8**

“The patient needs to be active. They need to be the one doing as much as they're able to. If you're just passively doing stuff to a patient, I'm not sure, you know, that's not going to have the same effect, so they need to be really active and involved in it.”

“You want to incorporate it across the ICF I think, so treating at an impairment level but making sure that then gets put into function and participation.”

“I think there needs to be an element of thinking about the real world, so incorporating what you're doing in a real-world scenario and not just in a fairly - what's the word I'm looking for? A fairly, bland, clinical, yeah, particularly if somebody has got cognitive issues which they might well do. Getting that carryover or apraxia or whatever. Getting that carryover into real world environments is useful, so if you could do a bit within their home and a bit as an outpatient that might be quite good. Access to our kit but also access to what they're doing in their own home or in their local shops, so kind of like an outreach bit.”

**Clinical Experience #9**

“Focussed control and focussed intervention on the arm then and that would be to improve control of that arm which might look like strengthening. It might look like assisted movement, depending on their abilities. Like therapist-assisted or even robotics assisted or FES, like electrical stimulation. Something to really hone in on the activation of that arm that involves that patient’s attempts to activate it alongside. So targeted arm activation. What that looks like depends on how they are presenting but I tend to include both weight-bearing activities like closed chain activities and open chain. Just a - check all the boxes, try every approach. A bit of a throw spaghetti and see what sticks. What works for the person or what they like, I think that’s so important as well. I think those are my two big pieces. It means that I would want to include an aerobic component and then targeted exercise based and I would lean more towards repetition. High repetition as long as it’s not just passive.”

“I think they’re both functional domains of movement control and postural environment control. Open chain is for things like reaching or manipulating objects whereas a closed chain is for weight bearing. Someone’s doing push-ups or wants a stable surface - stability to be derived from that arm. So, it’s not that - it’s a bit of like, well we’re going to do what’s going to - where the patient’s most successful at but also, they’re both functionally important to upper extremity use. Meaningful use of the upper extremity. So that’s why I would include both.”

“If I were to think of a comprehensive intervention for someone after stroke, whether it is focussed only on the arm or in general, I think it needs to include an aerobic component - an aerobic exercise component. Whether it’s they have the ability to reach an aerobic threshold with the use of their arm like a new step versus you might just have to - like if the arm is too non-functional. But I think if you - the aerobic component is to prime the neuroplastic environment of the brain which literature has started to show that kind of link between learning and motor learning and priming - using exercise to prime it for that. Then focussed, targeted movement control of - let’s just stick to the arms since that’s the research study.”

“Oh yeah, for sure. I guess I didn’t put that in my ideal intervention but I would - I like to think of the trunk as your foundation so if you don’t have a strong foundation, you’re not going to have a solid - you’re not going to have a good arm and I often use that example when I provide education to patients as you need a stable - yeah, if you want this arm to be moving freely, it has to have a base of support. For that, it’s the - a solid trunk.”

“It is both quantity and the task-based movement, but I wouldn’t forego quality of what that task-based movement looked like. It’s just a matter of where - what do we prioritise and in some cases, I’ve heard of oh, from this approach, we wouldn’t let someone do this task unless it was a perfectly completed movement. That’s where I draw the line. Like no, we are still going to complete this movement and we’ll work on quality along the way but it’s not a barrier to us pushing someone along in these activities.”

“But the quality of movement is definitely important. It’s not - it’s hard to distinguish them out altogether that as someone is repeating a task, it’s very obvious that this - if the quality is improving or not or what can we do as we do these tasks to improve the quality of these - this movement as we’re going? Then ideally as you’re driving that neuroplastic change and movements, driving the recovery of the brain and ability to turn on specific muscle groups that you’re doing them in a way that is going to improve their movement quality as well.”

Lived Experience Group

**Lived Experience #3**

“I think probably in my case they could’ve done more in terms of the group sessions. Since then, I think there’s been a lot more emphasis on group sessions, which is obviously a good way of providing people with more input without having an extra burden in terms of the staff required.”

“In my case pretty much everything was supervised. If there’d been ways of being able to do it myself in my own time, I think that would’ve been beneficial. Certainly, that only would’ve been applicable very late in the process, I think.”

“Trying different things. I tried acupuncture and various other things to see whether anything else would work. So, I think probably having a variety of different techniques available to try just to see if one works better than the other, I think is quite beneficial.”

**Lived Experience #5**

“If I would have been beforehand, someone that wasn't, and didn't enjoy exercise, I think the gym would be terrifying and you would really struggle.”

“I think that an outpatient hospital setting is fantastic because I would not be able to cope if there was music or too much noise going on. But this is setting is…I find it pretty much perfect for recovery. But I also don't know any different.”

**Lived Experience #7**

“Everyone says, go walk around the house, don't go in your wheelchair. But when you get up to walk, you’ve go to do something. What can you do with the walking stick in one hand and another useless one? No other hand! It doesn't make sense to me. It used to help when I could go for a walk outside. Like there is something. I’m not into just boring walking around the house. but then you've got to have someone with you every day to take you.”

**Lived Experience #8**

“[Groups] become a little bit of a social chats and I found that a waste of time.”

**Lived Experience #9**

“But if it was built into the system and the nurse knew that [redacted] got to get out of bed, you know, three times a day. or four times a day including his rehab. You would have made it a bit easier for me, you know? And even something that I could have in my hands. One is strengthening it, and the other is, you know, repetitive movements that we can do, that i couldn’t do in bed, you know? Things like...if we had a...even a roller that I could actually have in bed and i could actually use it. Something that is more mobile I’m probably more inclined.”

“I’m now looking to get some machines of my own, especially the one that you rotate the arm on the top and the legs at the same time. I used that a little bit at the rehab. But that was only at my own accord. Like, I would be moving from machines, I would say to the physio, “Look I’ll sit here for minutes and then I’ll get down”. So, I’m aiming to introduce that in my arm to...hopefully I can get it moving a little bit easier.”

**Lived Experience #10**

“[Groups should have] about five-ish [people], but it depends if the people are on the same level.”

“So, like writing and using - oh, no, cutlery and that sort of stuff, but - so I guess the functional tasks are more important. Yeah.”

### **Subtheme 4C: Multidimensional dose**

Preclinical Research Group

**Preclinical Research #1**

“We don’t have an answer to that [frequency] at least in subacute. We know it needs to be substantially more activity, but we don’t know what that activity level is.”

“I think there is no one definitive study that shows that but I think the aggregate set of findings is that more is better [dose].”

**Preclinical Research #2**

“We know from animal models, whether it is primates to begin with and then rodents. That high intensity, high dose training of capacities – quality of movements – works.”

“Hundreds if not thousands of movements focused on quality and return to normality, in monkeys and in rodents you can get quite startling results.”

“We know that enriched environments and high intensity, high dose training on capacity and movement quality work in animal models.”

**Preclinical Research #3**

“I certainly believe that dose is a critical factor. I do know that we don't know why dose is a critical factor. I would even believe that for certain treatments a lower dose would be more effective than a higher dose. But, again, this depends on the condition of a patient.”

**Preclinical Research #5**

“Exercise is like a drug, you have to dose it, you have to understand how it works, what exercise do - not just any old exercise.”

“Well, the difference is, if you don't make a limb heavier, you'll never deal with the weight of the limb and more, so it's a functional issue, right? So, if you want to deal with the weight of your limb and maybe get a nice German Weiss beer glass, then you need to deal with a lot more than the weight of your limb. Or a gallon of milk, or something like that, right. So, the point is: how do I recreate the control of a limb that ultimately will result in function. So that's why we need that progression, and we need to do this in a way, where folks do that with remains of their system. So long story short, we even have evidence to show that, if you do this progressively, you actually start using more and more, the remaining circuitry of the lesioned hemisphere.”

“The world that we are up against, you and I, is that when you go to rehabilitation meetings they tell you, ‘more is better, work harder!’ We’re finding if you actually make things much tougher then you basically get to use these backup pathways. And you may actually make your client much more impaired.”

**Preclinical Research #6**

“I think you know that dose is really important. I think if you look at clinical studies, you know, almost all of them, from our perspective would be vastly under dosed.”

“They don't even know if they have the right parameters, yet they just kind of reach a consensus and say, “Oh, this is what everyone else is doing let's use those parameters.” And you know you don't know if those [dose of TMS] are optimal. And then you use these parameters throughout the whole recovery period? Well, the brain is changing over time. So, I'm not sure it makes any sense to keep doing the same thing over and over again. You might have to change those parameters based on what's changing in the brain.”

“That's one thing that we do in our animal studies, you know we...the difficulty. At least in the work that we do, the task difficulty is different, it's not the same throughout a session, you know, there are easy rewards for them to get and then to get more rewards, they have to do more challenging things. So, we can make it progressively more difficult, and I don't know to what extent. I mean, I know that you do that as well on the clinical side, but I don't know how it compares. We might do more of it, and we might let our subjects fail, a bit more than therapists do.”

“In this study the animals had to reach for increasingly more difficult things: extending the limb out and higher and, you know, that sort of thing. And if they weren't able to do that, then it was backed off to the next level down, and then go back up. So, it kind of like a method of limits, you're going to go back and forth like that.”

“Then depending on the patient, they may do too badly and then they'll just get very discouraged, and they won't try. So, then you might have to back off, you know, and go back to an easier level and keep trying that. But I think it's important to challenge them.”

**Preclinical Research #7**

“It seems to be a rule of motor skill learning. That there's a phase where it gets better probably important cognitively dependent, figuring out ‘how’ you want to do it - in that early phase. After that, it wants a lot of repetitions to stamp it in and I don't know why brains do it that way, but they do for motor skill learning.”

**Preclinical Research #8**

“Repetition but considering the - it’s not only repetition. It’s repetition for learning. So I think that we’re identical there but it’s clear in animals that - we’re food restricting them so they’re really motivated and when you’re hungry, a bite of food is really fun to have and also, putting them in an enriched environment is - I think it’s related. It gets them hyper, energised and so that’s the components that are clear. Is that yes, it needs to be learned - repetition based learning but there is these other components that are much more general that are - like an enriched environment.”

“So, to engage reorganisation of the circuit, you need to have a challenge. I mean, that’s scientifically proven evidence.”

“If you just do repetition, it doesn’t do - it doesn’t engage the brain. You already have the circuit to answer that task. You can do it 1000 times if you want. It doesn’t engage anything. It doesn’t engage plasticity mechanism.”

**Preclinical Research #9**

“I think about the nervous system and the role of repetition: in order to drive the system to perform in a different way, and to drive it strong enough way that there's physiological and perhaps even anatomical plasticity in the system to maintain that behaviour over time. The intervention, whatever it is, needs to be very powerful and very salient and so by having -again back to this issue of doing something very easy - there's quite a bit of variability in the way that the animals perform the task, and I think that's one of the reasons it's not effective, is that if you think about driving particular types of sensory motor integration and again, pick your favourite areas that you think are involved in in control of this process, or that you want to ramp up their engagement in that behaviour. If it's easier, and there are few constraints on the way that it's done then the correlation of activity between two areas it's going to be fairly loose and the likelihood that you're going to drive robust physiological or anatomical changes is very low. To really canalize that process in a particular direction that you need a fair amount of intensity, numbers of repetitions, for that to occur and you need it to be relatively restricted in in some in some way.”

“Say you have them do it and they can tolerate it, the effects become less and less. So I think we, we have to do something to continually shock the system in some way - not literal shock with electrical stimulus has it has to remain robust. Intensity does that, maybe that effect of intensity wanes, and so I think that part of having an effective rehabilitation strategy, that can work over a long enough period of time to really change the system in a fundamental way in terms of its physiology and anatomy, doing the same thing, no matter how intensive it is, may not do it. This is really almost philosophical what I'm talking about right? But it's something that I've been thinking about in terms of intensity and saliency of approaches to rehabilitation.”

“I think that task difficulty actually enters into this because, at least with the animals, if the task is too easy, we know that you can have of an animal doing the same task, where they're reaching into a bowl rather than a narrow well that's twice the size of their fingers and the difficulty of that task is very different. When the animals are engaged in the easy task they do seem to - if they're hungry they're not going to lose interest – so they’ll continue to eat but it's interesting. Not only do you do not see changes in the nervous system with those easy tasks, but with the more difficult tasks they really attend to it so much more and that's kind of an anthropomorphic thing to say, but just observing, they’re intensely focusing their vision on the task, more so than with the easy task where they're really doing it in an automated way. I think there's a motivating factor involved with tasks are inherently difficult. If they're too difficult then they just quit, of course. For many, many years we've thought that the most effective therapy would be therapy that's really on the edge of what individual can do.”

Clinical Research Group

**Clinical Research #1**

“I spent a lot of years saying dose was important and then studying dose but then when we actually evaluated it, there is not really any difference at least for the upper limb later, after stroke, with different doses. The data from earlier after stroke, I think the jury is still out. There’s a mixed bag of later phase trials so I think it’s hard to know.”

“I think probably what’s more important than the dose that they get while they’re in the therapy session is ways to get them to do it when they’re not in the therapy session, so it’s incorporated in what they’re doing every day. So, it becomes a motor habit because that’s what we failed to do, right?”

“I would absolutely make it [challenge] progressive or I guess regressive, too, if we needed it. Hopefully we don’t need that too often but that’s certainly we’ve seen that before. So yeah, so the challenge - in order to get someone to learn and improve over time, then we can’t have a fixed point and we probably can’t have the same point for everyone, right?”

“We need a point that’s based on a certain set of parameters or rules that then can be applied so that sets the threshold for the challenge for each person for each task. Then we would have another set of rules that says this is when we can progress because you’re doing it well enough and this is when we regress because you’re clearly struggling.”

“Every individual has their own kind of place where they’ll get frustrated, and they won’t go on any more and so I - we hesitated to set it at a fixed percentage of fail or success but how long it took them to complete a repetition of the task was part of the picture.”

“So, if someone had difficulty opening their fingers, then we might progress the exercise by giving them a bigger object versus somebody else who maybe didn’t have trouble opening their fingers but was trying - didn’t have good grip strength. Maybe they were a little more moderate, right? So then we might change the task by giving them a heavier object that they had to move.”

**Clinical Research #2**

“I think about why that is, I think about this list of five things. Again, what should go into good motor therapy? Well, we asked ourselves that and this is what went into it. We think it’s a pretty good therapy. For the motor system it’s high intensity movement practice.”

“We’re all familiar with a series of studies 10 years ago where they counted the number of repetitions during a therapy session. There were 32. But the animal studies tell us you need to do 700, 800, 900 repetitions to really induce plasticity. So one of the core things is whatever you’re going to try to improve, you have to practice it. You have to practice it intensely day after day.”

**Clinical Research #3**

“I think that the one big one is very intense and high-dose therapy, whatever it is. I’m not sure that content matters as much.”

“I don’t mean just dose, but I mean just this intensity and structure of the intervention is really important seems to be the data that are coming out.”

“The dose is really just counting repetitions, right, and people have played that game, and it doesn’t always seem to yield the same, or yield the good result. So that’s where I think intensity becomes really important. So, you have a task you’re trying to work at, you’re trying to work at it hard, and maybe doing it five times really well, or five times really hard, is equal to – I don’t know what – 25 times just throughout repetition.”

“There does seem to be something about a mass dose of intensive rehab, and this is, you know Nick Ward’s stuff coming out of University College of London, right, where they’re looking at these intense three or six-hour bouts of therapy in a day, five days a week, and that’s a mass dose of practice. That seems to stimulate a very significant amount of change in those individuals. It doesn’t seem to quite matter, like he didn’t even report what they’re doing – they’re just doing that amount. That’s why I’m not sure that the content is super important.”

“it’s like going to training camp, right, and you just pound it out.”

“So, we want them to be challenged, but not challenged – challenged so that it’s hard, but not challenged so that they can’t succeed.

“I think that what we need to calculate is people who are hitting kind of an optimal learning rated change, which we’ve been able to define, and that’s a place where they’re not changing too quickly because the task is hard. It doesn’t matter what the task is.”

“But what seems to be important, for both optimising change in behaviour and also optimising change in neuroplasticity, is to find kind of that optimal challenge point where the task is quite challenging but manageable. You can do these calculations fairly easily and determine that optimal difficulty; as the person gets better at the task, they’re going to speed up, their rate’s going to increase, and that means you have to adjust the task and make it harder again.”

“A gaming rehab intervention for the arm, so it’s a game. So built into that is this difficulty. So as people start to get better at the game, the task increases its difficulty. As they struggle or they learn early, it decreases their difficulty, so it’s always kind of adjusting this, right?”

**Clinical Research #4**

“If we go back to Bernstein, we know that his famous repetition is not repetition. Repetition is - I forget what the exact - you know, repetition is repetition without repetition, something like that. So, when you - if you look carefully, you might think you're repeating the movement, but it's not the same every time. It's that small variation that is important for the system to be able to control, because it also means that there's some amount of flexibility. It's not stereotypical, you know, same thing. So, the term repetition has to be nuanced.”

“We need to think about progression, right? So if you give somebody difficult exercises, they're going to lose motivation because they're not going to be - it's going to be a struggle. So I think you've got to modulate between easy and hard, but you've got to target the things that you've got to work at. What I would call the challenge point.”

**Clinical Research #7**

“So a lot of studies have looked at the effect of amount of therapy and one of the things that we found in that was quite clearly that a small difference in amount of therapy made absolutely no difference at all, but the studies where there was a big difference between the treatment in control groups, we looked - just looked at RCTs, then they did make - it did have a significant effect at all levels at impairment and at activity level. So, I mean, that's I think quite good evidence that amount of therapy, if it's a big difference, is important. So - but that's just the tip of the iceberg.”

“Because - partly because of effort that if you - and again, this is from motor - very basic motor learning literature that, in order - and there are loads of papers from donkeys years ago that show simply repeating the same movement over and over again is not effective in motor learning. To get effective motor learning, you need to be working at the limit of your performance, it needs to be varied and you need to get feedback on your performance. I think that's textbook motor learning literature.”

**Clinical Research #9**

“It just takes huge volumes of intervention. So, it’s like a professional marathoner trying to shave 60 seconds or 90 seconds off their marathon time. The volume of training and the intensity of training has got to be massive in order for them to do that.”

Clinical Experience Group

**Clinical Experience #1**

“I mean I think 90 minutes would be awesome a day.”

“Just that it is evidence based, right, we know that repetition is important.”

**Clinical Experience #2**

“What’s also really important for recovery of arm and hand is intensity and dose. So, intensity and dose can be done in the acute phase if you have that opportunity, but again that’s quite hard.”

“We have to somehow get into giving opportunity for intensity and dose. If you can’t do that in the acute phase, then truly that’s one of the reasons why we set up the Intensive Upper Limb Program. Both [redacted] and I were fed up of just doing 45 minutes of therapy, it just doesn’t work, some patients require high dose, high intensity, they’re able to maintain it, yes if we could get people a bit earlier that would be helpful, but sometimes resources stop us doing that due to waiting lists, but the Intensive Program that to us has felt where we have had opportunity.”

“We have had that opportunity, and therefore we can bring people in and give 90 hours. We did a lot of discussions in the early phase about, what is good arm and hand recovery, do you do three weeks, do you do four weeks, do you do five weeks, do you three hours a day, and we debated that a lot because the literature is a little bit mixed.”

“90 hours are dose and intensity they’re two different things. I wondered whether I think you’d still get outcomes of same even if you did 90 hours over five weeks because I think it was a pragmatic 90 hours.”

“Is there an opportunity there to do four weeks because they live nearer and they come in a morning and do every morning for four weeks, I think the outcome would be the same personally.”

**Clinical Experience #3**

“And then dose wise, I mean ideally. You know, I would do as much as possible with them.”

“Someone with mild to moderate impairment, who had at least some movement and could engage in a decent amount of rehab. I mean I don't think we know for sure what the best dose is. and I don't... I mean guidelines tell us three hours of, you know, all disciplines. Two hours of active practice, three hours across all disciplines. But if you just look at the upper limb particularly even people with mild to moderate, I don't think they can tolerate more than like 30 minutes on task at once early after stroke, I think they do better and shorter more frequent sessions across the day, particularly early.”

“I recon I can do two hours with them on task. Maybe a break at the hour point, very easily. I mean, I think the nuance of that: if they find it really hard to concentrate or they've got significant cognitive and communication impairments that you're battling at same time. I think that comes into it. But I think I could easily do, you know, two hours on task with a patient in that chronic population without it being too much of an issue.”

“You know, being repetitive like that, repetitive task practice aspect kind of talks to the dose part of it.”

**Clinical Experience #4**

“I would say the dose side of things I need them to be able to do enough. So, we're getting a dose in terms of things like strength and conditioning. And also dose in the fact that they're getting enough relatively successful attempts at something to go through some kind of learning process. It needs to be dosed enough that I think they can do they can go away and relatively successfully and independently, or with minimal help with family or carers, do something they are engaged in.”

“So, that might be being able to do tasks that last for at least, for you know, 5 to 10 or 15 minutes. But then, also within that time do probably, maybe hundreds of repetitions of different movements that have some relative form of success.”

“I suppose, with some of those patients who actually have some of those kinds of recovery, research will show if we push them hard, they can recover even more.”

**Clinical Experience #5**

“Why so many repetitions? I mean it's if it's strengthening, you just have to you have to do the hard work.”

“I think using the numbers as targets within the session really helps people to up the numbers, but I don't know. I find it hard to give it a total number over time. But it feels like it's lots.”

“Amount of therapy each day: that's probably really intensity. But it's also a duration over time and I think the reason why that's important is that we see people recover over a really long period of time.”

“And for me, that is when we should still be really hammering everything we've got. So, whatever it is that you feel is the appropriate intervention. Whether it's lots and lots of facilitated, de-weighted reach. Whether it's some FES, whatever it is. Whatever you think is the right intervention then you really keep hammering that.”

“Once you're getting this particular movement... once you are achieving this consistently, then this is how you grade it so you get to the next level or you get to the next movement. or whatever it is. So, if we can skill people up to do that as part of an intervention, I think that's really important as well.”

“There is an element around quality of the subcomponents, I think. And again, I sit in the middle of this: I'm not at the purest end where I think every single repetition has to be perfect and beautiful. But equally I also think that there's a risk that if the movement isn't at least at the right level of challenge where it's relatively close to what you're trying to achieve that you can just reinforce compensation movements. Particularly in reach, even in dexterity.”

**Clinical Experience #6**

“How much the patient can tolerate. Okay, so we are talking about the sky is the limit, right? So, we're not talking about a one-hour session. Because typically a session is one hour. So, we're not talking about limiting it to one hour, right. So, then I would say that it really is patient dependent. Because some patients can tolerate and some patients can't, right.”

“So, if resources are not limited, and the resources I’m talking about is my resources, and patients resources. So, patients energy, and patients how tired, fatigued and stuff like that. So, if we're talking about unlimited resources on both sides, on therapist and patient side. Then it can be for as long as the patient is able to tolerate for me to get to that particular goal for that session.”

“It is essentially about the quality end point, yeah. But to get to that quality time is needed. You know that time is needed, you cannot rush something to get to that quality. You know where I'm coming from?”

**Clinical Experience #7**

“Dosage and experience. I would say - generally I would say little and often, spread across the day. Trying to find places that people can regularly put practice into action. So rather than just a big dose of practice once, I would be looking at little often spread.”

“But I don’t know whether repetition - the number is important - but it’s like amount of practice. So yeah, like I think we - numbers are easier to measure for some things. But when you go, what’s one sit to stand versus one ankle dorsiflexion? Is that equivalent? It’s not really equivalent.”

“Like you've got 10 minutes, let’s do 10 minutes now. So, I’d be trying to find keep - doing as much as you can in that 10 minutes. You're going to need a little rest or you swap and change. I would either have rest or swap to another activity. I’d do those sort of things. But trying to look at some repetition as a volume, probably in the measure of a time would be where I would probably talk about that to people.”

“So, you're constraining degrees of freedom to make it easier for the person. So, it’s trying to find the right Goldilocks' level for the person to start working in, yeah.”

**Clinical Experience #8**

“I think dose and appropriate intensity for the individual. I mean we've done what we've done because that's what was feasible and practical for us, but it may be that you could also have a program that's twice as long and slightly less intense but the same dose that has the same outcomes. We don't know. Dose and intensity being different things, if you know what I mean.”

“The only thing I was just thinking about is the time scale. I guess the biggest thing across the world is that of course you are restricted by what you can do by how long you can keep people on your books for and that's usually limited to three weeks, six weeks, eight weeks or whatever and people don't follow a timeline.”

**Clinical Experience #9**

“Higher number of repetitions than previous in order to drive that recovery.”

“Yeah, it would be coming from that animal research that says thousands of repetitions showed that rats can better use their arms in pressing levers and whatnot. So, repetition is - striving for repetition to mimic or emulate those animal studies would be - and I mean, I guess it’s like if you don’t practice this over and over again, even in the other concepts and contexts, like the Malcolm Gladwell 10,000 hours concept. Why not apply that here as well? Beyond just the literature showing that but just in general if more practice the better someone gets at anything, same principle.”

“I think the importance of challenges is very key to the clinical intervention. If something’s too easy, it might not be very - as beneficial as something that’s challenging but the patient can experience success. Then if something’s too difficult, that’s also not very helpful. It’s like the optimisation curve of where - a training curve. I think that’s also very important. So whether - what that challenge looks like, I think is up to the discretion of the therapist but I think it’s very important to include that in the conversation of repetitions that needs to be repetition but at an adequate level of challenge. It’s not too easy, not too hard.”

“If it’s too challenging. I think that applies to any sort of learning, not just motor learning in stroke but in general. If you want improvement, it’s not going to come from something that’s impossible and it’s not going to come from something that you can do already.”

Lived Experience Group

**Lived Experience #3**

“Yeah, the more the better. Certainly, when I was in hospital, initially I was getting one hour of physio a day, they did step that up to two hours for quite a lot of it. Overall, I think in hospital you spend so much time just sitting in your room.”

**Lived Experience #5**

“In hindsight I think I was probably only getting a half an hour a day as an inpatient. I wouldn’t have even been that as an inpatient. And I wish I would have had more therapy then… but this was the thing, cognitively and fatigue wise there's no way I would have managed that early on.”

“In a burst. Like if it was set up for like, you know, a two weeks burst of or something, I definitely be up for that I reckon, that would be really good.”

**Lived Experience #7**

“You could do it longer, I think to get the benefit.”

“The best progress I have had is when I’ve been in there for 2 week at time and worked really hard doing like a couple of hours a day.”

“Because it’s every day, I think that helps. I do it twice a day and it's the therapy regulator. So, the brain is training itself with repetition, repetition, repetition. Now when I get home, I doesn't do that. So, the brain just goes back.”

**Lived Experience #8**

“I am the sort that would keep doing as much as I can until it happens. I would say every other day. So that there is the opportunity to build on what one did in the earlier session.”

“A minimum of one hour because that's really fast... four hours would be hard going. Depends how difficult the exercise is. I’d have to say one would have to try it.”

“I thought it seemed pretty futile [when exercise was too hard]. I mean, I would prefer to have had very specific exercises which you can actually see and gauge a response.”

“I suppose it would depend on what that movement means to the individual. If it was something that was particularly useful. And that I could see how it would improve my everyday experience then that would be fine.”

**Lived Experience #9**

“Repetitive movement, I'm expecting that to slowly, you know, get a little bit lighter and maybe a little bit easier to have better extension on it.”

“I think influencing the hand is probably just repetitive work. More repetitive work. The hours we get at the rehab area, regardless if its OT or the physio therapy area. I would say that you can do a lot more things in bed.”

“But you know, things that I was wasting...I was wasting time. Even with my outpatients, I got to OT and they would indicate, “yeah well I get your hand sensing starting to work.” But I already know what I’ve got to do, I could do that at home. I could get a lot of, you know, things that once I do one or two of the OPs I didn’t have to come in for an hour and waste it.”

**Lived Experience #10**

“I have a lot of free time, so I am just doing not much in therapy. So I would like every day ideally and weekend off. One to two hours…but I could get bored with it and [laughs] I could get tired with my hand, and - yeah.”

“But then if I was doing two hours with a therapist is too much. [Laughs] I don't want to see them that often.”

### **Subtheme 4D: Therapist sway.**

Clinical Research Group

**Clinical Research #2**

“You have to have supervision by a professional. I don’t - very few are the patients that you can send off in the desert with the perfect recovery kit and they’ll back recovered. Humans need a lot of supervision.”

“I hope I wouldn’t get in hot water but to me a lot of what OT and PT are about is psychology. They have to know how to motivate people and how to keep them in the game and keep their mood up, get over the bumps and get over the frustration, develop resilience, confidence, on top of the neurology, neuroscience insight.”

“Your licenced physical therapist is more skilled than the random high school student. For me that is encompassed in what I mean by supervision. By supervision, it’s not just I sit next to you or I watch you by Zoom and tell you when to lift your elbow up more. But also at the end I see what you like and don’t like, what you prefer and don’t prefer, what works and what doesn’t work. I modify forthcoming treatment sessions based on that supervision.”

**Clinical Research #3**

“There’re therapists I’d send to mum to and ones I wouldn’t, right. So, I think that is that.”

**Clinical Research #5**

“The skill of the clinician unfortunately is important and it’s important because of the diagnostics. A good clinician will go, okay, why does this – and the ward round that we do now, I try and prod the therapist at the end of their description to say, okay well, why does his arm not work, why does her arm not work, as a kind of summary point, because if you can summarise why the person’s arm doesn’t work, that gives you your therapeutic targets, right. I mean that’s just clinical reasoning, and in a way, I’m sure they think I’m a bit odd because they’ve been doing this, you know, this is how they were trained. It's like, well why is this guy – yeah, anyway. But I think it’s getting into that way of thinking. So, the skill of the therapist does matter.”

**Clinical Research #7**

“This is just blindingly obvious really, that therapy is not like giving somebody a drug, not like prescribing a particular medication. It's much more like education; so it's much more to do with the relationship between the therapist and the patient and again, we come to motivation again; the therapist being able to support and encourage and know how to motivate and guide and sometimes cajole a patient into working hard at what their - at their recovery. So I think there's that aspect about it; the educator aspect of it.”

“I think there are things like the actual skills that - the one-to-one skills that are rather ignored now, - terribly difficult to measure but if you look, the skilled therapist handling a patient, guiding them, supporting them, facilitating them, they - what they can achieve is quite remarkable. But it has never been demonstrated in clinical trials because it's so difficult to quantify but - and because of that, I think it tends to get ignored, but a skilled therapist working with a patient is something which you can recognise, even though you actually can't put figures - put data on it.”

“A newer physiotherapist should have had to have demonstrate a higher level of training before they can have a senior position as a senior clinician. We need to put pressure on the health service to - not to employ people who have not done high level training, in order to make sure that high level training can be done. So, to put pressure on employers to enable the therapist to do a higher level training.”

**Clinical Research #9**

“A good therapist or clinician will sit and explain, well, if we can get you to do this squat to sit to stand, that’s a preparatory exercise to begin gait, right? That’s not a good upper extremity example.”

“you’ve either got a good therapist who can quickly and intuitively see things and change the pattern as you go to adjust your therapy, or maybe you don’t. They’re going to slap that FES on you for the next 10 weeks and hope that it works, because there’s 50 randomised trials showing that it should work, right? I see that. Both ends of the spectrum, right?”

“It’s [therapeutic alliance] important for a few reasons, right? A bit of it gets back to some of those psychosocial things like engagement, right? So, if you have a patient who’s motivated, and you have a therapist who can encourage that motivation, so, they come down and they do the work that they need to do to rewire their brain, then you’ve got a good combination. If you do not have a good therapeutic alliance between that therapist and that patient, that patient’s not going to have as good a recovery as the person who does a good therapeutic alliance. So, you turn off, stop showing up for therapy if it’s outpatients, like, they just - patients just won’t go, essentially. When they’re inpatients, they will still go, but they don’t participate as well. They fight the therapist tooth and nail on everything they do.”

“When I talk about a good therapist, that’s part of it, being able to create that therapeutic alliance so you can move things forward and being able to do that with all sorts of different walks of life. Maybe part of it is, if you’re on a service where you have multiple therapists that you work with and you recognise that this guy or this gal - you’re not going to be able to achieve that, you just cannot get in their mindset to be able to push things forward, step aside, let one of your colleagues take over, right?”

“I think the back end is the hands-on piece. I think we’ve all seen it. There are some therapists that I work with or have worked with who are just remarkably good with their hands. You give them a patient, you can pretty much guarantee that that patient is going to make improvements. They understand visually, biomechanically, movement patterns, they can cue the patient well to make changes to those patterns, and my assumption is the brain rewires because they’re watching every step of the way and they’re doing what we talked about - they’re modifying their treatment strategy as they go. They pretty quickly see something’s not working, this patient’s not improving, and doing a thousand more repetitions of this is only going to lead to frustration, so we need to change things up. And they keep the patient engaged. Therapists who struggle often aren’t as creative. They have trouble with therapeutic alliance. They can’t make those on-the-fly adjustments. They’re not so good at using their eyes and their hands to figure out that they need to change something. So, not saying there’s bad therapists, I’m just saying there’s a range of abilities in the therapists that I’ve worked with and seen over the years.”

Clinical Experience Group

**Clinical Experience #1**

“Rapport building is something I work hard to do. I think trust is really important. Yeah, it usually takes me like maybe one or two, three, depending on the type of patient, it can take you five sessions before you've built this really good rapport. They know what to expect of you, they trust you, yeah.”

“I think the same goes for any profession, we all have received different education, different experiences in life that help shape who we are and not all of us are the same. I think, yeah, the skill, it's important.”

**Clinical Experience #2**

“I don’t think junior staff, or even some senior therapists are able to assess and treat the arm anymore.”

“Your education could be all right but you're still a doctor if people are still coming to you for the cases that they need to, right. The same thing with, I think about this a lot, with backpain. I think there are definitely therapists who are way more skilled and have way more experience in treating that and it's just I think it can be frustrating too because you have a bad experience and you're not going to go back to anyone. In most cases, yeah, therapy is very important.”

“The only thing I’d like to say is I think who’s the right person to do that, is that qualified staff versus rehab assistants. Is there opportunity and a lot of rehabilitation and training.”

“I think we see people in clinic and I’m getting more and more grumpy in my old age and it’s just basic stuff and I don’t think junior staff, or even some senior therapists are able to assess and treat the arm anymore.”

**Clinical Experience #4**

“Then often none of those professions, but especially young professionals, have a lot of confidence for training in how to move the shoulder or begin the beginnings of very basic movements for those with quite severe impairments. There's a lot of fear around damaging a shoulder structures. There's a lot of concern around people who have pain and sensitivities and I think people just then don't touch it. So, I think confidence levels are low.”

“Less is more sometimes. So, giving patients, maybe three exercises that involve the upper limb. And one's easy one slightly harder, one is really hard. And really explain to them, to build up their dose around the other factors that affects them. If weakness is huge, we find we do a lot of strength and conditioning with our stroke upper limbs. Even when there is spasticity present. There used to be a background of fear around strengthening and spasticity, but I think that's clear. Now people are understanding that relationship. It’s a little bit clearer. All my therapists understand it.”

**Clinical Experience #5**

“I think we look for change from a thorough assessment. You're looking for any activity that you can palpate. Obviously, you need to be a certain skill of clinician to be able to disentangle from abnormal tone or associated reactions - If you’re looking at actual activity. And that can be quite tricky. I think the other thing is time, I just think some people tend to give up very early on.”

“And what I will often see is: if you've got a more junior therapists, they will not...they will miss that. So, from they're handling, they will just go through and kind of a fairly standard sort of asking people to do things. And they will actually miss that. If you have someone who's really skilled at handling. Actually, no look, there is a little bit isolated movement here distally in the thumb that is controllable. Or actually, no there's a few flickers around the shoulder and that gives us something to kind of work with. So, for me, I think it's around skilled assessment.”

“The other thing is, what can we do so that then the person is able to continue on outside of supervised interventions. I think that's really, really important and to me. A lot of that's to do with the cleverness of what you come up with.”

**Clinical Experience #6**

“Also, I think clinicians willingness and flexibility and creativity to try and work on the goals that the patient has.”

**Clinical Experience #7**

“In the skill level, too maybe we over complicate things, and we make it unachievable and that sort of empowers us. Because we think we’re so fantastic. We forget that the person's got to actually take away what we’re doing and do it. I think some of our approaches too - with this group of people there is a challenge to try and get them to do practice which is meaningful. The practice which is meaningful that is not dependent on the therapist. Then you have to get someone to actually hold your arm and then you need a high level of skill.”

**Clinical Experience #8**

“I think you need - there needs to be someone that is skilled in assessing the impairments and trying to come up with a hypothesis as to why somebody is moving the way they're moving so that you can treat it. There needs to be skilled clinicians in my opinion.”

“I think access to therapy and good therapy personally.”

“I think skilled therapy is important because I think if you can't work out why somebody has got something then it's very difficult to know how to manage it.”

**Clinical Experience #9**

“But I think, you know, as humans and living through life, we kind of all have a general sense of what movement should look and feel like. We can bring our own personal experience, what we’ve observed of others, and I think we teach students to be keen observers in general. So hopefully they’re not just doing that at their clinic or hospital but with every interaction, I think - at least for me, I watch people move no matter what now and maybe I always did but now it’s engrained in my training so I can’t not do that when I watch someone walk or watch someone perform a task.”

Lived Experience Group

**Lived Experience #2**

“It just not is clear to me where the definition, delineation between OT and physiotherapy is, and that sometimes gets blurred. I won’t say contradictory, but it’s confusing sometimes.”

“I tend to rely on the professional judgement rather than trying to make my own judgement. I’m not usurping the role of the professional. So, all I want from my professional viewpoint is what’s realistic, realistic hope based on what’s happening, and how are we going to go about that. I’m not asking to make judgements.”

“I’ve had multiple therapists, and despite that, over a year and a half it’s declined. That’s particularly discouraging because after the first, I’d say six months to a year, I believe I could do more than I can do now. The arm reach diminished in the past six months or a year, just that the range of movement and the dexterity. That’s my perception. So, that’s disappointing.”

**Lived Experience #3**

“When I was in Singapore my wife followed up whether we could have rehab in Singapore rather than coming back to Melbourne. Even though the acute care in Singapore was very good, the rehab care was actually fairly poor. You’d pretty much be just staying in bed all day and getting just an occasional little bit of physio or other assistance. So, it’s certainly the availability of the skilled people to do it is a critical factor.”

“Who the therapists are is important and obviously depending on their skill level. I mean, somebody like [redacted] has got enormous skill in that area. I think with her ability to take me down the right path and get me back to where I am now, so yes certainly, the skills of the therapists, I think, are very critical to the whole process.”

“Even though the acute care in Singapore was very good, the rehab care was actually fairly poor. You’d pretty much be just staying in bed all day and getting just an occasional little bit of physio or other assistance. So, it’s certainly the availability of the skilled people to do it is a critical factor.”

**Lived Experience #5**

“All my senior therapists have been amazing…I think the junior therapists work really well when you’ve had a senior therapist and I’m aware of what I should be doing. And I’ve already got some training if you want to think about it that way. Then I can use my time well with someone junior.”

“We're retraining a brain here. I think they just are well aware of how to how to push neuroplasticity and really drive that home. It really becomes really challenging, what movement, a lot of more difficult tasks and so on: that’s senior. I think it's a bit more rudimentary with someone more junior, someone that doesn't have that experience.”

**Lived Experience #6**

“My good therapists are here, and that’s why after so many years I’m seeing improvements. Because when I first got unwell, I was told my improvements will be up to three months, after that it’s gone. It’s been 13 years and I’m back here and I’m having improvements. I see it. The things I can do with my walking, up and down the stairs, I couldn’t walk up and down the stairs, but I can do that now.”

“I told them that I love my physios. They got such a reaction. I think they were surprised. I felt like – you develop a connection with people because they’re good people. I said to them, I don’t think of my physio as just physio, I said, I think of it as therapy, and they all laughed. It’s a positive.”

“I’m looking forward to that because, as I said, I have faith in the people here. So, I think how my leg has improved, I can see it coming in my arm. It’s just in the past I haven’t had – I don’t think I’ve had as good therapists as I’ve had here, to be honest.”

“I just don’t feel I had that connection. Sometimes exercises can be just – once with my leg, I was over, and she put my leg in some sort of contraption and told me to move it [laughs]. I couldn’t move it because I had no build-up to it. I had no strengthening, just nothing. You can’t just do something if it’s not working. If your car needs a tune-up, you need to tune it up [laughs]. When it needs petrol, it needs petrol, otherwise it’s not going to go. That’s the way I look at things anyway. So, we need to get it going.”

“I think therapists were coming to her home and I think she had a good relationship with them too. She must’ve felt comfortable with them as well. I remember my mum tying up a bag once and I was thinking, I wish I could do that. She passed from a stroke, so it’s in the family.”

**Lived Experience #7**

“Everyone else I’ve ever had, they get me to do stupid things and when I have asked can I go on the cross trainer. Not that. I hate the cross trainer. But you know, he lets me try things that other people didn’t attempt. Which did give hope.”

“He’s [physio] not like a doctor. He’s actually funny. So, gives you a bit of a laugh and you can give it back to him.”

**Lived Experience #8**

“I think it's the glue to it all. I think one has confidence in their ability to heal, and to help us progress or me progress, in particular. There are things that one can do which they make seem possible.”

**Lived Experience #9**

“Sometimes you need to feel wanted. Not just another injury, you know? Not just another person coming through the pipeline. And when you get that sense of being wanted, and I met up with [redacted] as well, I was talking with [redacted] as well, as you know. I'm here, I maybe, I can access some of my knowledge - different things that doesn't have to be the knowledge on what happened to me, but I think it's great to create the bond first.”

“The bond to me anything I do is critical. Forget health wise, because I was always quite healthy.

But if you look around me, that’s hence why it was such a big support team around me. So, it is for me. it's definitely a big plus.”

**Lived Experience #10**

“The exercise they prescribe, I wouldn't know how to do them and what I am looking for. So it's very helpful. Yeah.”

“I think it's half the personality. I'm spending so much time with them. So if we are not gelling, it's - yeah, it makes it hard. But yes, on the other hand, if with the hand stuff and they are doing the same sort of stuff, I get bored. So even if it's the same goal to go to, the same stuff is boring. So I need to mix it up, so yeah.”
