# Supplementary material for: Considerations for developing complex post-stroke upper limb behavioural interventions: An international qualitative study
Source: Clin Rehabil. 2024 Jul 25;38(9):1249–63. doi: 10.1177/02692155241265271 (PMC11487871; doi:10.1177/02692155241265271)
Supplement: sj-docx-8-cre-10.1177_02692155241265271 - Supplemental material for Considerations for developing complex post-stroke upper limb behavioural interventions: An international qualitative study [file sj-docx-8-cre-10.1177_02692155241265271.docx]

| **Domain 1: Research team and reflexivity**  Personal Characteristics |  |
| --- | --- |
| 1. Interviewer/facilitator | Methods: Research team and reflexivity  Paragraph 1 |
| 1. Credentials | Methods: Research team and reflexivity  Paragraph 1 |
| 1. Occupation | Methods: Research team and reflexivity  Paragraph 1 |
| 1. Gender | Methods: Research team and reflexivity  Paragraph 1 |
| 1. Experience and training | Methods: Research team and reflexivity  Paragraph 1 |
| Relationship with participants |  |
| 1. Relationship established | Methods: Research team and reflexivity  Paragraph 1 |
| 1. Participant knowledge of the interviewer | Methods: Research team and reflexivity  Paragraph 1 |
| 1. Interviewer characteristics | Methods: Research team and reflexivity  Paragraph 1 |
| **Domain 2: Study design**  Theoretical framework |  |
| 1. Methodological orientation and   Theory | Methods: Study design  Paragraph 2 |
| Participant selection |  |
| 1. Sampling | Methods: Study design  Paragraph 2 |
| 1. Method of approach | Methods: Study design  Paragraph 2 |
| 1. Sample size | Methods: Study design  Paragraph 2 |
| 1. Non-participation | Methods: Study design  Paragraph 2 |
| Setting |  |
| 1. Setting of data collection | Methods: Data collection  Paragraph 1 |
| 1. Presence of non-participants | Methods: Data collection  Paragraph 1 |
| 1. Description of sample | Methods: Data collection  Paragraph 1 & Supplemental 1 |
| Data Collection |  |
| 1. Interview guide | Methods: Data collection  Paragraph 1 & Supplemental 2 |
| 1. Repeat interviews | Methods: Data collection  Paragraph 1 |
| 1. Audio/visual recording | Methods: Data collection  Paragraph 1 |
| 1. Field notes | Methods: Data collection  Paragraph 1 |
| 1. Duration | Methods: Data collection  Paragraph 1 |
| 1. Data saturation | Methods: Data collection  Paragraph 1 |
| 1. Transcripts returned | Methods: Data collection  Paragraph 1 |
| **Domain 3: analysis and findings**  Data Analysis |  |
| 1. Number of data coders | Methods: Analysis and findings  Paragraph 1 |
| 1. Description of the coding tree | Methods: Analysis and findings  Paragraph 1 & Supplemental 3 |
| 1. Derivation of themes | Methods: Analysis and findings  Paragraph 1 |
| 1. Software | Methods: Analysis and findings  Paragraph 1 |
| 1. Participant checking | Methods: Data collection  Paragraph 1 |
| Reporting |  |
| 1. Quotations presented | Results: throughout  Supplemental 4  Supplemental 5  Supplemental 6  Supplemental 7 |
| 1. Data and findings consistent | Results: throughout |
| 1. Clarity of major themes | Results: throughout as indicated  Figure 1 – Theme 1  Figure 2 – Theme 2  Figure 3 – Theme 3  Figure 4 – Theme 4 |
| 1. Clarity of minor themes | Results: throughout as indicated  Figure 1 – Subtheme 1A & 1B  Figure 2 – Subtheme 2A, 2B & 2C  Figure 3 – Subtheme 3A & 3B  Figure 4 – Subtheme 4A, 4B, 4C & 4D |
